# Supplementary material for: CAR-mediated release of IL-10 increases the function of regulatory T cells: Relevance for future clinical application
Source: Mol Ther. 2026 Feb 6;34(5):2637–52. doi: 10.1016/j.ymthe.2026.02.001 (PMC13154253; doi:10.1016/j.ymthe.2026.02.001)
Supplement: Supplementary file 1 [file mmc1.pdf]

YMTHE, Volume *34*

Supplemental Information

CAR-mediated release of IL-10 increases the function of regulatory T cells: Relevance for future clinical application

Adeel Saleem, Qi Peng, Ziqin Tang, Yasmin R. Mohseni, Cristiano Scottà, Panicos Shangaris, Kimberly Smit, Wilbert P. Vermeij, Fadi Issa, Giovanna Lombardi, and Gilbert O. Fruhwirth

SUPPLEMENTAL FIGURES

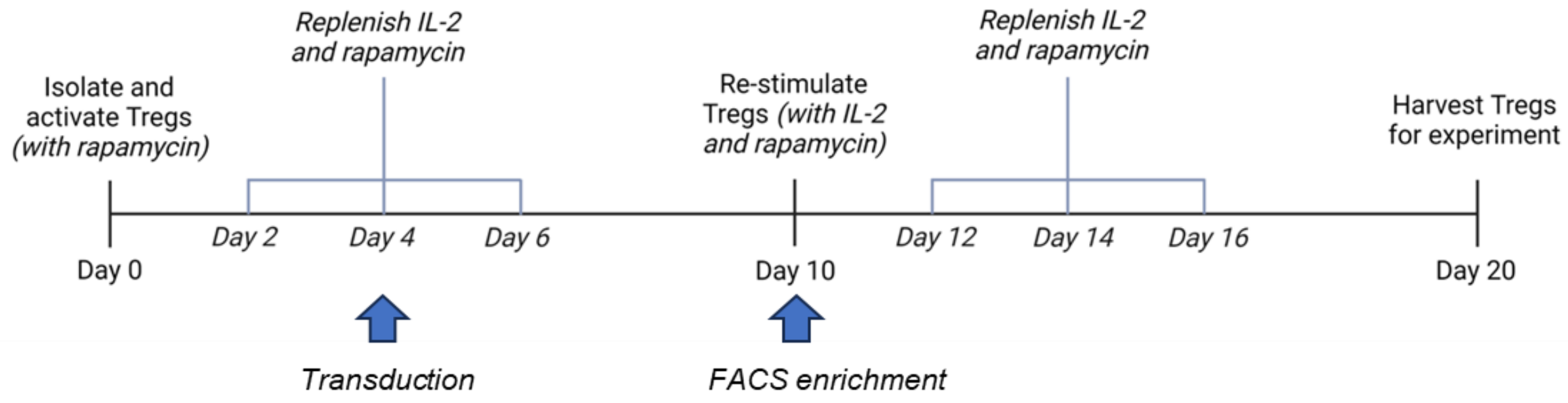

Figure S1. Scheme of Treg isolation, transduction, enrichment, and expansion. | IL-2 and rapamycin were replenished every two days after transduction with viral vectors. Transduction was performed on day 4 and FACS enrichment on day 10. Cells were harvested on day 20 for subsequent analyses or to use for *in vivo* experiments.

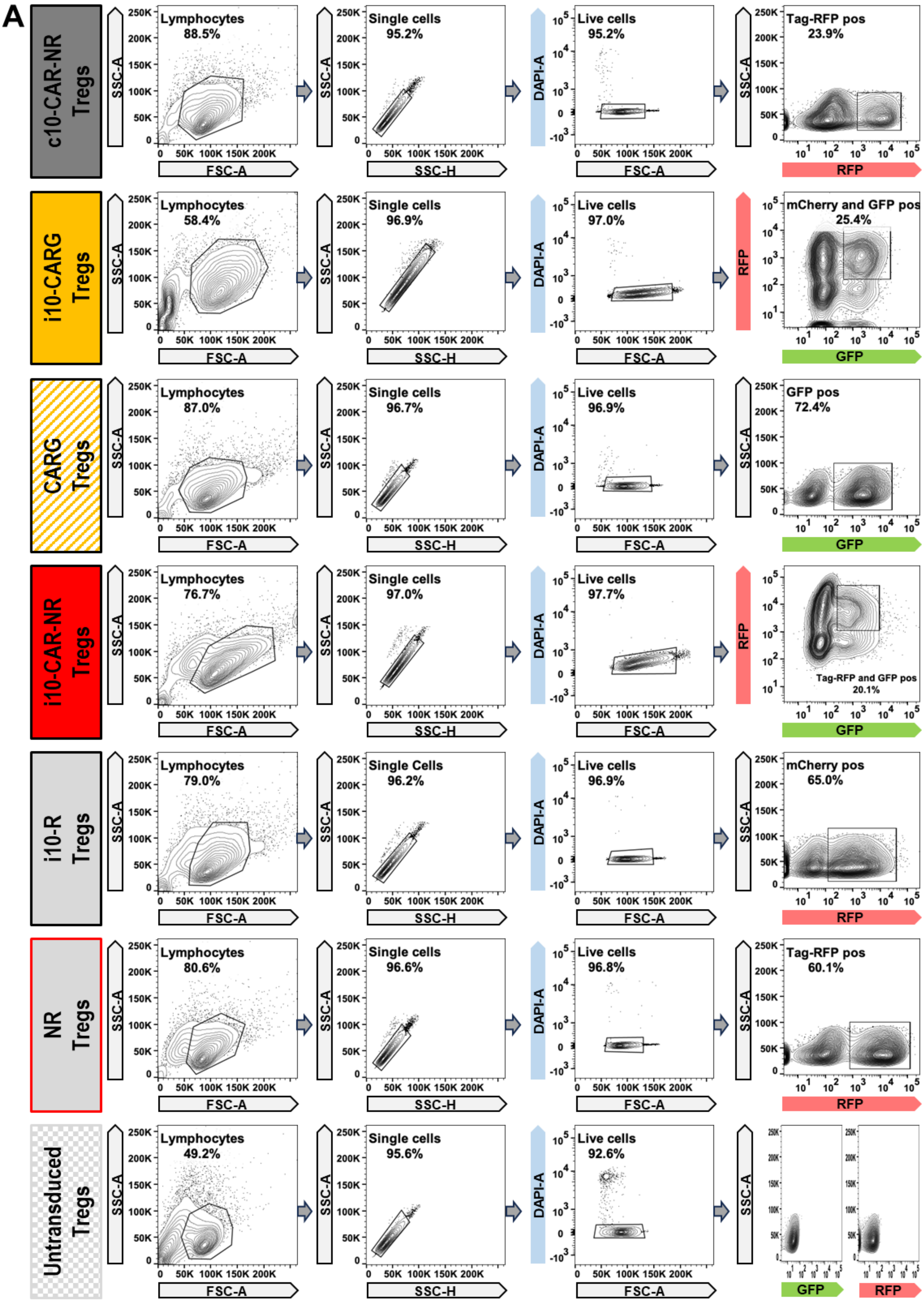
A
c10-CAR-NR Tregs
Lymphocytes 88.5%
Single cells 95.2%
Live cells 95.2%
Tag-RFP pos 23.9%
i10-CARG Tregs
Lymphocytes 58.4%
Single cells 96.9%
Live cells 97.0%
mCherry and GFP pos 25.4%
CARG Tregs
Lymphocytes 87.0%
Single cells 96.7%
Live cells 96.9%
GFP pos 72.4%
i10-CAR-NR Tregs
Lymphocytes 76.7%
Single cells 97.0%
Live cells 97.7%
Tag-RFP and GFP pos 20.1%
i10-R Tregs
Lymphocytes 79.0%
Single Cells 96.2%
Live cells 96.9%
mCherry pos 65.0%
NR Tregs
Lymphocytes 80.6%
Single cells 96.6%
Live cells 96.8%
Tag-RFP pos 60.1%
Untransduced Tregs
Lymphocytes 49.2%
Single cells 95.6%
Live cells 92.6%
SSC-A
FSC-A
SSC-H
DAPI-A
RFP
GFP

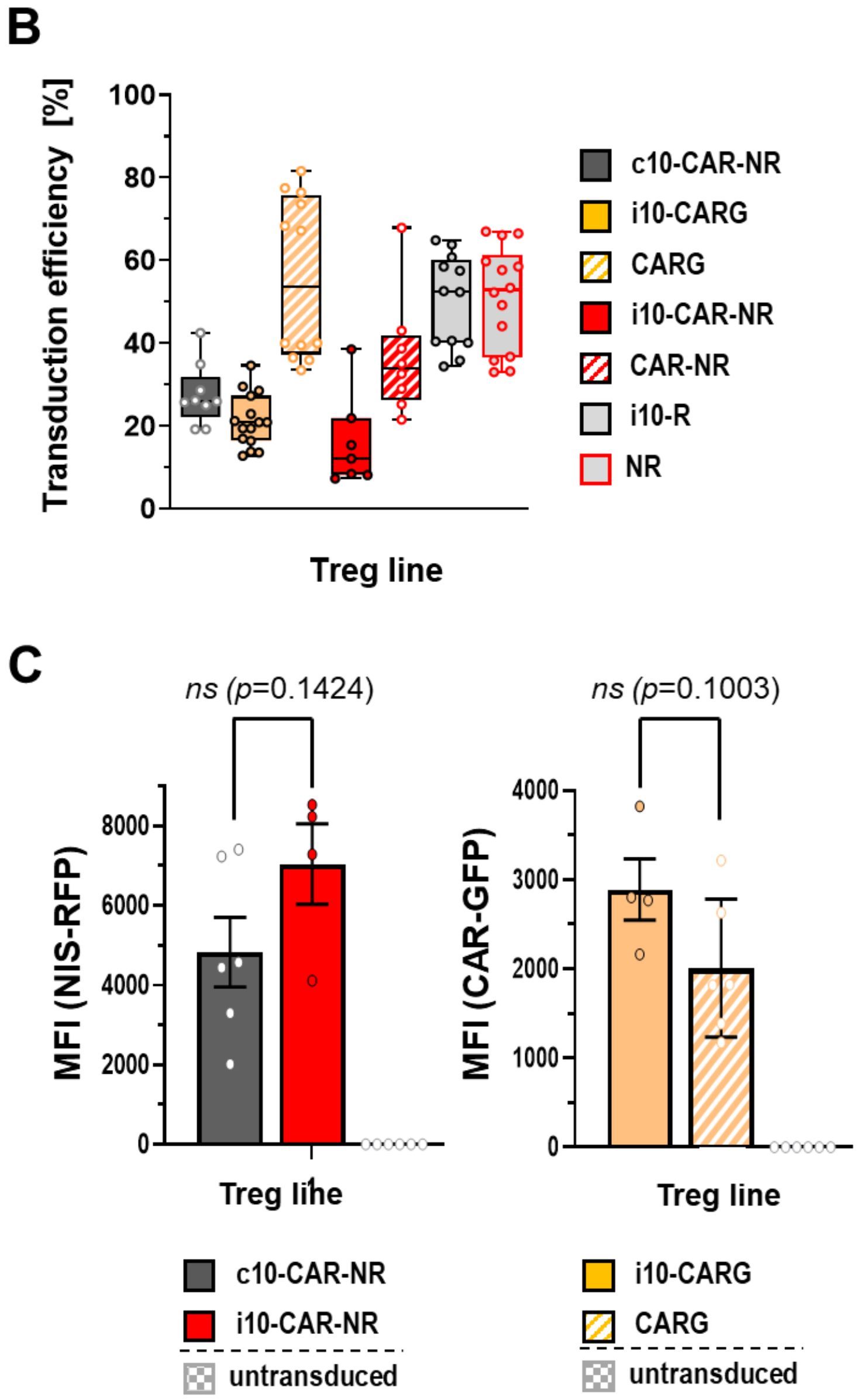

Figure S2. Representative gating strategy for evaluating transduction efficiencies. | (A) Representative flow cytometry plots showing the gating strategy for evaluating fluorescent protein expression (*cf.* Fig.1A) with examples from different engineered Treg types. Cells were analysed one week after transduction on day 10. **(B)** Cumulative transduction efficiency data based on the corresponding fluorescent protein reporter expression on day 10 and prior to FACS sorting of the various Treg populations. Box-an-Whisker plot shown with middle line indicating the medians and minima/maxima indicated by 'error bar' style lines. **(C)** CAR protein levels in the various CAR-Treg lines were determined by flow cytometry. *(Right)* i10-CARG and CARG Tregs expressed the anti-HLA-A*02 targeted CAR fused to GFP (see Fig.1A) and green fluorescence was a direct measure for CAR protein. *(Left)* In i10-CAR-NR and c10-CAR-NR Treg lines, anti-HLA-A*02 CAR expression was from the same transcript as NIS-RFP (see Fig.1), hence a surrogate for protein production from this transcript and therefore a measure for the CAR protein level. Mean fluorescence intensity (MFI) was determined on day 20 and is shown. Student's *t*-tests were used to evaluate any potential differences (α=0.05, two-tailed, heteroscedastic).

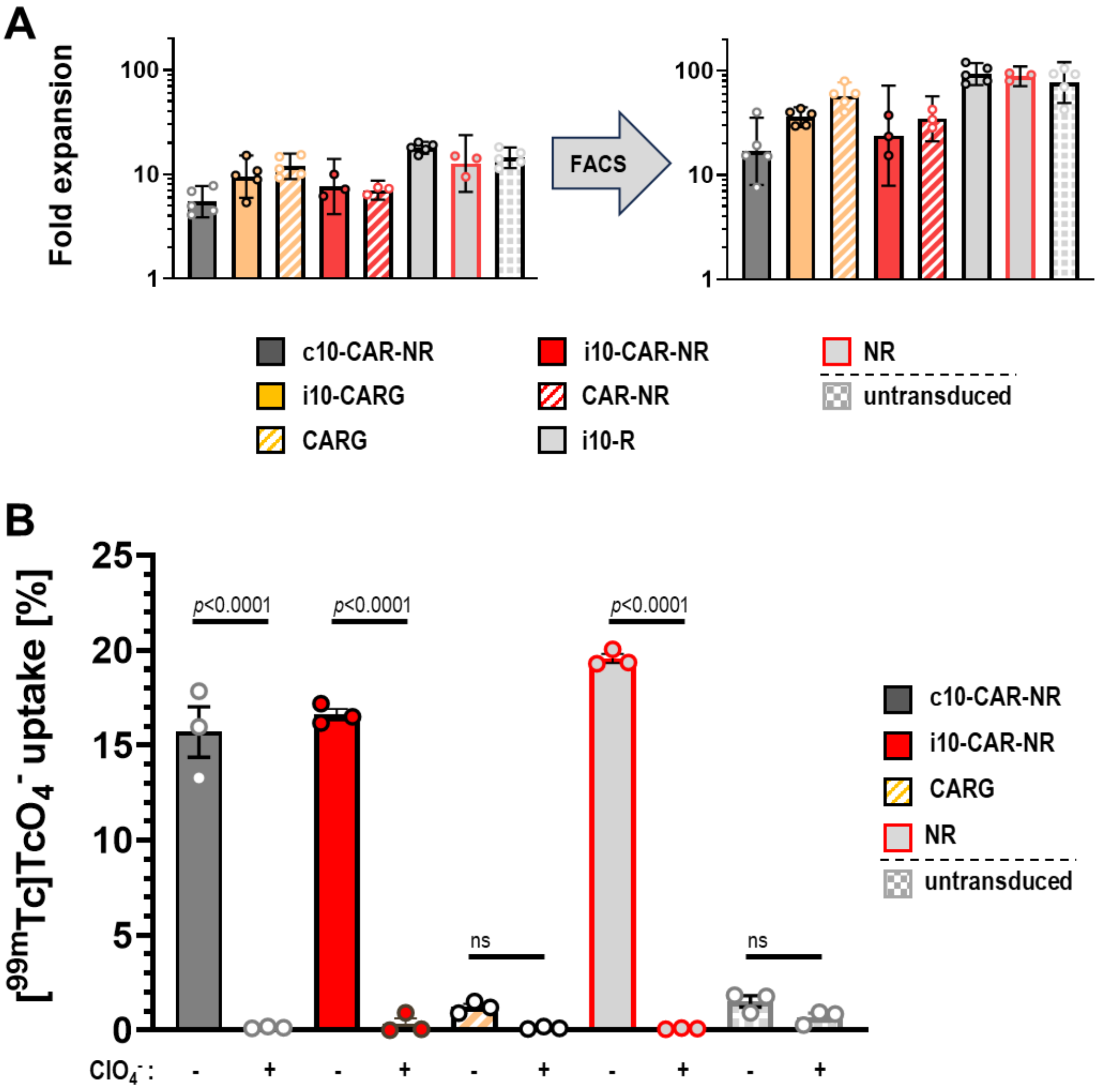

Figure S3. Expansion capacity and evaluation of radionuclide reporter function of engineered Tregs. | (A) Expansion capacity for indicated engineered Tregs for (*left*) the period between transduction and FACS sorting on day 10 and (*right*) for the period from FACS sorting until day 20. For timelines, see Figure S1. Geometric means are show with error bars representing 95% confidence intervals. **(B)** i10-CAR-NR, c10-CAR-NR, and NR Tregs (positive control) additionally contained the NIS-RFP reporter, which enables fluorescence applications but also future in vivo cell tracking by radionuclide imaging as demonstrated for Tregs before.[1] The function of the NIS portion of this dual–mode fusion reporter was determined by uptake of the NIS-specific substrate Tc-99m-pertechnetate (50kBq, 30min). The competitive substrate perchlorate was used to demonstrate that radiotracer uptake was specific to NIS-RFP. N=3 independent Treg batches from different donors with error bars representing SEM. Statistical significance was determined by Student's *t*-test (α=0.05, two-tailed, heteroscedastic) between sample pairs with and without perchlorate addition.

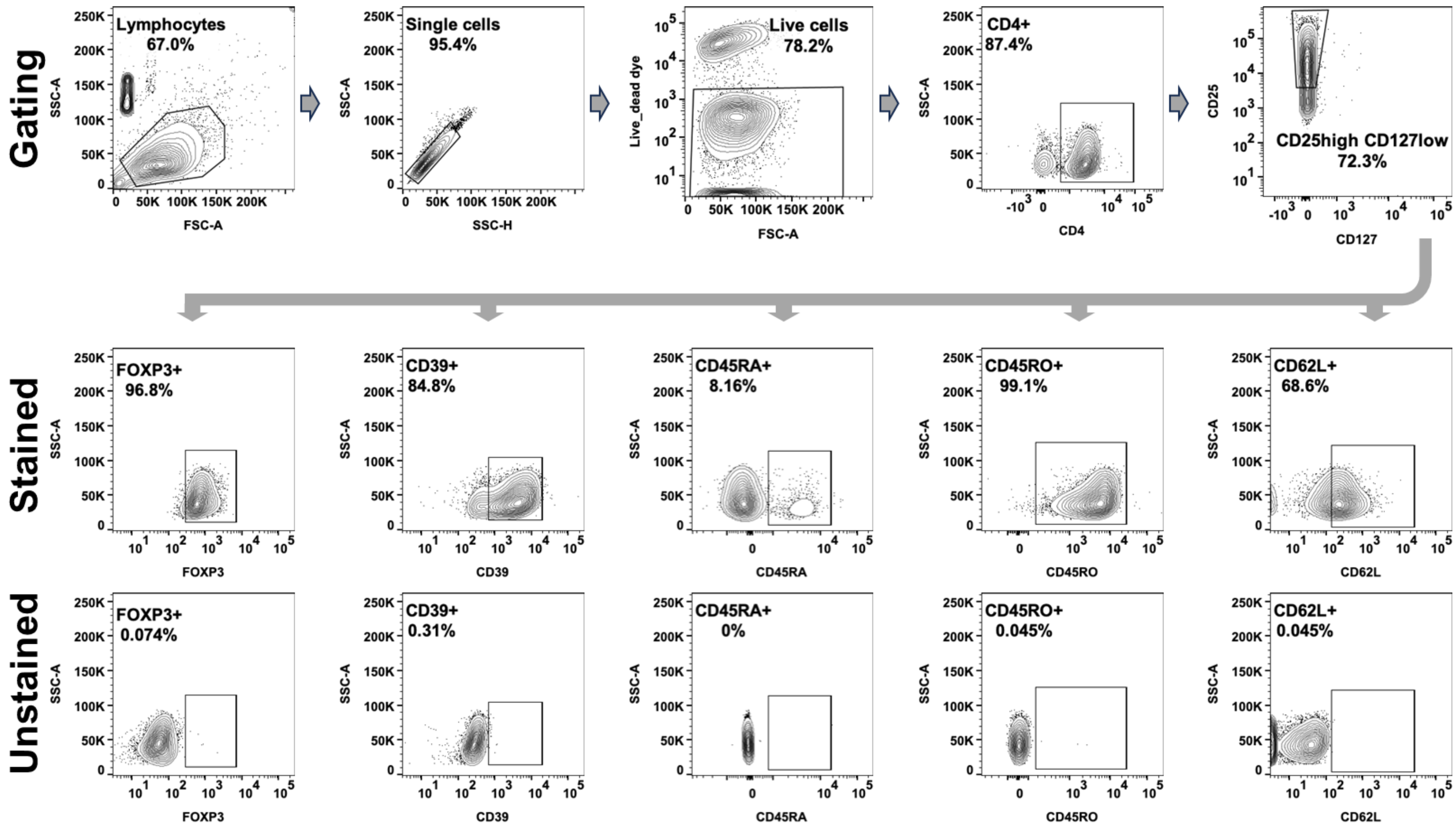

Figure S4. Representative Treg phenotyping example depicting gating strategy for indicated Treg markers. | Gating and subsequent representative results of stained and unstained samples for various Treg function and homing markers.

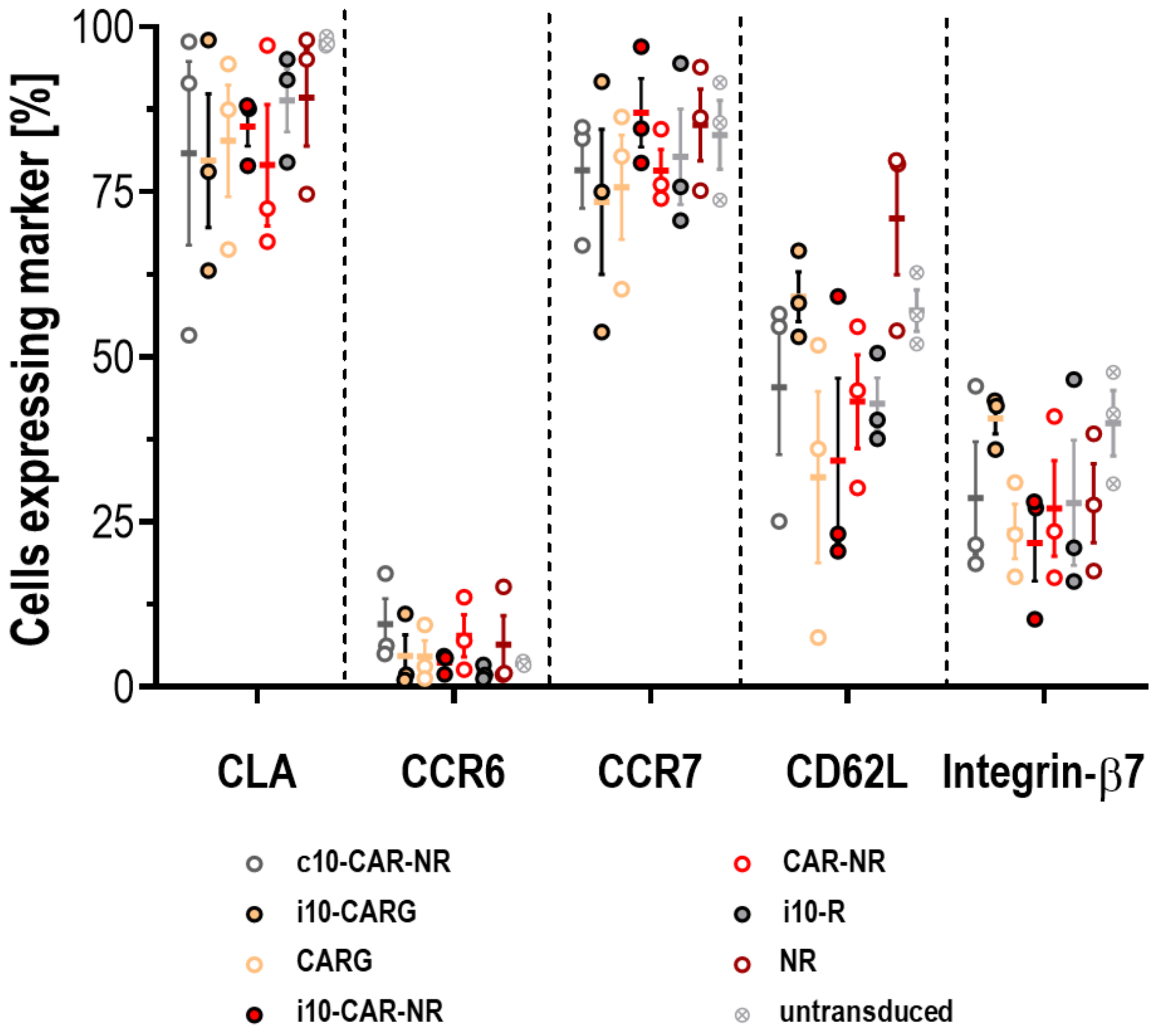

Figure S5. Surface expression of homing markers for Tregs. | Cumulative flow cytometry data for indicated marker expression in the various Treg types. N=3 independent Treg batches each with mean and error bars representing SEM.

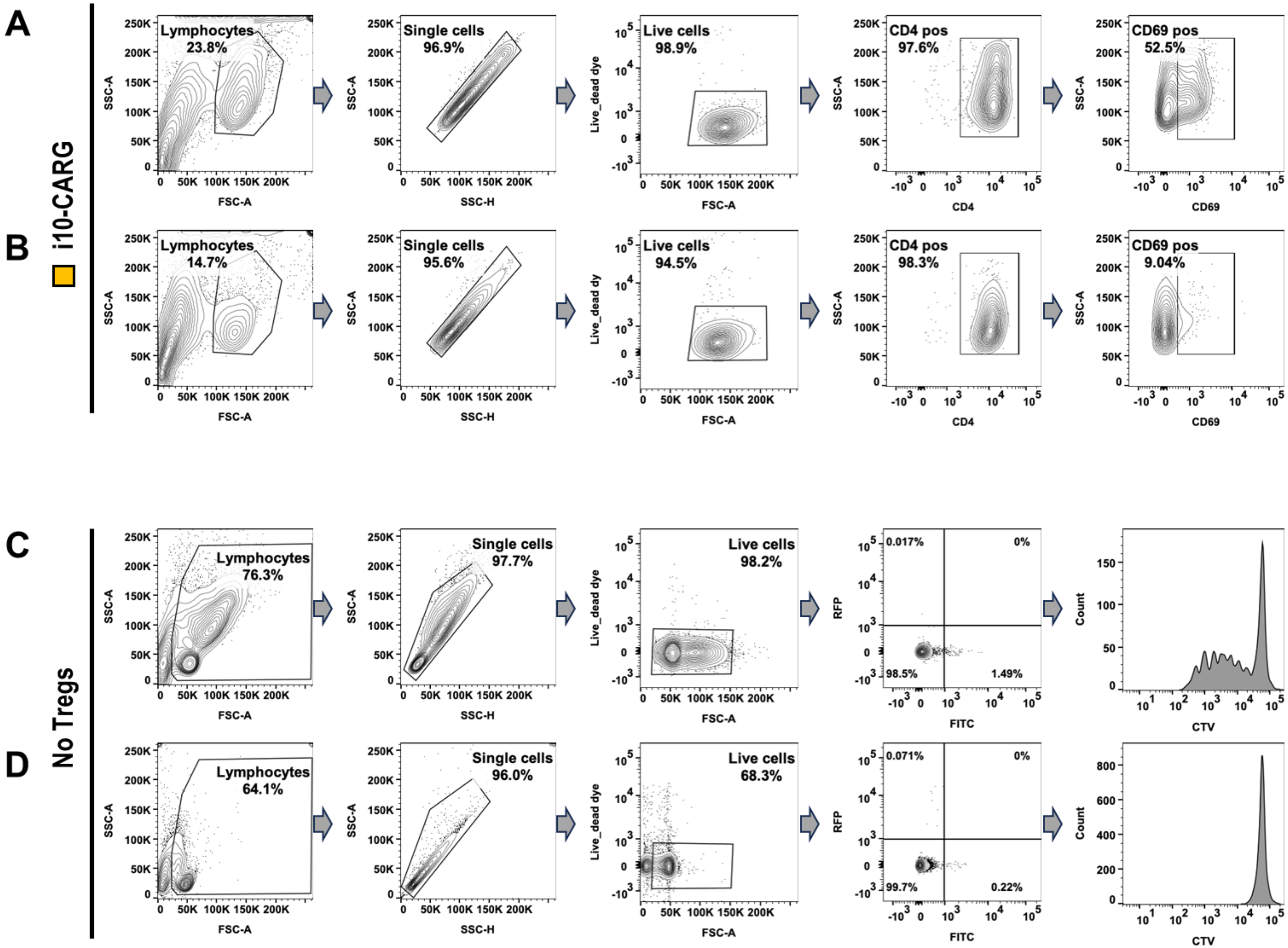
A
B
i10-CARG
Lymphocytes
23.8%
Single cells
96.9%
Live cells
98.9%
CD4 pos
97.6%
CD69 pos
52.5%
Lymphocytes
14.7%
Single cells
95.6%
Live cells
94.5%
CD4 pos
98.3%
CD69 pos
9.04%
C
D
No Tregs
Lymphocytes
76.3%
Single cells
97.7%
Live cells
98.2%
0.017%
0%
98.5%
1.49%
Lymphocytes
64.1%
Single cells
96.0%
Live cells
68.3%
0.071%
0%
99.7%
0.22%
SSC-A
FSC-A
SSC-H
Live_dead dye
CD4
CD69
RFP
FITC
Count
CTV

Figure S6. Representative gating strategy for determining the activation status of engineered Tregs and suppressive capacity towards Tconv. | i10-CARG Tregs were exposed to **(A)** the A2-CAR target antigen HLA-A*02 or **(B)** the mis-match antigen and Treg activation was measured by determining CD69 activation marker expression using flow cytometry (after 24h co-culture with corresponding B-LCLs). **(C)** Representative gating for Cell Trace Violet (CTV)-labelled Tconv that were stimulated with CD3/28 beads and consequently proliferated. **(D)** Representative gating for unstimulated CTV labelled Tconv, which did not proliferate. (C/D) are typical controls for assays that additionally contain Tregs (see Fig.3).

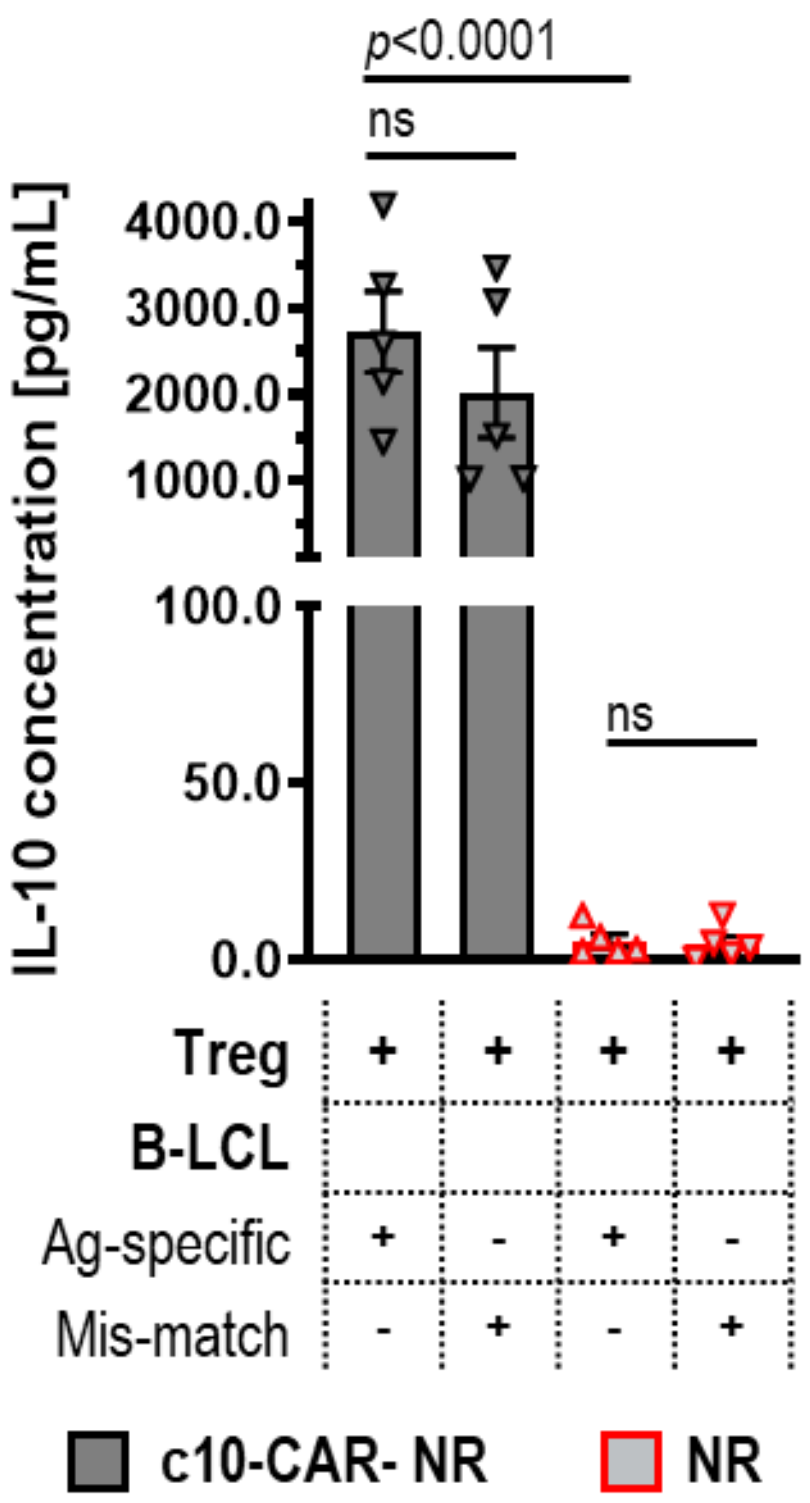

Figure S7. CD69 expression and IL-10 secretion data complementing Fig. 2C. | Indicated Treg types were co-cultured for 18h with B-LCL expressing either HLA-A*02 or the mis-match antigen HLA-A*01. IL-10 concentration was determined in co-culture supernatants of indicated engineered Tregs with HLA-A*02 B-LCLs or mis-match HLA-A*01 B-LCLs as indicated. N=5 Treg batches from different donors with error bars representing SEM. Statistical analyses in (A-B) were performed by one-way ANOVA with Tukey's multiple comparison correction and α=0.05 for all panels.

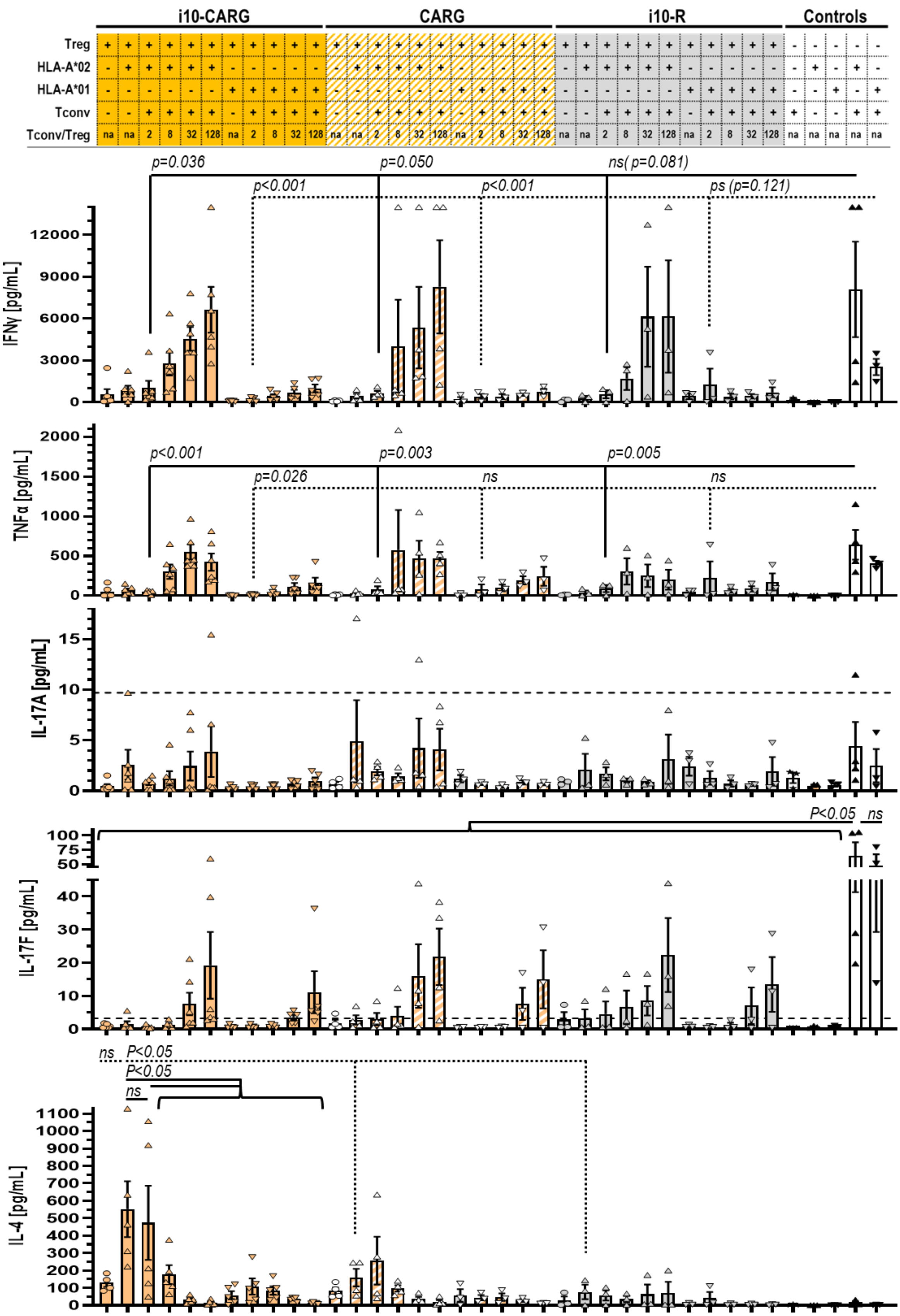

Figure S8. Cytokine secretion data supplementing suppression assays from Fig.3. | Secretion of additional indicated cytokines in the culture supernatants of various Treg suppression assays as assessed after 5 days of co-culture with autologous Tconv and

antigen presenting cells that were either HLA-A*02 positive B-LCLs (SPO) or HLA-A*02 negative B-LCLs (BM21). Cytokine bead array data are shown with means and error bars representing SEM; N ranged from 3-6. Cytokine secretion data from the supernatant of Tregs alone, Tregs with B-LCLs alone (without Tconv), B-LCLs alone, Tconv alone, and Tconv with B-LCLs (without Tregs) are included as controls. Statistical significance was tested by one-way ANOVA with multiple comparison correction, and the most relevant significant comparisons are visualised. The dashed line in the middle panel indicates the lower Limit of Measurement in the IL-17 assays.

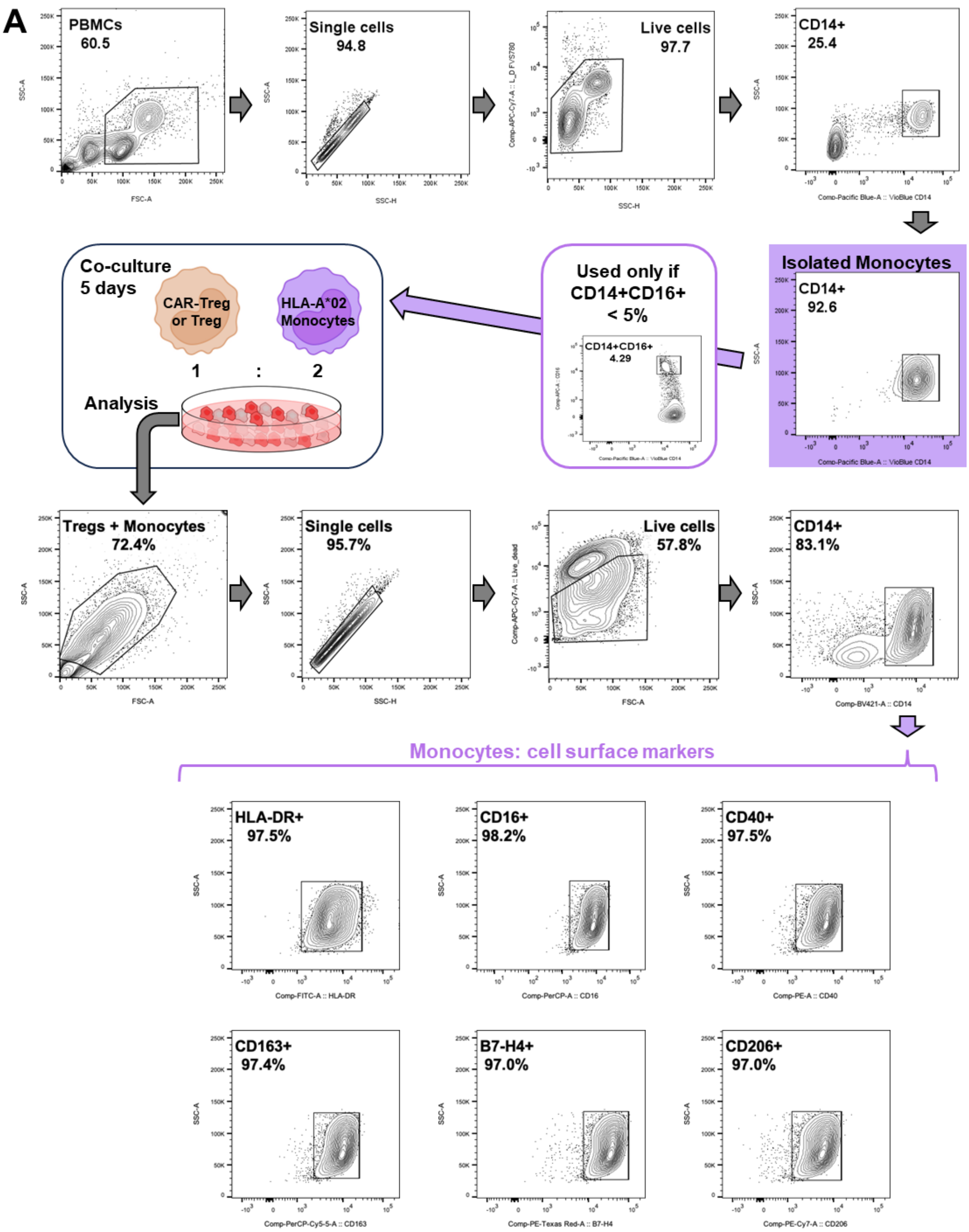
A
PBMCs
60.5
Single cells
94.8
Live cells
97.7
CD14+
25.4
Isolated Monocytes
CD14+
92.6
Used only if
CD14+CD16+
< 5%
CD14+CD16+
4.29
Co-culture
5 days
CAR-Treg
or Treg
HLA-A*02
Monocytes
1 : 2
Analysis
Tregs + Monocytes
72.4%
Single cells
95.7%
Live cells
57.8%
CD14+
83.1%
Monocytes: cell surface markers
HLA-DR+
97.5%
CD16+
98.2%
CD40+
97.5%
CD163+
97.4%
B7-H4+
97.0%
CD206+
97.0%

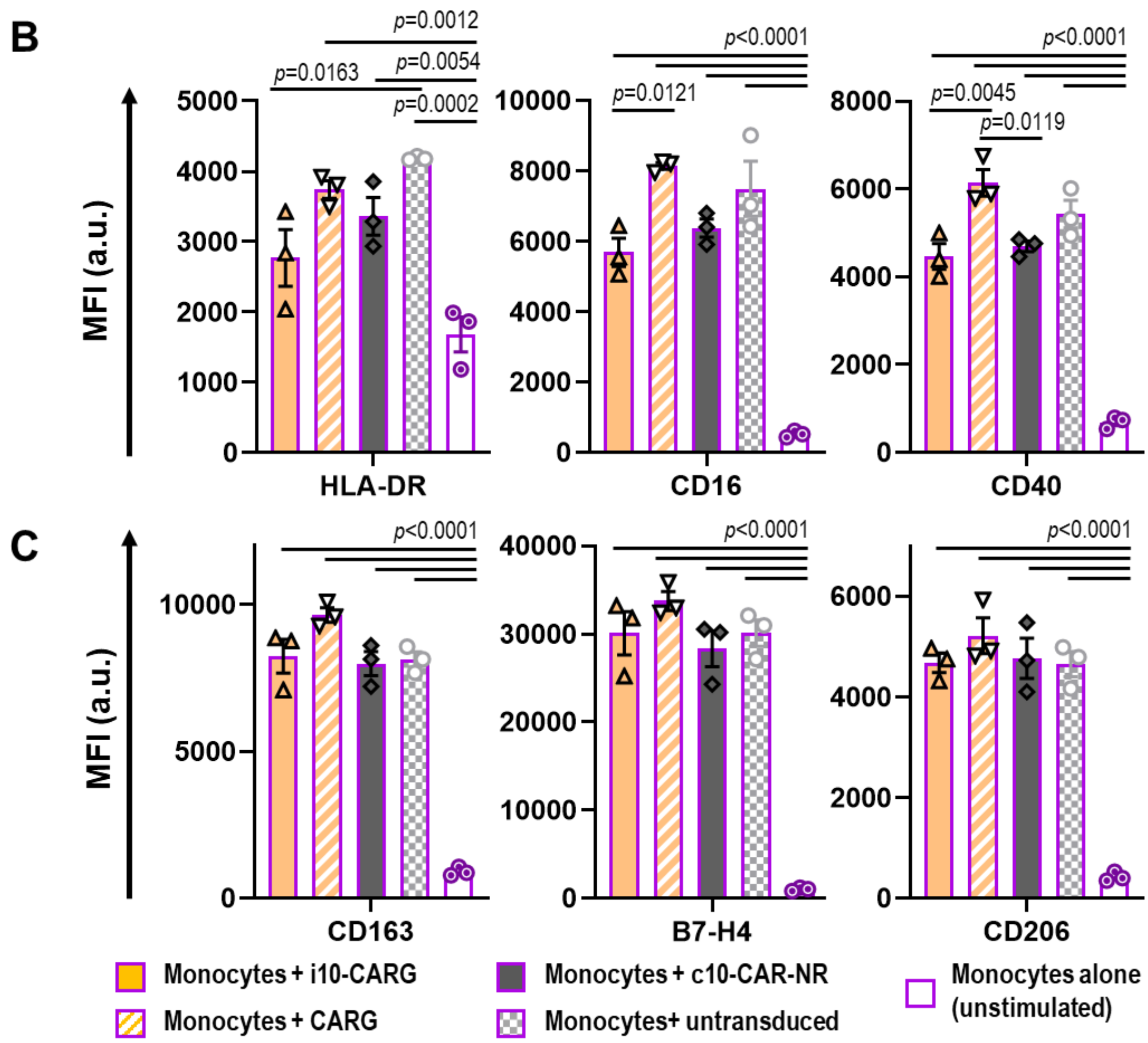

Figure S9. Effect of CAR-Tregs/Tregs on co-cultured HLA-A*02-positive monocytes | (A) Cartoon illustrating the monocyte:CAR-Treg/Treg co-culture assay. Flow cytometry panels at the top exemplify the analysis following the monocyte enrichment process (co-culture input). Flow panels at the bottom illustrate the gating undertaken for the analysis of monocytes after harvesting of the co-cultures. **(B)** Mean fluorescence intensity (MFI) values for indicated pro-inflammatory and **(C)** anti-inflammatory surface markers expressed on HLA-A*02 monocytes following co-culture with indicated Treg types. Unstimulated monocytes were included as a control. Cumulative results are shown from N=3 different experiments (*i.e.* different monocyte and Treg donors). Data are expressed as mean±SEM and with statistical analysis by one-way ANOVA (α<0.05) followed by Tukey's multiple comparison correction.

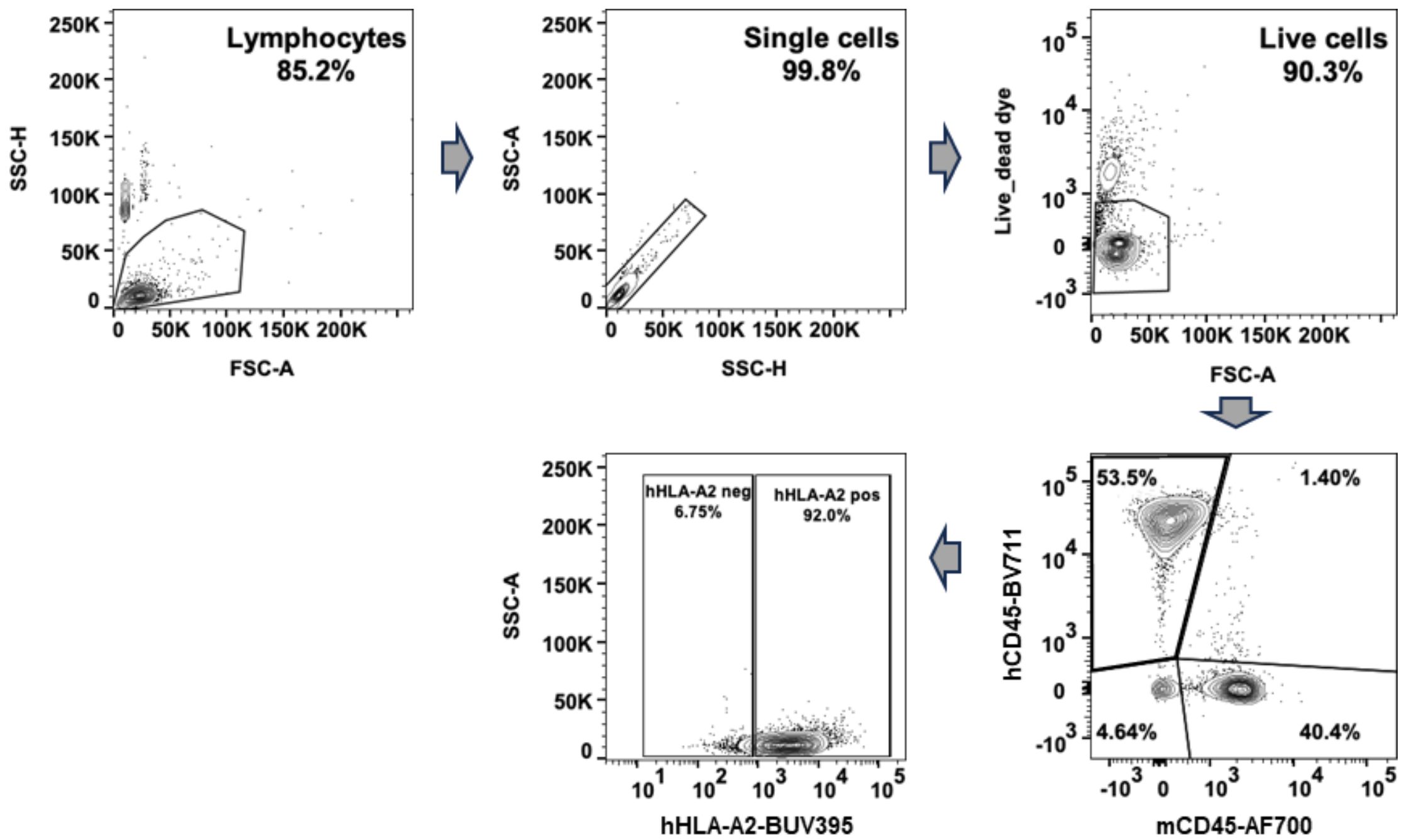

Figure S10. Representative gating strategy for determining HLA-A*02-positive CD45+ cells in peripheral blood of the xeno-GvHD model in Fig.4D. | Sample shown is from an animal that had received PBMCs and i10-CARG Tregs.

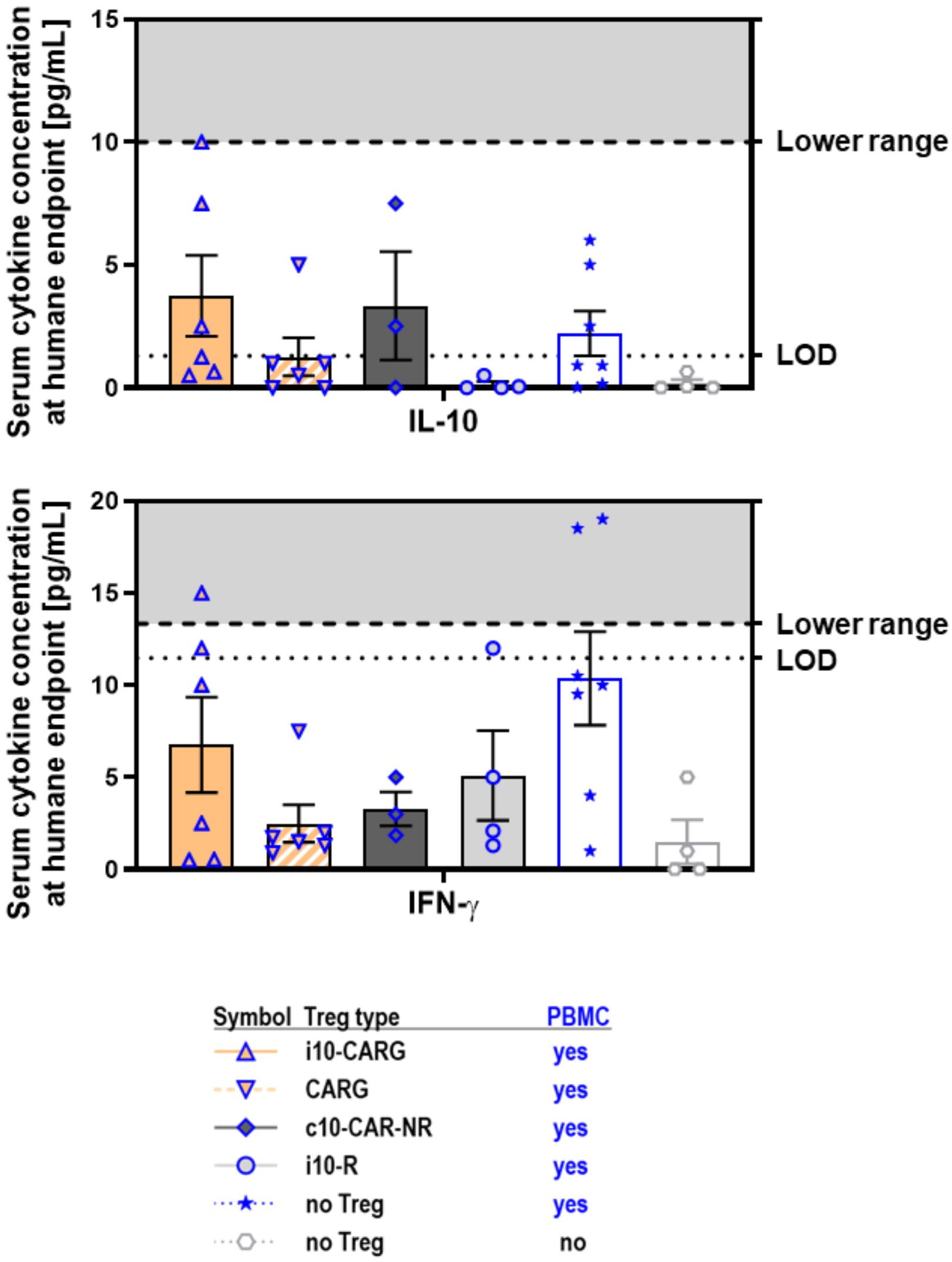

Figure S11. Serum cytokine determination at the time of animal sacrifice (human endpoint) from animals in Fig.4 | Graphs indicate the manufacturer's routine measurement range (*'Lower range' indicates its bottom end; dashed line*) and the limit of detection (*LOD; dotted line*) as obtained by our own measurements in control matrix samples; controls were sera from animals that had neither received PBMCs (no GvHD) nor Tregs and just an injection of saline. The number of data points is lower than in Fig.4, because readings of some serum samples were too low for a quantifiable instrument reading (*'not detectable')*. A noteworthy limitation of these measurements was that sampling was performed at the experimental humane endpoint, i.e. at the time of animal sacrifice, and not repeatedly at different time points ranging from therapeutic cell administration to the humane endpoint. This was due to animal license restrictions, which precluded serial sampling at volumes sufficient for repeat cytokine determination.

SUPPLEMENTAL TABLES

TableS1. List of fluorophores and optical flow cytometry settings as used for routine flow cytometric transduction and expansion analyses of the various Treg types (for constructs see Fig.1A); complements Figure S2 and S3A.

Marker	Fluorescent protein / dye	Antibody clone	Final concentration	Manufacturer	Product code	Laser wave-length (nm)	Emission Filter (nm)
Live/dead stain	DAPI	N/A	0.1µg/mL	ThermoFisher Scientific	62248	355	450/30
eGFP (expressed)	eGFP	N/A	N/A	N/A	N/A	488	530/30
mCherry (expressed)*	mCherry	N/A	N/A	N/A	N/A	561	610/20
mTagRFP (expressed)*	TagRFP	N/A	N/A	N/A	N/A	561	582/15

* never expressed in the same cell type.

TableS2. List of conjugated fluorescent antibodies and dyes as well as optical flow cytometry settings used for phenotype analyses. Fluorescent proteins as expressed by the various Treg types (*cf.* Fig.1A) are included. Antibodies in black font formed the panel for data shown in Fig.1B-D and Figure S4-S5.

Marker	Fluorescent protein / dye	Antibody clone	Dilution	Manufacturer	Product code	Laser wave-length (nm)	Emission Filter (nm)
Live/dead stain	Fixable Near-IR	N/A	1:1000	ThermoFisher Scientific	L10119	640	780/60
eGFP (expressed)	eGFP	N/A	N/A	N/A	N/A	488	530/30
mCherry (expressed)*	mCherry	N/A	N/A	N/A	N/A	561	610/20
mTagRFP (expressed)*	mTagRFP	N/A	N/A	N/A	N/A	561	582/15
CD4	BUV805	SK3	1:33	BD Biosciences	612888	355	780/60
CD25	PE-Vio-770	REA570	1:50	Miltenyi Biotec	130-133-749	561	780/60
CD127	BV786	HIL-7R-M21	1:50	BD Biosciences	563324	405	780/60
FOXP3	BV421	206D	1:20	Biolegend	320124	405	450/40
CD39	BV510	A1	1:20	Biolegend	328220	405	520/50
CD45RA	AF700	HI100	1:50	Biolegend	304120	640	730/45
CD45RO	BUV737	UCHL1	1:33	BD Biosciences	748368	355	740/35
CD62L	BV650	DREG-56	1:20	Biolegend	304832	405	660/20
CLA	BV605	HECA-452	1:20	BD Biosciences	563960	405	610/20
CCR6	BUV496	11A9	1:20	BD Biosciences	612948	355	515/30
CCR7	BV711	150503	1:20	Biolegend	353228	405	710/50
Integrin-β7**	PerCP-Cy5.5	FIB27	1:20	Biolegend	121008	488	710/50

* never expressed in the same cell type.

TableS3. List of conjugated fluorescent antibodies and dyes as well as optical flow cytometry settings used for activation and suppression analyses as in Fig.2A, Fig.3, and Figure S7. Fluorescent proteins as expressed by the various Treg types (*cf.* Fig.1A).

Marker	Fluorescent protein / dye	Antibody clone	Dilution	Manufacturer	Product code	Laser wave-length (nm)	Emission Filter (nm)
CTV	Cell Trace Violet	N/A	1:1000	ThermoFisher Scientific	C34557	405	450/40
Live/dead stain	Fixable Near-IR	N/A	1:1000	ThermoFisher Scientific	L10119	640	780/60
eGFP (expressed)	eGFP	N/A	N/A	N/A	N/A	488	530/30
mCherry (expressed)*	mCherry	N/A	N/A	N/A	N/A	561	610/20
mTagRFP (expressed)*	TagRFP	N/A	N/A	N/A	N/A	561	582/15
CD4	AF647	SK3	1:33	Biolegend	344636	640	670/30
CD25	PE-Vio-770	REA570	1:50	Miltenyi Biotec	130-133-749	561	780/60
CD69	PerCP	FN50	1:20	Biolegend	310928	488	710/50

* never expressed in the same cell type.

TableS4. List of conjugated fluorescent antibodies and dyes as well as optical flow cytometry settings used for monocyte-Treg co-culture experiments as in Figure S9. Fluorescent proteins were only expressed by various Treg types (*cf.* Fig.1A) but not by monocytes, hence are not listed here as CD14 gating always selected the monocyte population prior to cell surface marker analyses.

Marker	Fluorescent protein / dye	Antibody clone	Dilution	Manufacturer	Product code	Laser wave-length (nm)	Emission Filter (nm)
Live/dead stain	Fixable Near-IR	N/A	1:1000	ThermoFisher Scientific	L10119	640	780/60
CD14	BV421	MoP9	1:20	BD Biosciences	563743	405	450/40
HLA-DR	Alexa fluor 488	L243	1:20	Biolegend	307619	488	530/30
CD16	PerCP	3G8	1:20	Biolegend	302029	488	670/30
CD40	PE	5C3	1:20	Biolegend	334307	561	582/15
CD163	PerCP-Vio700	REA812	1:50	Miltenyi Biotec	130-112-291	488	710/50
B7-H4	PE/Dazzle	MIH43	1:20	Biolegend	358112	561	610/20
CD206	PE/Cy7	15-2	1:20	Biolegend	321101	561	780/60

TableS5. List of conjugated fluorescent antibodies and dyes as well as optical flow cytometry settings used for *ex vivo* engraft-ment analyses in peripheral blood as in Fig.4D and Figure S10. Fluorescent proteins as expressed by various Treg types (*cf.* Fig.1A).

Marker	Fluorescent protein / dye	Antibody clone	Dilution	Manufacturer	Product code	Laser wave-length (nm)	Emission Filter (nm)
Live/dead stain	Fixable Near-IR	N/A	1:1000	ThermoFisher Scientific	L10119	640	780/60
eGFP (expressed)	eGFP	N/A	N/A	N/A	N/A	488	530/30
mCherry (expressed)*	mCherry	N/A	N/A	N/A	N/A	561	610/20
mTagRFP (expressed)*	TagRFP	N/A	N/A	N/A	N/A	561	582/15
mCD45	AF700	30-F11	1:50	Biolegend	103128	640	730/45
hCD45	BV711	HI30	1:20	Biolegend	304050	405	710/50
hHLA-A2	BUV395	BB7.2	1:50	BD Biosciences	740313	355	379/28

* never expressed in the same cell type.

TableS6. List of exact *p*-values for individual comparisons in Fig.3B. Exact *p*-values were calculated by two-way ANOVA using Tukey's multiple comparison correction using GraphPad Prism software.

B-LCL: HLA-A*02			
**Tconv:Treg ratio**	**Tukey's multiple comparisons test**	***p*-value**	**Summary**
1	CAR-Treg vs. CAR-IL10i-Tregs	0.6391	ns
	CAR-Treg vs. Group C	0.0071	**
	CAR-IL10i-Tregs vs. Group C	0.0004	***
2	CAR-Treg vs. CAR-IL10i-Tregs	0.3696	ns
	CAR-Treg vs. Group C	0.0183	*
	CAR-IL10i-Tregs vs. Group C	0.0003	***
4	CAR-Treg vs. CAR-IL10i-Tregs	0.4743	ns
	CAR-Treg vs. Group C	0.0304	*
	CAR-IL10i-Tregs vs. Group C	0.0011	**
8	CAR-Treg vs. CAR-IL10i-Tregs	0.9846	ns
	CAR-Treg vs. Group C	0.0042	**
	CAR-IL10i-Tregs vs. Group C	0.0026	**
16	CAR-Treg vs. CAR-IL10i-Tregs	0.946	ns
	CAR-Treg vs. Group C	0.0137	*
	CAR-IL10i-Tregs vs. Group C	0.0057	**
32	CAR-Treg vs. CAR-IL10i-Tregs	0.9814	ns
	CAR-Treg vs. Group C	0.0786	ns
	CAR-IL10i-Tregs vs. Group C	0.116	ns
64	CAR-Treg vs. CAR-IL10i-Tregs	0.9929	ns
	CAR-Treg vs. Group C	0.1171	ns
	CAR-IL10i-Tregs vs. Group C	0.0924	ns
128	CAR-Treg vs. CAR-IL10i-Tregs	0.8198	ns
	CAR-Treg vs. Group C	0.4242	ns
	CAR-IL10i-Tregs vs. Group C	0.7889	ns
256	CAR-Treg vs. CAR-IL10i-Tregs	0.8998	ns
	CAR-Treg vs. Group C	0.8533	ns
	CAR-IL10i-Tregs vs. Group C	0.5955	ns

B-LCL: HLA-A*01			
**Tconv:Treg ratio**	**Tukey's multiple comparisons test**	***p*-value**	**Summary**
all ratios	All comparison had *p*-values	>0.05	ns

SUPPLEMENTAL REFERENCES

1. Jacob, J., Nadkarni, S., Volpe, A., Peng, Q., Tung, S.L., Hannen, R.F., Mohseni, Y.R., Scotta, C., Marelli-Berg, F.M., Lechler, R.I., Smyth, L.A., et al. (2021). Spatiotemporal in vivo tracking of polyclonal human regulatory T cells (Tregs) reveals a role for innate immune cells in Treg transplant recruitment. Mol Ther Methods Clin Dev *20*, 324-336. 10.1016/j.omtm.2020.12.003.
