# Supplementary material for: CAR-mediated release of IL-10 increases the function of regulatory T cells: Relevance for future clinical application
Source: Mol Ther. 2026 Feb 6;34(5):2637–52. doi: 10.1016/j.ymthe.2026.02.001 (PMC13154253; doi:10.1016/j.ymthe.2026.02.001)
Supplement: Supplementary file 2 [file mmc2.pdf]

Molecular Therapy
Original Article

CAR-mediated release of IL-10 increases the function of regulatory T cells: Relevance for future clinical application

Adeel Saleem,[1,2,3] Qi Peng,[1,2] Ziqin Tang,[3,4] Yasmin R. Mohseni,[1,2] Cristiano Scottà,[2,5] Panicos Shangaris,[2,6] Kimberly Smit,[3,4] Wilbert P. Vermeij,[3,4] Fadi Issa,[7,8] Giovanna Lombardi,[2,9] and Gilbert O. Fruhwirth[1,9]

[1]Imaging Therapies and Cancer Group, School of Cancer and Pharmaceutical Sciences, King's College London, London SE1 9RT, UK; [2]Peter Gorer Department, School of Immunology and Microbial Sciences, King's College London, London SE1 9RT, UK; [3]Princess Maxima Center for Pediatric Oncology, 3584 CS Utrecht, the Netherlands; [4]Oncode Institute, 3521 AL Utrecht, the Netherlands; [5]Department of Biosciences, Centre for Inflammation Research and Translational Medicine, College of Health, Medicine and Life Sciences, Brunel University, London UB8 3PH, UK; [6]Department of Women and Children's Health, School of Life Course and Population Sciences, King's College London, London SE1 9RT, UK; [7]Chinese Academy of Medical Sciences Oxford Institute, University of Oxford, Oxford OX3 9DU, UK; [8]Transplantation Research and Immunology Group, Nuffield Department of Surgical Sciences, University of Oxford, Oxford OX3 9DU, UK

Regulatory T cell (Treg) therapy emerges for various indications associated with a breakdown of immune tolerance. Antigen-specific chimeric antigen receptor (CAR) Tregs are frontrunners for transplantation and autoimmune diseases and are currently being clinically evaluated. We aimed to link CAR-antigen engagement with immunosuppressive cargo release into the local microenvironment to boost efficacy and reduce side effects. We used our HLA-A*02 CAR and immunosuppressive interleukin-10 (IL-10) as model components to generate human CAR Tregs that release IL-10 upon CAR engagement. These were compared to CAR Tregs with constitutive or no IL-10 expression by evaluating phenotypes, antigen-specific IL-10 release, and suppression of effector cell proliferation *in vitro* and performance *in vivo* in a humanized xenogeneic graft-versus-host disease (xeno-GvHD) model. We demonstrated successful multi-construct engineering of CAR Tregs, which released upon CAR engagement 2.5-fold more IL-10 than CAR Tregs lacking the corresponding antigen-specific IL-10 secretion module. Neither phenotype nor function was affected by expressing this module. In the xeno-GvHD model, we showed the beneficial effect of IL-10 release, particularly evident when compared to constitutive IL-10 expression that impaired CAR-Treg efficacy. We provide first proof-of-principle for engineering human CAR Tregs to release an immunosuppressive cytokine upon CAR engagement. This approach will both enhance the potency of CAR Tregs at the intended target sites and limit their off-target effects.

INTRODUCTION

Regulatory T cells (Tregs) are a specialized subset of T cells that express CD4 and CD25 surface markers along with the transcription factor FOXP3 and exhibit low levels of CD127.[1] Tregs are essential for maintaining self-tolerance and dampening immune responses.[2] Currently, Tregs are being clinically evaluated for their potential in adoptive cell therapies aimed at preventing transplant rejection and treating autoimmune disorders, with several phase 1/2 clinical trials being conducted.[3] We have previously reported two clinical trials that demonstrated the safety of adoptive polyclonally expanded Tregs in liver transplant patients (the ThRIL study,[4] NCT02166177) and kidney transplant patients (the ONE study,[5,6] NCT02129881). Both studies confirmed that Treg therapy was safe and well tolerated, with no serious adverse events, and showed initial signs of effectiveness in reducing inflammation and preventing acute rejection.[4–6] However, several studies showed that donor-specific mouse and human Tregs are more effective than polyclonal Tregs in preclinical transplant models.[7–9]

One approach to confer antigen specificity to Tregs is by genetically engineering them to express a chimeric antigen receptor (CAR) that is tailored for the desired alloantigen. This approach aims to improve both the targeting and efficacy of adoptively transferred Tregs. Several studies reported on successful engineering of Tregs with alloantigen-specific CARs demonstrating potential efficacy benefits.[10–12] A significant benefit of using CARs to provide Tregs with antigen specificity is that it bypasses the limitations of major histocompatibility complex (MHC) restrictions, and the Tregs can be targeted to MHC class I antigens. We and others have engineered human

Received 15 May 2025; accepted 2 February 2026;
https://doi.org/10.1016/j.ymthe.2026.02.001.

[9]Senior author

Correspondence: Giovanna Lombardi, School of Immunology and Microbial Sciences, King's College London, Guy's Hospital, Tower Wing, 5th Floor, Great Maze Pond, London SE1 9RT, UK.
E-mail: giovanna.lombardi@kcl.ac.uk
Correspondence: Gilbert O. Fruhwirth, School of Cancer and Pharmaceutical Sciences, King's College London, New Hunt's House, 2nd Floor, Great Maze Pond, London SE1 1UL, UK.
E-mail: gilbert.fruhwirth@kcl.ac.uk

Published by Elsevier Inc. on behalf of The American Society of Gene and Cell Therapy.
This is an open access article under the CC BY license (http://creativecommons.org/licenses/by/4.0/).

Tregs to express a CAR that targets HLA-A*02, and these A2-specific CAR Tregs have shown enhanced functionality compared to polyclonal Tregs both *in vitro* and *in vivo* in humanized mouse models.[11–13] The success of preclinical studies on CAR Tregs has led to two current clinical trials exploring their safety and therapeutic potential in reducing transplant rejection (LIBERATE, NCT05234190 and STEADFAST, NCT04817774).

Exploiting synthetic biology beyond CAR engineering has the potential to unlock several additional benefits. While in oncology and in the context of CAR-engineered effector T cells, additional components were expressed, for example, cytokines,[14,15] antibody fragments,[16,17] suicide genes,[18] or clinically compatible imaging reporters,[19,20] similar efforts and potential benefits currently lag in Treg therapy as it is an emerging therapy modality. We previously found that packing additional cargoes into transgene cassettes for the engineering of CAR Tregs was possible without impairing phenotype and function, which we demonstrated for both the cytokine interleukin-10 (IL-10) and a clinically compatible imaging reporter.[21] In this previous work, we also showed that high levels of IL-10 expression can enhance the suppressive ability of CAR Tregs *in vitro*. However, since IL-10 has pleiotropic effects, such as promoting the activity of natural killer cells or effector CD8$^+$ T cells,[22,23] we hypothesized that it may be more beneficial to avoid constitutive high-level IL-10 expression in Tregs, which may mimic systemic administration.

Building on our previous anti-HLA-A*02 CAR-Treg work including above efforts to constitutively express and secrete IL-10 cargo in CAR Tregs,[21] our goal here was to create an inducible system in which cargo expression/release occurs on CAR-antigen engagement only. We envisaged to equip CAR Tregs with this feature and thereby only secrete IL-10 locally at the target site, boosting therapeutic efficacy and limiting off-target effects.

RESULTS

Generation of CAR Tregs with an inducible IL-10 expression module and reporter genes

We generated lentiviral transgene cassettes to express IL-10 under the endogenous control of the nuclear factor of activated T cells (NFAT) promoter alongside our previously reported anti-HLA-A2-CAR (A2-CAR[11]). This second-generation A2-CAR contains a C-terminal CD3ζ domain which, upon antigen binding to the A2-CAR, triggers a downstream NFAT transcription-factor-mediated response. To streamline Treg engineering and downstream experimentation at this preclinical development stage, we included constitutively expressed fluorescent reporter proteins (Figure 1A: red and green fluorescent protein [RFP and GFP] in *R_i10* and *G_i10* plasmids, respectively). We adopted a double transduction strategy to keep overall provirus sizes within lentiviral packaging limits while achieving reasonably high virus titers. Lentiviruses produced were then combined with lentiviruses for transfer of A2-CAR-encoding constructs. We combined lentiviruses produced from the *R_i10* plasmid (constitutive RFP with inducible IL-10) with lentiviruses produced from the *A2-CAR-GFP* plasmid (encoding A2-CAR fused to GFP[11]) yielding CAR Tregs that included NFAT-driven IL-10 expression while expressing both cytosolic RFP alongside the A2-CAR-GFP fusion protein localized to the plasma membrane (Figure 1A: "i10-CARG" Tregs; yellow). Analogously, we engineered CAR Tregs that expressed the A2-CAR alongside the radionuclide reporter sodium iodide symporter fused to TagRFP (NIS-RFP; expressed in the plasma membrane and enabling future *in vivo* cell tracking) alongside IL-10 under NFAT control and expressing cytosolic GFP (Figure 1A: "i10-CAR-NR" Tregs; red). In addition, we generated the following Treg types as controls for various subsequent experiments: (1) Tregs constitutively expressing IL-10, the A2-CAR, and the NIS-RFP reporter ("c10-CAR-NR" Tregs[21]; dark gray); (2) Tregs expressing IL-10 under NFAT control alongside constitutive RFP expression ("i10-R" Tregs; light gray); (3) Tregs constitutively expressing only the A2-CAR-GFP fusion protein ("CARG" Treg[11]; yellow stripes); (4) Tregs constitutively expressing the A2-CAR alongside the NIS-RFP dual-mode reporter ("CAR-NR"[21]; red stripes); and (5) Tregs constitutively expressing the NIS-RFP reporter only ("NR"[21]; light gray/red frame) (for constructs and resulting Tregs, see Figure 1A).

Human Tregs were isolated as previously reported,[21] transduced, and expanded polyclonally *in vitro* in the presence of IL-2 and rapamycin (Figure S1). Lentiviral transduction efficiencies were evaluated based on fluorescent protein expression (Figures S2A and S2B) before transduced cells were enriched through fluorescence-activated cell sorting (FACS) on day 10, followed by further expansion until day 20 and analysis for fluorescence marker presence. All Treg preparations were >74.6% transduced (range: 74.6%–94.7%) (Figure 1B), with CAR-expressing Treg lines expressing comparable CAR protein levels (Figure S2C). Tregs were expanded prior to FACS and thereafter, with corresponding expansion levels shown in Figure S3A. Moreover, the different experimental and control Treg types contained various reporter molecules. These were either cytosolic fluorescent proteins, a GFP fused to the A2-CAR (in CARG Tregs), or, in some cases, NIS-RFP. Fluorescent protein expression was assessed by flow cytometry (e.g., Figures 1B and S2), and all engineered Treg types behaved as expected. For NIS-RFP reporter expressing Tregs, NIS function was evaluated through uptake of the NIS-specific radiotracer Tc-99m pertechnetate ($^{99m}TcO_4^-$) and demonstrated functional NIS-RFP expression in all cases (Figure S3B).

A2-CAR Tregs engineered with the inducible IL-10 expression module retain their phenotype

Next, we evaluated the impact of lentiviral transduction, FACS, and subsequent expansion on Treg phenotypes compared to untransduced Tregs. We confirmed there were no differences at the end of the 20-day culture period between various Treg preparations, and average FOXP3 expression was over 90% (Figure 1C; representative gating shown in Figure S4). All engineered Treg preparations presented on day 20 with low CD45RA and high CD45RO expression as expected (Figure 1D), and various Treg homing markers were evaluated (CLA, CCR7 >> CD62L, integrin β7 > CCR6; Figure S5).

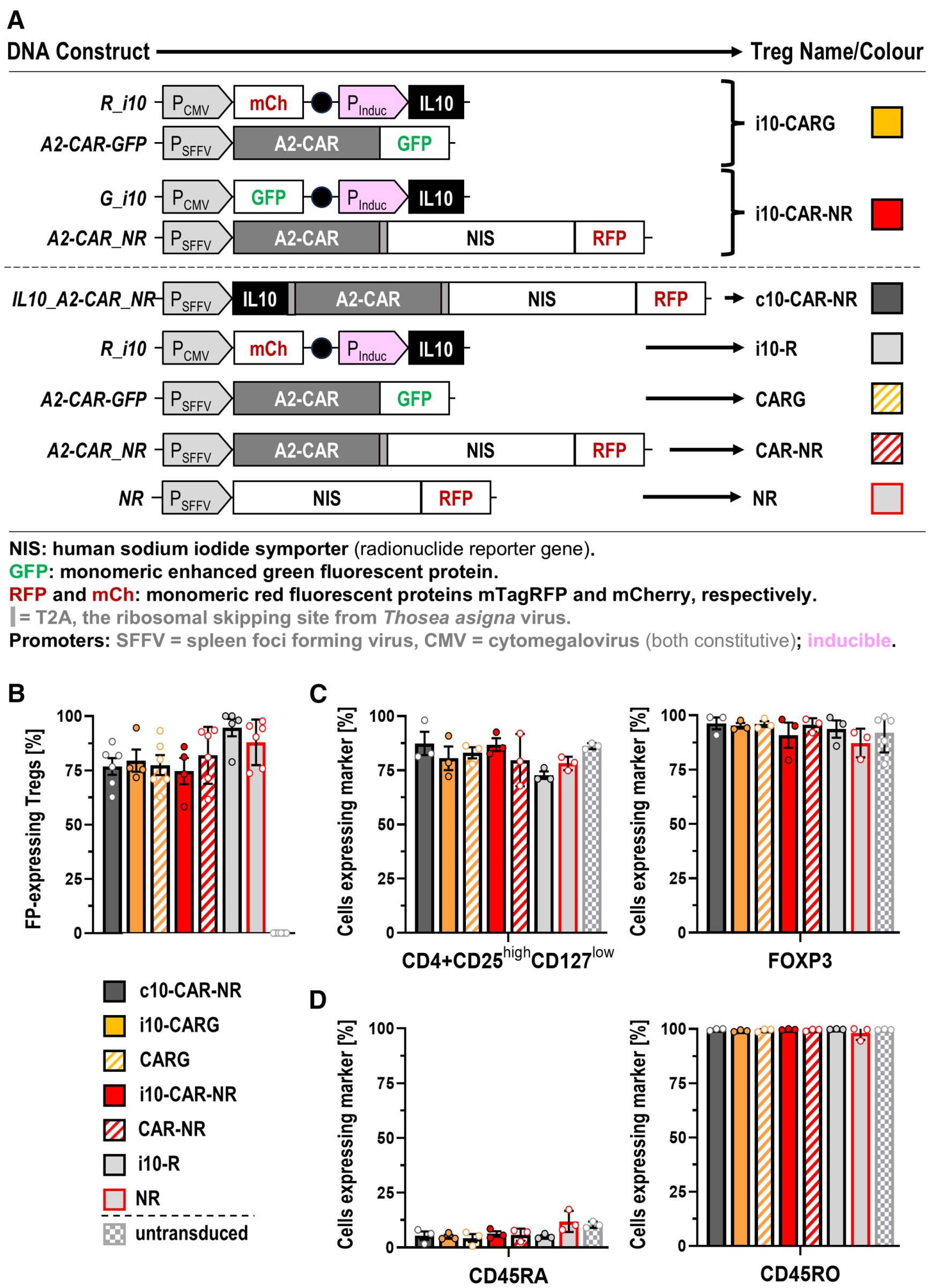

Figure 1. Engineering of A2-CAR Tregs with inducible IL-10 expression modules

(A) Scheme indicating which DNA constructs (left column) were used to generate the different Treg types (right column) including construct sketches and corresponding color codes for Tregs as used in this work. (B) Percentage of successfully engineered Tregs on day 20 of the protocol depicted in Figure S1; for the corresponding gating strategy, see representative plots in Figure S2. N = 3–6, and error bars represent SEM. (C) Engineered Tregs were analyzed for their phenotypes on day 20 including (left) evaluation of the retention of the intended $CD4^{+}CD25^{high}CD127^{low}$ marker combination and (right) FOXP3 expression. N = 3, and error bars represent SEM. (D) Analysis of (left) CD45RA and (right) CD45RO markers on day 20. N = 3, and error bars represent SEM. Statistical analyses in (B)–(D) were performed by one-way ANOVA with Tukey's multiple comparisons and α = 0.05 for all panels. No significant differences between indicated columns within a panel were found.

Notably, the presence of the A2-CAR together with the inducible IL-10 expression module did not impair expression of Treg markers or their homing molecules required for Treg migration and function.

Antigen-specific activation and cargo release of A2-CAR Tregs containing the inducible IL-10 expression module

To investigate whether i10-CARG Tregs recognized the HLA-A*02 antigen and were antigen-specifically activated, i10-CARG Tregs were co-cultured with a γ-irradiated HLA-A*02-expressing B cell lymphoblastoid cell line (B-LCL) for 24 h. Upon incubation with HLA-A*02 B-LCLs, we observed the upregulation of the activation marker CD69 in i10-CARG Tregs (mean 49.5%), which was comparable to CARG Tregs (mean 54.4%) and c10-CAR-NR Tregs (mean 50.6%), while no CD69 upregulation was observed in i10-R Tregs, which lack the A2-CAR ($p < 0.0001$ for all of i10-CARG, CARG, and c10-CAR-NR) or NR Tregs (Figure 2A). Incubation of A2-CAR-expressing Tregs with mismatch antigen HLA-A*01-expressing B-LCLs did not result in their activation above baseline levels (Figure 2A; for representative gating examples, see Figures S6A and S6B).

Beyond exploiting the established Treg activation marker CD69, we wanted to confirm whether activation was associated with nuclear translocation of the endogenous transcription factor NFAT, which was paramount for the downstream function of our antigen-specific IL-10 expression/secretion module. Using ImageStream technology, we analyzed Treg/B-LCL co-cultures for nuclear localization of NFAT in engineered Tregs. Nuclear NFAT localization was quantified by the mean similarity score (a measure of the overall similarity between images within the given stream). We observed similarity scores >3 for NFAT correlating with FOXP3, indicating strong nuclear translocation of NFAT in i10-CARG and CARG Tregs after co-culture with HLA-A*02 B-LCLs (Figure 2B). In contrast, in i10-R Tregs lacking the A2-CAR or under conditions in which i10-CARG and CARG Tregs were exposed to mismatched HLA-A*01 B-LCLs, we did not find nuclear translocation of NFAT (similarity scores ≤1; Figure 2B). As a reference point, the similarity scores obtained for polyclonal Tregs were similar to previously reported scoresa.[24] These data demonstrated that it was sufficient to exploit endogenous CD3ζ-to-NFAT signaling in this

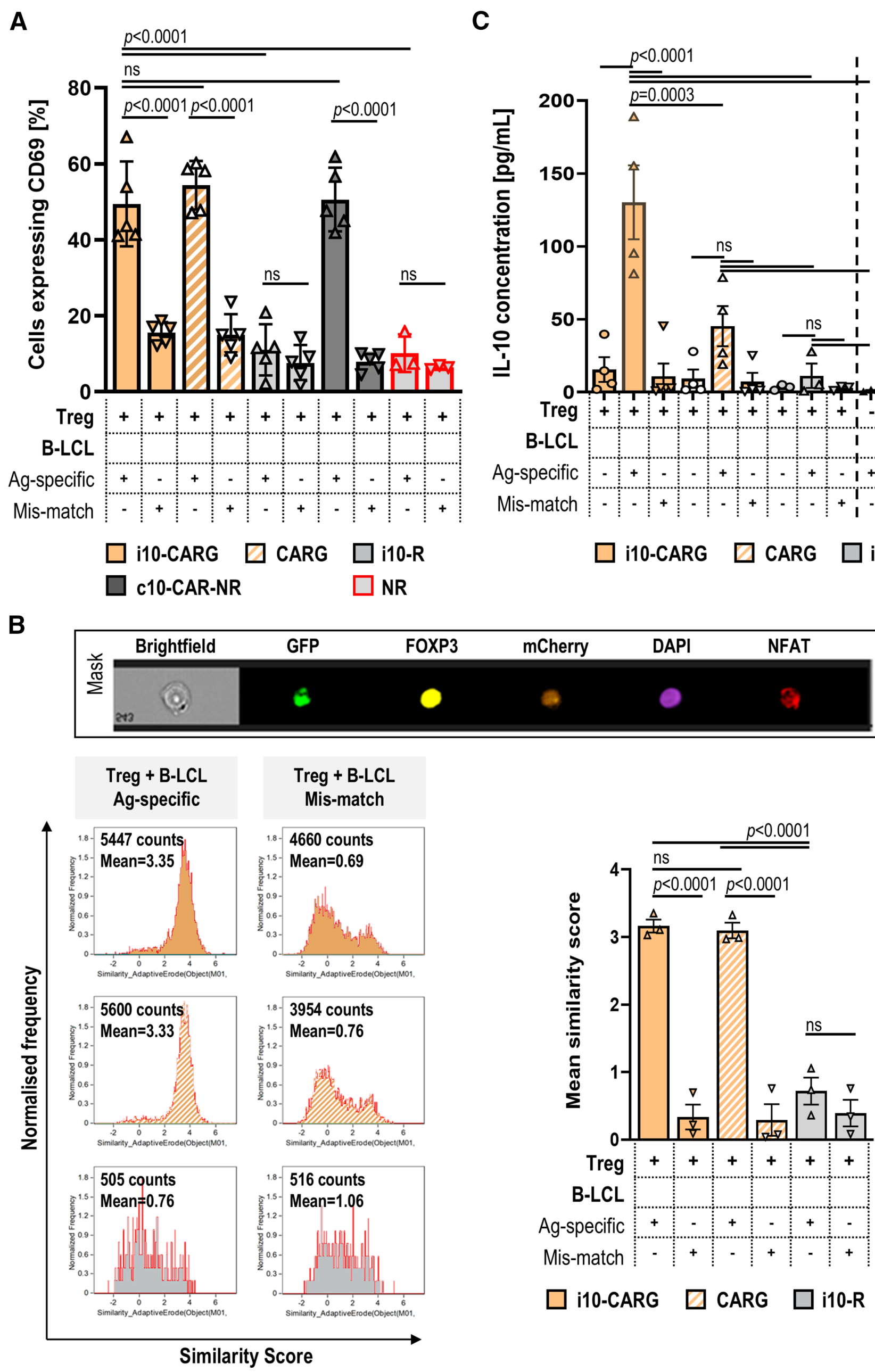

Figure 2. Antigen-specific activation, IL-10 secretion, and suppression of Tconv cell proliferation

Indicated Treg types were co-cultured for 18 h with B-LCL expressing either HLA-A*02 or the mismatch antigen HLA-A*01. (A) Cells were then analyzed for CD69 surface expression by flow cytometry (for gating, see Figures S7A and S7B). $N = 5$ engineered Treg batches ($N = 3$ for control NR Tregs) from different donors, with error bars representing SEM. (B) Co-cultures were analyzed by ImageStream for nuclear localization of the NFAT transcription factor. Top: exemplary ImageStream cell masks based on fluorescent proteins expressed as a consequence of Treg transduction (GFP, mCherry; cf. Figure 1), staining with dye-conjugated antibodies (FOXP3, NFAT), and a nuclear dye (DAPI). Markers allowed separation of Tregs from B-LCLs and determined nuclear NFAT localization. Bottom left: representative examples of the mean similarity score for nuclear NFAT localization obtained with indicated Treg types after co-culture with HLA-A*02 B-LCLs or HLA-A*01 B-LCLs. Bottom right: Cumulative mean similarity scores for nuclear NFAT localization. $N = 3$ independent experiments with Treg batches made from different donors; error bars represent SEM. (C) IL-10 concentration was determined in co-culture supernatants of indicated engineered Tregs with HLA-A*02 B-LCLs or mismatch HLA-A*01 B-LCLs as indicated. $N = 4$ Treg batches from different donors, with error bars representing SEM. Statistical analyses in (A)–(C) were performed by one-way ANOVA with Tukey's multiple comparison correction and $\alpha = 0.05$ for all panels. Exact p values for relevant comparisons are shown within relevant panels. ns, not significant. For comparison with constitutively IL-10 expressing c10-CAR-NR and NIS-RFP only expressing NR Tregs, see Figure S7.

context and suggested that the inducible IL-10 expression module was functional.

Importantly, in culture supernatants of i10-CARG Tregs that had been exposed to the HLA-A*02 B-LCLs, we found significantly higher levels of IL-10 compared to culture supernatants of CARG Tregs exposed to HLA-A*02 B-LCLs (cf. Figure 2A). This confirmed the function of the inducible IL-10 expression module. Cytokine analyses within the culture supernatants demonstrated that i10-CARG Treg co-cultures released ~2.5-fold more IL-10 compared to CARG Treg co-cultures ($p = 0.0003$; Figure 2C) and were significantly higher compared to supernatants from mismatch antigen HLA-A*01 co-cultures ($p < 0.0001$). As previously demonstrated, in c10-CAR-NR Tregs, the concentrations of IL-10 in cell supernatants were not modulated by HLA-A*02 antigen stimulation and were significantly higher than for i10-CARG and CARG Tregs when responding to antigen stimulation ($p < 0.0001$ and $p < 0.0001$, respectively; Figure S7). This was expected, given the IL-10 secretion in c10-CAR-NR Tregs was constitutive and not linked to CAR engagement and considering the high strength of the spleen focus-forming virus promoter driving CAR and IL-10 expression in these cells.

Antigen-specific suppressive capacity of A2-CAR Tregs containing the inducible IL-10 expression module

We next evaluated the capacity of A2-CAR Tregs containing the inducible IL-10 expression module to suppress conventional T

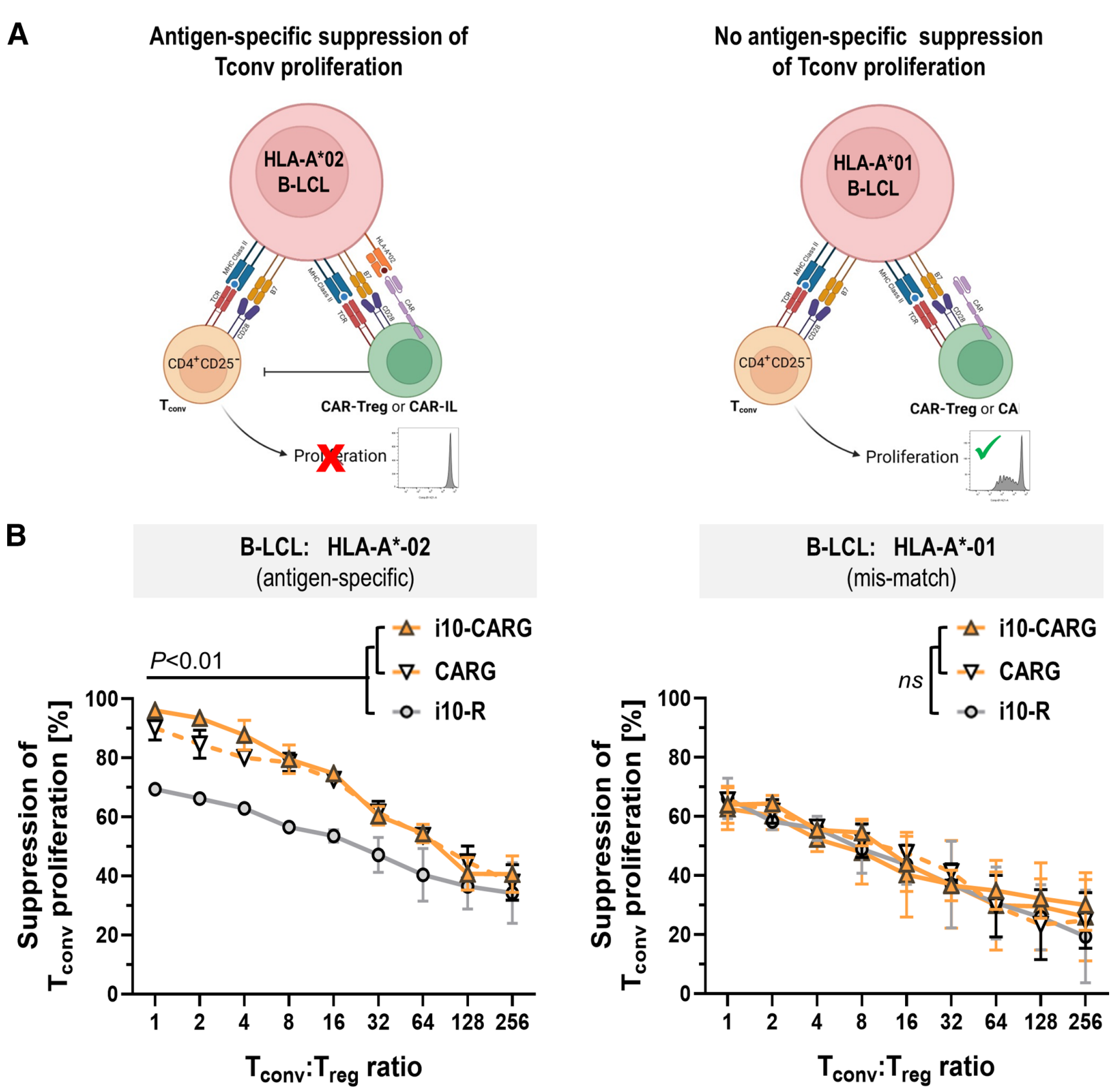

Figure 3. Antigen-specific suppression of Tconv cell proliferation

(A) Cartoon illustrating the setup of the antigen-dependent suppression assay to determine the suppressive capacity of Tregs to inhibit Tconv cell proliferation, with typically obtained representative proliferation results indicated in micrographs. (B) Suppression of Tconv cell proliferation (left) in the presence of HLA-A*02 B-LCLs or (right) in the presence of mismatch HLA-A*01 B-LCLs. $N = 3$ Treg batches from different donors with matching autologous Tconv; error bars represent SEM. Statistical analyses in (B) were performed by two-way ANOVA analyses for each panel comparing Treg means at each Tconv/Treg ratio including Tukey's multiple comparison correction ($\alpha = 0.05$). An overview of p values is shown atop figure panels; a detailed exact p value list for significant comparisons is shown in Table S6.

(Tconv) cell proliferation in an antigen-dependent manner. Treg preparations were co-cultured with autologous Cell Trace Violet (CTV)-labeled Tconv cells in the presence of HLA-A*02 B-LCLs or mismatch HLA-A*01 B-LCLs. The suppressive capacity of the Tregs was determined by flow-cytometric quantification of the inhibition of Tconv cell proliferation after 5 days of incubation (see scheme in Figure 3A). The inducible IL-10 module did not impair CAR-mediated antigen-specific suppression of autologous Tconv cell proliferation, while CAR presence resulted in significantly better suppression compared to Tregs without the A2-CAR (i10-R) for Tconv/Treg ratios up to 1:32 ($p < 0.01$). As expected, mismatch antigen exposure did not result in any differences between CAR Tregs and Tregs without CARs (i10-R) (Figure 3B; for representative gating examples, see Figures S6C and S6D). We then investigated corresponding co-culture supernatants for the presence of anti-inflammatory and pro-inflammatory cytokines. Notably, we found that i10-CARG Tregs secreted higher amounts of the anti-inflammatory cytokine IL-4 and lower amounts of the pro-inflammatory cytokines tumor necrosis factor α (TNF-α), interferon-γ (IFN-γ), and IL-17A compared to CARG Tregs and i10-R Tregs (Figure S8).

Antigen-specific modulation of cell-surface markers of antigen-presenting monocytes

Beyond B-LCLs, we also investigated monocytes as antigen-presenting cells, as they are the major cellular component of inflamed tissues. Previously, we had found that expanded Tregs were able to drive monocytes toward an alternatively activated state[25] resulting in a reduced production of pro-inflammatory cytokines and upregulation of "M2" macrophage-specific markers compared to freshly isolated Tregs. Here, we investigated whether our lentivirally engineered CAR Tregs and, in particular, those with the ability to secrete IL-10 cargo (either in an antigen-specific or constitutive manner) could also polarize monocytes toward an alternatively activated state. Therefore, we isolated human $CD14^+$ monocytes from HLA-A*02-positive peripheral blood mononuclear cell (PBMC) donors and used them to establish co-cultures with either i10-CARG, CARG, c10-CAR-NR, or untransduced Tregs (monocyte/Treg ratio was 2:1; Figure S9A). As control, we cultured monocytes in the absence of Tregs. We found that all CAR Tregs/Tregs upregulated HLA-DR, CD16, and CD40 as well as CD163, B7-H4, and CD206 compared to monocytes that had not been in co-culture with Tregs (Figures S9B and S9C). Notably, the presence of IL-10 secretion, i.e., in co-cultures with i10-CARG-secreting IL-10 under antigen stimulation as well as in co-cultures with c10-CAR-NR that constitutively secreted IL-10, we found lesser upregulation of HLA-DR, CD16, and CD40 than in co-cultures with Treg types without IL-10 secretion beyond their endogenous capacities (Figure S9B). These *in vitro* data demonstrated a regulatory effect of expanded engineered Tregs on antigen-matched monocytes.

A2-CAR Tregs with the inducible IL-10 expression module outperform A2-CAR Tregs with constitutive IL-10 overexpression in a xeno-GvHD model

A2-CAR Tregs have previously been shown to outperform polyclonal Tregs in various preclinical applications.[11–13] Here, we set out to evaluate the performance of i10-CARG Tregs in a xenogeneic graft-versus-host disease (xeno-GvHD) model.[26] In this model, immunodeficient NSG mice received HLA-A*02 PBMCs

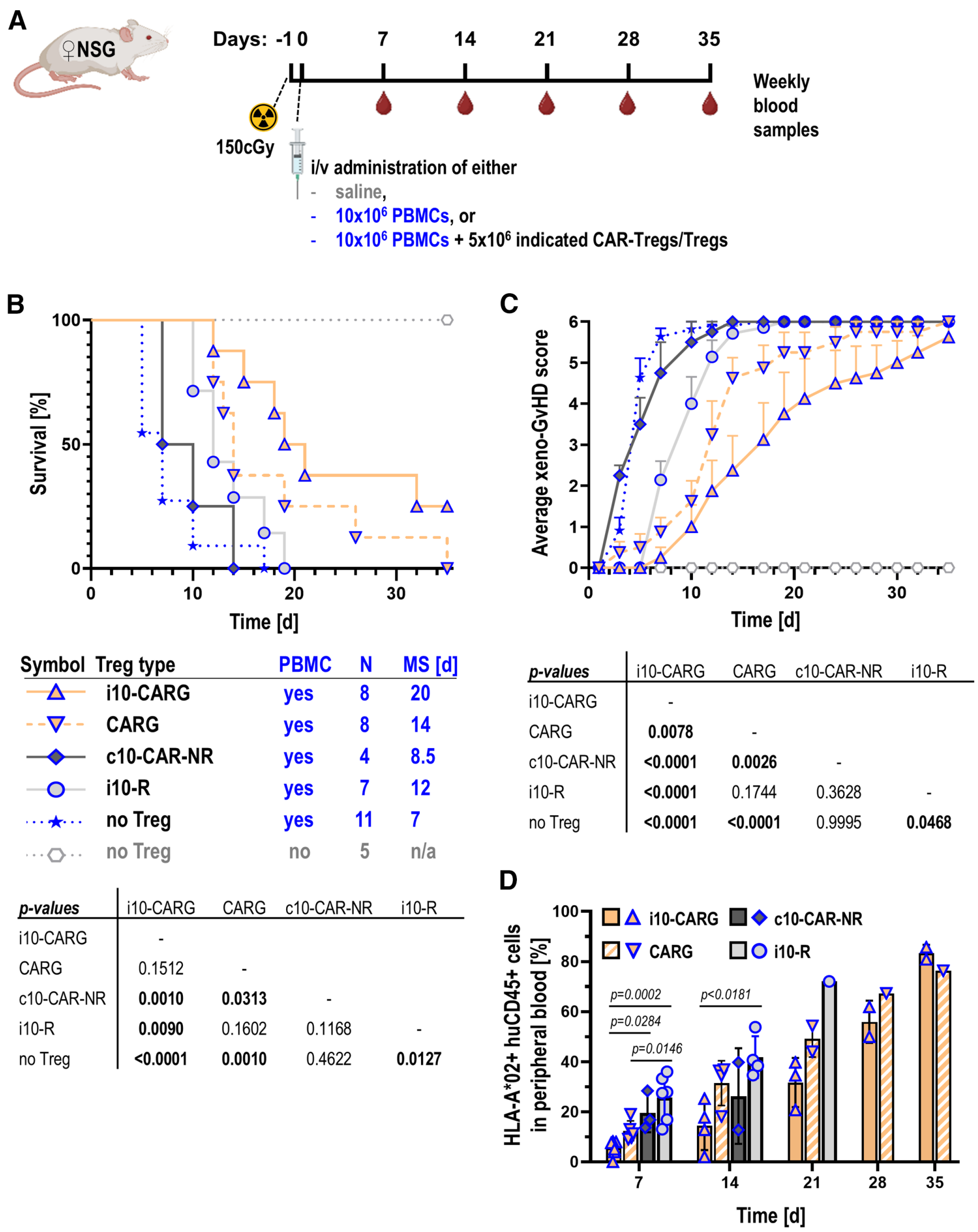

Symbol	Treg type	PBMC	N	MS [d]
△	i10-CARG	yes	8	20
▽	CARG	yes	8	14
◆	c10-CAR-NR	yes	4	8.5
○	i10-R	yes	7	12
★	no Treg	yes	11	7
○	no Treg	no	5	n/a

p-values	i10-CARG	CARG	c10-CAR-NR	i10-R
i10-CARG	-			
CARG	0.1512	-		
c10-CAR-NR	**0.0010**	**0.0313**	-	
i10-R	**0.0090**	0.1602	0.1168	-
no Treg	**<0.0001**	**0.0010**	0.4622	**0.0127**

p-values	i10-CARG	CARG	c10-CAR-NR	i10-R
i10-CARG	-			
CARG	**0.0078**	-		
c10-CAR-NR	**<0.0001**	**0.0026**	-	
i10-R	**<0.0001**	0.1744	0.3628	-
no Treg	**<0.0001**	**<0.0001**	0.9995	**0.0468**

Figure 4. Evaluation of different engineered Treg types in a xeno-GvHD model

(A) Scheme of the xeno-GvHD model. (B) Survival curves for NSG mice that received 10 × 10^6 HLA-A*02-positive PBMCs with or without indicated Treg types (5 × 10^6 each). "Survival" is defined as GvHD scores being below the threshold triggering the humane endpoint and therewith animal sacrifice. Pooled survival rates from two independent experiments wherein Tregs were engineered from two different human donors (PBMCs also from two human donors but not the same donor as Tregs). Numbers and median survival (MS) per cohort are listed below the graph. Statistical analysis by Mantel-Cox analysis; for *p* values of individual comparisons, see table at the bottom. (C) Progression of xeno-GvHD as per the regulator-approved scoring system for cohorts shown in (B). Recorded curves could not be well fitted with the same equation and hence were statistically evaluated by area-under-curve (AUC) analysis followed by one-way ANOVA of AUC means and standard errors including Tukey's multiple comparison correction. Day 0 in (B) and (C) indicates the day of cell administration. (D) Mean percentage of HLA-A*02-positive cells in cohorts treated as indicated. At the start, *N* = 6 for this analysis for i10-CARG, CARG, and i10-R cohorts and *N* = 4 for the c10-CAR-NR cohort; *N* decreased over time as animal numbers decreased (cf. B). Means and SEM are shown. *p* values listed are from significant comparisons and stem from one-way ANOVA including Tukey's multiple comparison correction performed at each time point.

followed by administration of indicated Tregs. The PBMCs were not from the same human donor as the engineered Tregs. PBMC engraftment and proliferation induced GvHD, which was monitored through GvHD scores (see materials and methods) up to the time points when animals reached the humane experimental endpoint; the median survival was 7 days from PBMC administration (Figure 4B; no Tregs + PBMCs, positive control). Control animals that were not given PBMCs did not develop GvHD (neither Tregs nor PBMCs, negative control). When polyclonal Tregs were administered after PBMCs, the onset of GvHD was delayed in line with previous observations and with a median survival of 12 days for this cohort (Figure 4B; i10-R Tregs, $p = 0.0127$ compared to the PBMC-only cohort). CARG Tregs expressing the CAR targeting HLA-A*02 fared even better, with a median survival of 14 days. Notably, animals that had received i10-CARG Tregs showed an additional improvement in survival, with a median survival of 20 days compared to polyclonal Tregs (Figure 4B; $p = 0.0090$). In contrast, animals that had received c10-CAR-NR Tregs, which express IL-10 constitutively and at higher levels than i10-CARG Tregs (cf. Figure 2C), presented with a median survival of 8.5 days, which was significantly less than that of i10-CARG Tregs ($p = 0.0010$). The constitutive IL-10 expression resulted in the loss of the beneficial effect of CAR expression ($p = 0.0313$ compared to CARG Tregs) and rendered these c10-CARG-NR Tregs ineffective for the protection against GvHD ($p = 0.4622$ compared to positive control for GvHD). Corresponding average xeno-GvHD score data are shown in Figure 4C and corroborate Figure 4B (for *p* values, see figure panels). From weekly blood samples, we determined the percentage of engrafted HLA-A*02-positive human $CD45^+$ cells. We also found that animals that had received i10-CARG and CARG Tregs showed initially lower PBMC engraftment compared to other conditions (Figures 4D and S10), suggesting that A2-CAR Tregs were either reducing PBMC engraftment or delaying PBMC expansion and thereby delaying xeno-GvHD symptom development.

Upon reaching humane experimental endpoints, animals were sacrificed, and lungs, liver, intestines, and femur for bone marrow were formalin-fixed, paraffin-embedded, sectioned, and stained

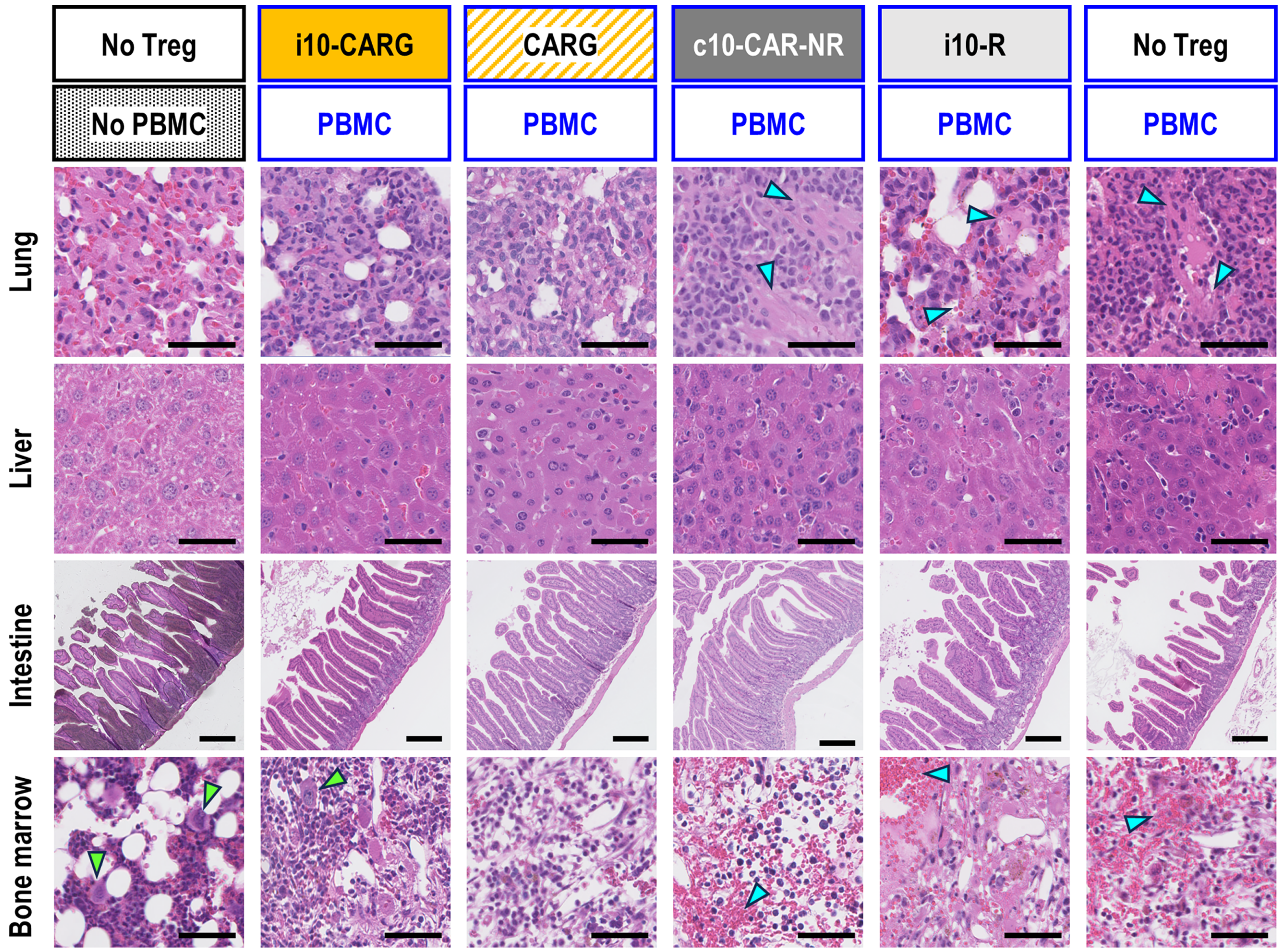

Figure 5. Lung, liver, intestine, and bone marrow tissue morphology in the xeno-GvHD model

Indicated organs from animals in Figure 4 were sectioned, stained with H&E, and investigated for signs of immune cell infiltration and tissue damage by microscopy. Micrographs shown are representative for each cohort and taken from the same animal per cohort; all shown animals were culled in week 3 of the experiment (except for c10-CAR-NR animals, which were culled in week 2; cf. Figure 4B). (Left column) Animals treated without PBMC/Tregs developed no signs of GvHD and served as healthy controls, while animals treated with only PBMCs served as GvHD positive controls, and the other columns are color coded as in previous figures. Scale bars, 50 μm. Cyan arrows indicate multifocal inflammation necrosis, while green arrows indicate the protection of megakaryocytes in the bone marrow.

with hematoxylin and eosin (H&E) for xeno-GvHD-related pathological lesion identification. Blood for serum preparation and IL-10 and IFN-γ determination was also collected, but at this sampling point only minute cytokine amounts were detected in sera that were mostly very close to or below the limit of detection (Figure S11). Due to animal license restrictions, serial sampling at volumes sufficient for repeat cytokine determination was not possible, representing a technical limitation. In tissues, we observed widespread signs of multifocal inflammation necrosis (Figure 5, cyan arrows) and blood stasis with focal hemorrhage in lung tissues and heavy multifocal inflammation in liver sections. Notably, we also found damage to mucous epithelial cells, lymphoid infiltration, destruction of crypts, and formation of extensive necrotic-ulcerous defects in intestines, while we also detected hemorrhage and hypocellular marrow (Figure 5). Across all tissues, we found less immune cell infiltration and tissue destruction in animals that had received i10-CARG Tregs and CARG Tregs compared to counterparts that had received c10-CAR-NR Tregs, i10-R Tregs, or PBMCs alone (Figure 5). Remarkably, even though the animals were sacrificed later due to a delayed GvHD, megakaryocytes in the bone marrow were less pronounced in animals that had received i10-CARG Tregs and CARG Tregs (Figure 5, green arrows). Moreover, we stained adjacent tissue sections for human CD3 or human FOXP3 to identify the existence of administered total T cells and administered Tregs, respectively (Figure 6). In animal tissues from i10-R Tregs lacking the A2-CAR, we found hardly any Tregs revealed by FOXP3 foci in both the liver and intestine, corroborating the data presented herein. Conversely, substantial lesion-induced non-specific FOXP3 signals were observed in the hemorrhagic regions of the lung and bone marrow in these animals, indicating that non-CAR engineered Tregs are ineffective in delaying the progression of xeno-GvHD. Notably, we generally were able to detect administered engineered Tregs by this approach and found higher Treg numbers in tissues when i10-CARG Tregs were co-administered with PBMCs as compared to tissues from animals that received other Treg types.

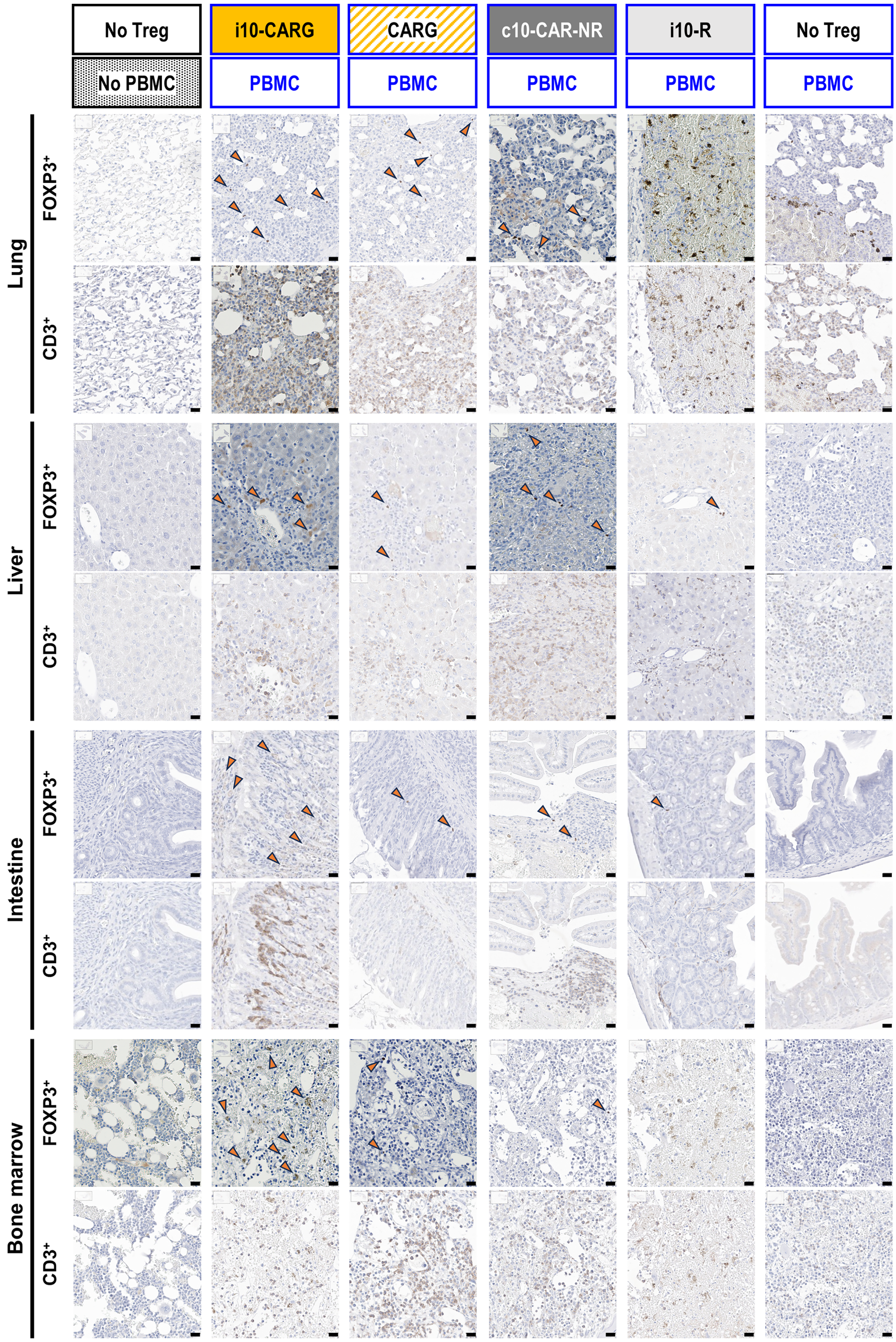

(legend on next page)

DISCUSSION

Building on our previous work demonstrating Treg and CAR-Treg engineering to enable *in vivo* imaging[27] and constitutive cargo release,[21] we report here, for the first time, that CAR-Tregs can also be engineered to express cargo in a manner directly linked to CAR-antigen engagement. To achieve this, we exploited the NFAT transcription factor machinery endogenous to Tregs. Previously, the NFAT machinery of effector T cells has been utilized to *in vivo* report effector T cell activation by imaging[28] and to express IL-12 or IL-18 in response to CAR activation[14,15]; however, this has so far not been demonstrated in Tregs in this form and avoids manipulation of autoinhibitory molecules.[29] Although we have focused here on IL-10 as a proof-of-principle cargo, the same NFAT-driven delivery platform could be readily adapted to other immunomodulators such as transforming growth factor β, programmed death ligand-1, or the ectonucleotidase CD39, to establish a versatile pipeline of antigen-triggered Treg therapies. Notably, this approach differs from recently reported synthetic receptor technologies including SynNotch[30] and SNIPR,[31] which are independent of endogenous NFAT signaling and exploit a synthetic signaling cascade that also must be introduced into the T cells through gene-delivery approaches. SNIPR in particular offers effective tunability, as has been demonstrated in effector T cells; however, neither SynNotch nor SNIPR or similar more complex circuitry have so far been applied to CAR-Tregs.

To date, effector CAR-T cell therapies are commercially available for patients suffering from certain relapsed/refractory lymphomas or multiple myeloma,[32] with reports of very promising results in lupus erythematosus treatment,[33,34] while CAR-Treg technology has reached clinical trial stages (e.g., NCT04817774 and NCT05234190). Notably, all current commercialized products employ second-generation CARs without any additional cargoes, while more complex and larger synthetic constructs are emerging. Current commercial products utilize viral gene-transfer technologies despite their limitations on transgene size.[35,36] While we have used double transduction of two smaller constructs followed by FACS-based purification in this proof-of-principle study, notably without negative impact on expansion capability (Figure S3A), it is possible to combine our components into one large vector and remain within viral packaging limits. For example, a single vector including the HLA-A*02 CAR and the NFAT activation-dependent IL-10 secretion module would amount to a provirus size smaller than 6 kb, thereby remaining in a favorable region below virus packaging limits. Furthermore, a single vector approach would limit potential safety concerns, i.e., the risk of insertional mutagenesis as measured by an increased vector copy number. Notably, we have previously generated CAR Tregs with additional cargo modules with transgene and provirus sizes up to 4.85 kb and 8.67 kb, respectively.[21] Alternatively, gene-editing technologies have been applied to engineer human Tregs.[37–39] While we have recently shown gene editing of medium-sized constructs in human stem cells (2.7 kb),[40] such conventional approaches generally also present with transgene size limitations.[41] Importantly, newer gene-editing technology has shown promise to overcome these limitations (with reported examples of 8 kb and 10 kb transferred).[42,43] It is therefore conceivable that, at variance to larger-sized synthetic signaling circuitry modules, our relatively small-sized secretion module (~1.1 kb) exploiting the endogenous NFAT signaling machinery of Tregs may be introduced into CAR Tregs destined for clinical translation even prior to the maturation of efficient technologies enabling gene transfers ~10 kb.

In vitro characterization of our CAR Tregs demonstrated expected phenotypes (Figures 1 and S2), effector T cell suppression (Figure 3), expansion capabilities (Figure S3A), and expression of homing markers (Figure S5) as well as the retention of imaging reporter function (Figure S3B). We further demonstrated CAR-antigen-specific activation of these Tregs including upregulation of CD69 expression, nuclear translocation of NFAT, and corresponding NFAT-dependent expression and secretion of the engineered IL-10 transgene (Figure 2). Moreover, we demonstrated monocyte modulation properties (Figure S9). The patterns observed suggested that IL-10 exerted a dampening effect on monocyte activation, maintaining lower expression levels of key co-stimulatory and Fc-receptor molecules during cell-cell interaction. Consequently, these data indicated that IL-10 produced by CAR T cells can modulate the antigen-presenting phenotype of monocytes, promoting a more tolerogenic or less inflammatory profile, whereas in the absence of IL-10 (as in the CARG-Treg condition), monocytes retained a more activated and immunostimulatory phenotype comparable to untransduced Tregs (Figure S9). Such modulation of monocyte activation markers is consistent with IL-10's known capacity to suppress nuclear factor κB-dependent transcription[44] of HLA-DR and CD40 and to down-regulate Fcγ receptor expression, thereby limiting antigen presentation and pro-inflammatory cytokine release.

Subsequently, we employed a xeno-GvHD mouse model, which is a well-established approach to assess the preclinical efficacy of Tregs or CAR Tregs *in vivo*.[12,26,45,46] Here, adoptive transfer of various Treg preparations into irradiated NSG recipient mice was performed but with HLA-A*02 PBMCs administered through separate injections. This ensured that the various administered Treg types received CAR activation *in vivo* and that the effects observed were not biased due to activation already taking place prior to *in vivo* administration at the stage of mixing Tregs and PBMCs *in vitro* when preparing the injections. The results were obtained through

Figure 6. Staining for CD3⁺ and FOXP3⁺ cells in lung, liver, intestine, and bone marrow tissues in the xeno-GvHD model

Selected organs from animals in Figure 4 were sectioned and adjacent sections stained with antibodies against either human CD3 or human FOXP3 (brown stain) and a nuclear counterstain (blue stain) before being imaged by bright-field microscopy. Micrographs shown are representative for each cohort and taken from the same animals per cohort, all of which were culled in week 3 of the experiment (except for c10-CAR-NR animals, which were culled in week 2; cf. Figure 4B). Scale bars, 20 μm. Arrows indicate FOXP3-positive cells indicative of experimentally administered therapeutic Tregs as indicated in each column.

humane endpoint assessment in line with ethical regulations in the United Kingdom, including weight-loss determination and GvHD scoring (Figure 4). Results from polyclonal and A2-CAR-Treg mice were comparable to data from previous studies.[12,45] *Ex vivo* histology confirmed our results (Figures 5 and 6); notably, we found more adoptively transferred human Tregs across indicated tissues when we administered i10-CARG or CARG Tregs as compared to when we used polyclonal Tregs (i10-R). This was in line with the delayed development of GvHD and extended survival, demonstrating that the adoptively transferred therapeutic cells not only trafficked to the target sites but were correlated with their positive effects.

We previously had developed CAR Tregs that constitutively expressed IL-10. While they showed enhanced suppressive capacity compared to polyclonal Tregs and CAR Tregs without the constitutive IL-10 secretion module, we also found that these cells produced approximately 50-fold more IL-10 than activated Tregs/CAR Tregs lacking the constitutive IL-10 module.[21] Due to the pleiotropic effects of IL-10, systemic and/or off-target secretion of IL-10 at high levels may present safety and toxicity concerns, especially as it has previously been shown that high levels of systemically administered IL-10 exacerbated xeno-GvHD by promoting significant expansion of human effector T cells; the effects were blocked by an antibody against IL-10.[47] Consequently, we included this previously generated constitutively IL-10-releasing CAR construct (c10-CAR-NR) for comparison. We observed that c10-CAR-NR Tregs had negligible effects in our xeno-GvHD model (Figures 4, 5, and 6). Future work involving molecular analyses across different tissues and aimed at revealing the differential effects caused by either antigen-specific or constitutive expression of IL-10 will be required to identify the precise mechanisms governing the observed effects.

First, IL-10 dose and kinetics will require future attention. IL-10 expression capacity of therapeutic cells and tissue-delivered concentrations are intrinsically linked; hence, more research is needed to evaluate beneficial ranges for agent secretion in armored CAR Tregs. Our data may also strengthen the hypothesis that CAR-engagement-dependent cargo release should remain at near-endogenous levels. This is particularly important, as higher IL-10 serum concentrations may be deleterious in xenogeneic settings. The latter requires serial blood sampling and analysis, but this could not be undertaken in this study due to sampling restrictions dictated by our animal license, thereby representing a limitation of this work. Beyond GvHD, for example in the context of organ transplantation, this also suggests addressing possible "spill-over" effects from target tissues into circulation and, thus, evaluation of potential systemic cargo effects, placing emphasis on the importance of suitable preclinical models.

Second, it will be crucial to evaluate the location and consequences of on-target antigen-specific release of immunomodulatory agents. This will help identify whether and with what kinetics CAR-Treg-released cargo may become detectable and exert effects in target tissues and beyond. By situating the IL-10 cassette under an NFAT endogenous promoter, cytokine production becomes contingent on active CAR signaling as was demonstrated in this work. The differential in IL-10 production with antigen-specific stimulation should sharply limit systemic "spill-over" while concentrating immunoregulation at the graft interface. We cannot rule out that new tunable but more complex synthetic signaling circuitries could achieve similar results in the future. However, we believe that our approach is simple to implement and showed that our strategy did not negatively impact other critical attributes of the engineered Tregs (e.g., phenotype, expansion capability, and function; cf. Figures 1, 3, S3, and S5). Cargo release is intrinsically linked to cell therapy presence and hence its evaluation, for example through reporter gene-afforded imaging, will be highly beneficial in this context (see also below).

Finally, it remains critical to carefully consider precise model parameters and settings. While we performed *in vivo* assessment of various human engineered Treg types in a xeno-GvHD model in immunodeficient mice, it is possible that Treg performance differs when employed in other settings or disease models, for example, transplantation or autoimmunity. Moreover, in the GvHD setting it is possible that $CD8^+$ effector T cells are involved in driving xenogeneic disease severity. We previously showed the important role of cells with $CD8^+$ T-effector memory phenotype in xeno-GvHD models.[48] It is noteworthy that $CD8^+$ T cell activation was previously reported when they were exposed to higher-than-endogenous IL-10 levels. For example, in a murine model of autoimmune cholangitis, endogenous IL-10 was found to maintain immune tolerance, while systemic IL-10 expression yielding serum concentrations in the hundreds of pg/mL resulted in the exacerbation of the model condition.[49] Another example was reported in oncology, where systemically expressed IL-10 at serum concentrations similar to those of the aforementioned autoimmune study resulted in $CD8^+$ T-cell-mediated tumor control.[50] It is therefore plausible that xeno-GvHD severity may be elevated in the presence of non-targeted IL-10 exposure above certain levels, and further investigations in this context will be warranted.

We also would like to point out that our data indicate limitations of various *in vitro* assays and illustrate the importance of suitable preclinical experimentation. Beyond phenotyping, activation, and cytokine release measurements, we utilized co-culture-based assays aimed at revealing suppression of effector cell proliferation (Figure 3A) and monocyte modulation properties (Figure S9), respectively. For example, judging from our *in vitro* suppression data, one would certainly not expect a detrimental effect of constitutive IL-10 expression in CAR Tregs (c10-CAR-NR) and, instead, similar behavior compared to our inducible IL-10 release approach (i10-CARG). However, our data from the xeno-GvHD model revealed different results indicating that none of our *in vitro* assays adequately predicted the *in vivo* behavior observed in this model.

In this work, we demonstrated antigen-specific cargo release via CAR-antigen engagement for a defined matched pair, i.e., the

HLA-A*02 antigen and our anti-HLA-A*02 targeted CAR employed in a murine xenograft setting.[11] While this approach simplified the interpretation of our data, it also presented a limitation, namely, limited antigen breadth. We had originally chosen to utilize HLA-A*02 because of its high frequency in most human populations.[51] The latter offered a strong rationale to develop a CAR to target it. It is important to note, however, that it will require future work employing different antigen-CAR pairs to demonstrate the generalizability of our approach reported here. This will not only be important for academic reasons but also for future development of CAR-based therapeutics for autoimmune conditions to make them available for an even wider range of potential patients.

In adoptive cell therapy, long-term persistence represents another important factor. Its determination in animal models is generally limited by the duration the model can be used, which is not least limited by ethical requirements and humane endpoints. Moreover, the aspect of how complete the animal host's immune system is affects how relevant findings are for subsequent clinical translation. The xeno-GvHD model we used here was limited to ~5 weeks in the longest cases with i10-CARG cells being detectable in relevant tissues (Figure 6). While methodology to sensitively determine persistence in circulation is available, e.g., quantitative PCR, corresponding methodology for analyses within tissues is much more challenging. The latter require either multiple sampling and histology or serial non-invasive *in vivo* whole-body imaging. This imaging approach has been exploited for adoptive immunotherapy tracking over the last decade, whereby non-immunogenic reporter genes are particularly useful on the preclinical level and are envisaged for clinical translation. We have previously exploited this avenue to quantify Treg homing to transplants[27] and demonstrated that required reporter gene cassettes can be included in CAR Tregs.[21] Hence, we also evaluated the generation of *in vivo* traceable i10-CAR-NR Tregs as part of this work (Figures 1, S1–S3, and S5). Alongside long-term persistence, phenotypic stability is also crucial in the context of these cell therapies; however, it is even more challenging to access, with phenotypic and Treg-specific demethylated region (TSDR) methylation-state analyses being warranted as part of the future clinical translation pathway.

In conclusion, we demonstrated herein that human CAR Tregs can be engineered to conditionally release the immunosuppressive cytokine IL-10 upon CAR-specific engagement, characterized these cells *in vitro*, and showed that these CAR Tregs exerted beneficial effects in delaying deterioration of disease symptoms in a preclinical xeno-GvHD mouse model. We believe that this study provides proof of concept for a new class of Treg-based engineered cell therapies and warrants their evaluation in conditions associated with a breakdown of immune tolerance.

MATERIALS AND METHODS

Standard reagents and plasticware were from Merck, Sarstedt, Thermo Fisher, TPP, or VWR unless otherwise specified.

Human Treg isolation and expansion

Human blood was obtained with ethical approval (Institutional Review Board ref. #09/H0707/86) from the National Blood Service (NHS Blood and Transplantation, Tooting, London, UK). $CD4^+CD25^+$ Tregs and $CD4^+CD25^-$ effector T cells were isolated from leukocyte-enriched leukapheresis blood cones, with ethical approval (see section below). $CD4^+$ T cells were isolated from total PBMCs by negative selection using Rosette-Sep (STEMCELL Technologies, UK) and enriched for $CD25^+$ T cells using CD25 Microbeads II (Miltenyi Biotec, UK). Collected $CD4^+CD25^-$ Tconv cells were preserved by freezing in liquid nitrogen until use. $CD4^+CD25^+$ Tregs were cultured at 1×10^6 cells/mL in X-Vivo 15 (Lonza, Switzerland) and activated with anti-CD3/CD28 Dynabeads (1:1 bead/cell ratio; Thermo Fisher, UK). Growth media were supplemented with 1,000 U/mL IL-2 (R&D Systems, MN, USA) and 100 nM rapamycin (LC-Laboratories, MA, USA) and replaced every 2 days. On days of harvest, Dynabeads were removed by magnetic force before any subsequent analyses.

DNA constructs

DNA plasmids carrying the constructs as indicated in Figure 1A (left column) were used to generate the various Treg types in this study. The transgene plasmids "IL10_A2-CAR_NR," "A2-CAR_NR," and "NR" were previously described[21] as well as "A2-CAR-GFP."[11] For "R_i10," the commercial pLJM1 mCherry plasmid was digested with the restriction endonucleases *Sac*II and *Nsi*I, thereby removing its PGK promoter and puromycin resistance and serving as the vector backbone. A DNA sequence named "NF10" was synthesized (Genewiz, UK), which consisted of the following elements in order: a *Hpa*I restriction site, six NFAT binding sites, a minimal IL-2 promoter, a *Bam*HI restriction site including a Kozak element, and DNA encoding human IL-10, all flanked by restriction sites for *Sac*II and *Nsi*I, respectively. This "NF10" was subcloned in the vector backbone giving rise to "R_i10." For "G_i10," EGFP was amplified by PCR from "A2-CAR-GFP" with flanking *Nhe*I and *Bsp*EI restriction sites and subcloned into the "R_i10" replacing mCherry. All constructs were confirmed by Sanger sequencing (Genewiz).

Generation of engineered Tregs

Lentiviral particles were produced and batch titers determined as previously described.[52] Virus batches were frozen and stored at −80°C until use. Treg transduction with lentiviruses was performed in 24-well sterile plates (Corning, UK) precoated with 4.8 μg of RetroNectin (Takara, UK; in 0.5 mL PBS per well for 2 h at room temperature). Titered lentiviral particles were thawed and added to each well before 0.5×10^6 Tregs (MOI ~ 5) were added per well with X-Vivo 15 complete medium containing 1,000 IU/mL IL-2 added to a final volume of 250 μL. Plates were spinoculated (600 × *g*, 1 h at 42°C) and incubated at 37°C in humidified air containing 5% (v/v) CO_2. On the next day, 750 μL of X-Vivo 15 complete medium containing 1,000 IU/mL IL-2 and 100 nM rapamycin was added, and cells were expanded for a week (representing day 10 of the culture). Suitable Treg aliquots were collected, Dynabeads were removed by magnetic force separation, and Tregs were resuspended

at 20 × 10^6 cells/mL in PBS and labeled with 1 μg/L DAPI (Thermo Fisher Scientific, UK). Cells were incubated for 10 min at 37°C before being washed and resuspended in 200 μL of sterile PBS. Cells were strained through a 70-μm strainer into sterile FACS tubes (Corning). Cells were then sorted into sterile tubes containing X-Vivo 15 medium based on viability discrimination and fluorescent protein reporter expression using a BD FACSAria II (BD Biosciences, UK) equipped with an 85-μm nozzle (for optical flow cytometer settings, see Table S1). Notably, research-grade lentiviral transduction generates polyclonal cell products with variable integration sites and copy numbers.[53,54] Copy-number determination, e.g., by digital PCR, would be required for every individual batch to be informative and is not customary in discovery studies; hence, we also omitted it. Fluorescent protein expression was used to measure transduction (Figure S2) and as a surrogate informed on CAR expression, either due to direct CAR-GFP fusions (in i10-CARG, CARG) or due to 2A technology[55]-afforded near equimolar expression from multi-construct cassettes (in i10-CAR-NR, c10-CAR-NR, and CAR-NR). However, fluorescent protein expression is confounded by positional effects and hence is not a reliable proxy for copy-number determination.

Treg phenotype analysis

Cell staining was performed using 2.5 × 10^5 cells in 100 μL of PBS containing 2% (v/v) FBS and 0.5 mM EDTA ("flow buffer"; Thermo Fisher Scientific). Dead cells were identified using a 1:1,000 dilution of the LIVE/DEAD Fixable Near IR stain kit (Life Technologies, UK) in PBS for 30 min at 4°C. Fluorescently conjugated antibodies were added to the cells at concentrations of 2–5 μg/mL for specific antigens, according to the manufacturer's instructions for use (for antibody details, see Table S2). The cells were then incubated at 4°C for 20 min.

For intracellular FOXP3 staining, cells were fixed and permeabilized with the FOXP3/Transcription Factor Fixation/Permeabilization kit (Invitrogen, Thermo Fisher Scientific) for 1 h at 4°C prior to antibody staining. Cells were washed twice with 1:10-diluted permeabilization buffer for 10 min, resuspended in 100 μL of permeabilization buffer containing antibodies, and incubated for a further 45 min at 4°C.

After staining, all cells were washed twice before being resuspended in 300 μL of permeabilization buffer and analyzed on an LSR Fortessa flow cytometer (BD Biosciences, UK).

NIS radionuclide reporter function

3 × 10^6 indicated CAR Treg cells were transferred into Eppendorf tubes, washed with ice-cold Hank's balanced salt solution (HBSS; Thermo Fisher), and resuspended in 1 mL of growth medium. $^{99m}TcO_4^-$ was generator-eluted as a sodium salt solution and used within 6 h of elution (supplied by local King's Health Partners' Radiopharmacy; ^{99m}Tc half-life is 6.0 h). A 50-kBq-generator-eluted sodium $^{99m}TcO_4^-$ solution was added to cells, which were then incubated for 30 min at 37°C. Subsequently, cells were pelleted, supernatants collected, and cells washed twice with 1 mL of HBSS before being resuspended in growth medium for γ-counting (1282 Compugamma, LKB-Wallac). If the competitive substrate perchlorate (12.5 μM) was used to determine radiotracer uptake specificity, cells were preincubated with perchlorate for 30 min in growth medium, and perchlorate was present at the same concentration during the radiotracer uptake assay. Radiotracer uptake was calculated according to Equation 1, where Cpm represents decay-corrected radioactivity counts per minute.

$$\%Tracer\ Uptake = \frac{Cpm(Cells)}{Cpm(Cells)+Cpm(Supernatant)+Cpm(Wash1)+Cpm(Wash2)} \quad \text{(Equation 1)}$$

Antigen-specific Treg activation assay

For Treg activation and suppression assays, two Epstein-Barr-virus-transformed B-LCLs, SPO (HLA-A*02$^+$DR11$^+$) and BM21 (HLA-A*02 DR11$^+$), were used. These cell lines were cultured in RPMI-1640 (Thermo Fisher Scientific) supplemented with 10% (v/v) heat-inactivated FBS, 100 μg/mL streptomycin, 100 IU/mL penicillin, 2 mM L-glutamine, and 1 mM sodium pyruvate (Thermo Fisher) in a humidified atmosphere containing 5% (v/v) CO_2 at 37°C. To assess CAR-specific Treg activation, B-LCLs were pretreated with a γ-rays at a dose of 40 Gy, and Tregs were cultured at a 4:1 ratio with each γ-irradiated B-LCL type (1 × 10^6 Tregs: 0.25 × 10^6 B-LCLs) in a 96-well round-bottom plate. After 18 h incubation at 37°C, cells were harvested, washed with PBS, and stained with the LIVE/DEAD dye. Cells were then washed twice and stained with PerCP-conjugated anti-CD69 antibody (Clone FN50, BioLegend, CA, USA) for 20 min at 37°C. Cells were washed twice and resuspended in flow buffer prior to flow-cytometric analysis (for antibody details and optical flow cytometer settings, see Table S3).

Antigen-specific NFAT nuclear localization assay

Treg preparations were collected before activation beads were removed magnetically and co-cultured with γ-irradiated B-LCLs at a 4:1 ratio for 30 min (1 × 10^6 Tregs: 0.25 × 10^6 B-LCLs). Subsequently, cells were collected, permeabilized/fixed, and stained for FOXP3 (see above). Permeabilization buffer containing 0.1% (v/v) Triton X-100 was used from this point onward, and the cells were first stained with the primary antibody anti-NFAT1 (D43B1 rabbit monoclonal; Cell Signaling Technology Europe; 0.26 μg/mL) for 30 min and anti-rabbit immunoglobulin G for 30 min, with a final wash and resuspension. 1 μg/L DAPI (Thermo Fisher Scientific)

was added before cell acquisition. Quantitative analysis of NFAT nuclear translocation was performed using the IDEAS image-analysis software (ImageStream/Amnis; EMD Millipore). For each cell, the nuclear (DAPI) and NFAT1 (AF700) images were compared, and a similarity score was assigned. This similarity score is a Fisher *Z* transformation of Pearson's correlation of the pixel intensity values between DAPI and NFAT1 images within each cell's nuclear region. Fluorescent markers (eGFP and mCherry) were used to confirm the different Treg types. and these along with FOXP3 were used to separate Tregs from B-LCLs.

Treg suppression assay

The suppressive capacity of Tregs was assessed by co-culturing autologous $CD4^+$ Tconv cells with varying concentrations of Tregs (ranging from 1:1 to 256:1). Frozen $CD4^+$ Tconv cells were thawed in PBS, washed thrice to remove any residual DMSO, and labeled with 2 μM CTV (Cell Proliferation Kit; Thermo Fisher Scientific) for 20 min at 37°C. Labeling was stopped by adding 20 mL of X-Vivo-15 and a further incubation for 5 min at 37°C. The labeled Tconv cells were resuspended at a concentration of 1×10^6 cells/mL in the same medium and kept on ice until needed. Treg preparations were collected, activation beads removed magnetically, washed twice with PBS, and resuspended at a concentration of 1×10^6 cells/mL. Tregs and Tconv cells were seeded (with Tregs in a serially diluted manner to achieve Treg/Tconv ratios as indicated in Figure 3) into a 96-well round-bottom plate in a total volume of 200 μL per well. Cultures were activated with γ-irradiated B-LCLs (3:1 B-LCL/Tconv ratio; 30 μL) and incubated at 37°C for 5 days. Tconv cell proliferation was measured through CTV label dilution by flow cytometry after 5 days as previously described.[11] Treg suppression was determined as a percentage defined by inverse of Tconv cell proliferation as identified when gating for CTV, relative to stimulated Tconv cells alone. For antibody details and optical flow cytometer settings, see Table S3.

Treg and monocyte co-culture assay

Monocytes were isolated from PBMCs obtained from HLA-A*02:01-positive donors using the EasySep Human CD14 Positive Selection kit (STEMCELL Technologies, Canada), according to the manufacturer's instructions. Monocytes were assessed for $CD16^+$ using flow cytometry, and only the ones with less than 5% $CD16^+$ expression were taken forward for co-culture experiments.

Co-culture experiments were performed in 48-well flat-bottom tissue culture plates. Therefore, purified monocytes (0.5×10^6 cells per well) were co-cultured with indicated Treg types (0.25×10^6 cells per well), which were generated from HLA-A*02:01-negative donors. Co-culture duration was 5 days under standard culture conditions (37°C, humidified atmosphere containing 5% [v/v] CO_2). Subsequently, cells were harvested using Accutase Cell Detachment Solution (STEMCELL Technologies), stained with LIVE/DEAD Fixable viability dye (Thermo Fisher Scientific), and subsequently labeled with fluorochrome-conjugated monoclonal antibodies targeting surface markers for flow-cytometric analysis. For antibody details and optical flow cytometer settings, see Table S4.

Cytokine analysis

Cytokine measurements were performed using LEGENDplex Human Th Cytokine Panel (BioLegend) as per manufacturer's guidelines. In brief, supernatant samples previously collected from activation and suppression assays and stored at −20°C until analysis were thawed and diluted 1:1 with the assay buffer in a V-bottom plate. Standards provided as part of the kit were also diluted 1:1 with the assay buffer and included in the V-bottom plate. Mixed capture beads were then added to the standards and samples as per manufacturer's instructions and incubated for 2 h at room temperature while mixing (thermomixer set at room temperature). Samples were washed twice and the supernatant removed, and biotinylated detection antibody cocktail was added, followed by 1 h of incubation at room temperature while mixing. Subsequently, streptavidin-phycoerythrin was added, and the samples were incubated for a further 30 min at room temperature while mixing. Samples were washed twice and resuspended in 150 μL of wash buffer before flow-cytometric data acquisition. LEGENDplex Data Analysis software was used for analysis, with individual analyte standard curves recorded in the same assay permitting quantification.

For human IL-10 and IFN-γ concentration determination in serum samples from mice taken at the humane endpoints, the BD Cytometric Bead Array (CBA) Human IL-10 Flex Set and Human IFN-γ Flex Set (both from BD Biosciences) were used according to the manufacturer's instructions. In brief, serial dilutions of the reconstituted cytokine standards were prepared to generate standard curves, and test samples were incubated with the corresponding capture beads and phycoerythrin-conjugated detection reagents for 2 h at room temperature with gentle mixing, protected from light. Following washing, the bead complexes were resuspended in assay buffer and analyzed on a flow cytometer configured for CBA detection. Data were collected until approximately 300 events per bead population were recorded, and cytokine concentrations were calculated from the respective standard curves using the FCAP Array software package.

Animals

All procedures related to animal work were performed in accordance with all legal, ethical, and institutional requirements (PPL70/7302 and PP4161226). Animal use was kept to a minimum, with cohort sizes as well as humane endpoints based on prior work. NOD.Cg-$Prkdc^{scid}$ $Il2rg^{tm1Wjl}$/SzJ (NSG) mice were purchased from Charles River UK. All mice used were female and between 8 and 12 weeks old at the beginning of experiments. Mice were maintained within the King's College London Biological Services Facility under specific pathogen-free conditions in a dedicated and licensed air-conditioned animal room (at 23°C ± 2°C and 40%–60% relative humidity) under light/dark cycles lasting 12 h every day. Mice were kept in individually ventilated standard plastic cages (IVCs; 501 cm^2 floor space; Tecniplast, UK) including environmental enrichment and bedding

material in the form of sterilized wood chips, paper strips, and one cardboard roll per cage. Maximum cage occupancy was five animals, and animals were moved to fresh cages with fresh environmental enrichment and bedding material twice per week. Sterilized tap water and food were available *ad libitum*; food was PicoLab Rodent Diet 20 (LabDiet) in the form of 2.5 × 1.6 × 1.0-cm oval pellets that were supplied at the top of the cages. Sentinel animals were kept on the same IVC racks as experimental animals and confirmed to be healthy after completion of the studies.

Xeno-GvHD mouse model

Female NSG mice underwent whole-body irradiation (150 cGy) 1 day before receiving an intravenous injection of 10×10^6 HLA-A*02$^+$ PBMCs followed by 5×10^6 engineered Tregs as indicated (injected separately). Control cohorts received either saline injections (PBS; healthy controls) or only PBMCs (GvHD positive control). No adverse events were associated with the cell administration procedures performed in this study. Healthy control animals put on weight in line with strain expectations (cf. public strain data from Charles River UK). Xeno-GvHD symptoms were scored based on weight, fur texture, posture, activity, and skin integrity, assigning from 0 to 3 points for each category, with 0 = no findings, 1 = mild, 2 = moderate, and 3 = severe, combined into a total score.[26]

To monitor human cell engraftment, peripheral blood was collected from the saphenous vein on a weekly basis and subjected to flow-cytometric analysis (for antibody details and optical flow cytometer settings, see Table S5). Upon reaching the humane endpoint (body weight loss of 15% compared to experiment accompanied by clinical signs), animals were sacrificed and indicated organs/tissue harvested. Harvested tissue samples were fixed in 10% formalin (R&D Systems, UK) and embedded in paraffin. Cut sections were deparaffinized, rehydrated, and subjected either to staining with H&E to visualize tissue structure or to immunohistochemical staining by the indicated primary antibodies (conventional antigen retrieval using citric acid was employed prior to staining). Samples were incubated with horseradish peroxidase-conjugated secondary antibodies, and the stain was developed with 3,3′-diaminobenzidine (brown stain) followed by counterstaining with hematoxylin (blue stain) and sample mounting (DPX mounting kit, Thermo Fisher). Samples were imaged using a digital-slide scanner (model C13210-04, Hamamatsu, Japan) and read blind by two pathologists.

Software and statistical analyses

Generally, data were collated in MS Excel spreadsheets and graphs generated including statistical analyses using Prism software v.10 (GraphPad, CA, USA). Flow-cytometric data were analyzed using FlowJo v.9.7.5 (BD Biosciences, USA). The LEGENDplex Data Analysis software used was v.8 (BioLegend). IDEAS Analysis Software v.6.2.187 (Amnis/EMD Millipore, USA) was used for NFAT nuclear translocation analysis. Details of statistical analyses are provided in the figure legends.

DATA AND CODE AVAILABILITY

The data presented here are available on request from the corresponding authors.

ACKNOWLEDGMENTS

We thank Drs. Anna Rose and Isabel Correa for technical assistance with flow cytometry and ImageStream analyses as well as the King’s College London (KCL) Biological Services Facility for help with animal husbandry and logistics. This work was supported by an MRC Doctoral Training Program PhD studentship to A.S., the MRC Center for Transplantation at KCL (MR/J006742/1), with important toolboxes developed with funding from Cancer Research UK (Multidisciplinary Project Award C48390/A21153 to G.O.F.). We further acknowledge funding from the Chinese Academy of Medical Sciences (CAMS) Innovation Fund for Medical Science (CIFMS) (2024-I2M-2-001-1 to F.I.). We acknowledge financial support from the Oncode Institute (supported by the Dutch Cancer Society) for Z.T., K.S., and W.P.V. and the European Joint Program Rare Diseases (TC-NER RD20-113) for W.P.V., as well as the British Heart Foundation (for G.L.). P.S. is funded by an NIHR Clinical Lectureship (CL-2018-17-002) and by the Fetal Medicine Foundation (registered charity 1037116). The authors received further support from the National Institute for Health Research (NIHR) Biomedical Research Center based at Guy’s and St Thomas’ NHS Foundation Trust. The views expressed are those of the authors and not necessarily those of the NIHR, the National Health Service, or the Department of Health.

AUTHOR CONTRIBUTIONS

Conceptualization, G.L. and G.O.F.; data curation, A.S., G.L., G.O.F., K.S., and Z.T.; formal analysis, A.S., G.O.F., W.P.V., and Z.T.; funding acquisition, G.L. and G.O.F.; investigation, A.S. and Q.P.; methodology, A.S., C.S., Q.P., P.S., W.P.V., Y.R.M., and Z.T.; project administration, G.L. and G.O.F.; resources, F.I., G.L., G.O.F., Q.P., and Y.R.M.; supervision, G.L. and G.O.F.; validation, A.S., G.L., and G.O.F.; visualization, G.O.F.; writing – original draft, G.O.F.; writing – review and editing, all authors.

DECLARATION OF INTERESTS

G.L. is a founder of Quell Therapeutics and a member of its scientific advisory board. She is married to Prof. Sir Robert Lechler, who is a non-executive director of Quell Therapeutics. G.O.F. is an associate editor for *Molecular Therapy*.

SUPPLEMENTAL INFORMATION

Supplemental information can be found online at https://doi.org/10.1016/j.ymthe.2026.02.001.

REFERENCES

1. Sakaguchi, S., Miyara, M., Costantino, C.M., and Hafler, D.A. (2010). FOXP3+ regulatory T cells in the human immune system. Nat. Rev. Immunol. *10*, 490–500. https://doi.org/10.1038/nri2785.

2. Tang, Q., and Vincenti, F. (2017). Transplant trials with Tregs: perils and promises. J. Clin. Invest. *127*, 2505–2512. https://doi.org/10.1172/JCI90598.

3. Romano, M., Fanelli, G., Albany, C.J., Giganti, G., and Lombardi, G. (2019). Past, Present, and Future of Regulatory T Cell Therapy in Transplantation and Autoimmunity. Front. Immunol. *10*, 43. https://doi.org/10.3389/fimmu.2019.00043.

4. Sanchez-Fueyo, A., Whitehouse, G., Grageda, N., Cramp, M.E., Lim, T.Y., Romano, M., Thirkell, S., Lowe, K., Fry, L., Heward, J., et al. (2020). Applicability, safety, and biological activity of regulatory T cell therapy in liver transplantation. Am. J. Transpl. *20*, 1125–1136. https://doi.org/10.1111/ajt.15700.

5. Sawitzki, B., Harden, P.N., Reinke, P., Moreau, A., Hutchinson, J.A., Game, D.S., Tang, Q., Guinan, E.C., Battaglia, M., Burlingham, W.J., et al. (2020). Regulatory cell therapy in kidney transplantation (The ONE Study): a harmonised design and analysis of seven non-randomised, single-arm, phase 1/2A trials. Lancet *395*, 1627–1639. https://doi.org/10.1016/S0140-6736(20)30167-7.

6. Harden, P.N., Game, D.S., Sawitzki, B., Van der Net, J.B., Hester, J., Bushell, A., Issa, F., Brook, M.O., Alzhrani, A., Schlickeiser, S., et al. (2021). Feasibility, long-term safety, and immune monitoring of regulatory T cell therapy in living donor kidney transplant recipients. Am. J. Transpl. *21*, 1603–1611. https://doi.org/10.1111/ajt.16395.

7. Shen, X.-F., Jiang, J.-P., Yang, J.-J., Wang, W.-Z., Guan, W.-X., and Du, J.-F. (2016). Donor-Specific Regulatory T Cells Acquired from Tolerant Mice Bearing Cardiac Allograft Promote Mixed Chimerism and Prolong Intestinal Allograft Survival. Front. Immunol. *7*, 511. https://doi.org/10.3389/fimmu.2016.00511.

8. Sagoo, P., Ali, N., Garg, G., Nestle, F.O., Lechler, R.I., and Lombardi, G. (2011). Human regulatory T cells with alloantigen specificity are more potent inhibitors of alloimmune skin graft damage than polyclonal regulatory T cells. Sci. Transl. Med. *3*, 83ra42. https://doi.org/10.1126/scitranslmed.3002076.

9. Putnam, A.L., Safinia, N., Medvec, A., Laszkowska, M., Wray, M., Mintz, M.A., Trotta, E., Szot, G.L., Liu, W., Lares, A., et al. (2013). Clinical grade manufacturing of human alloantigen-reactive regulatory T cells for use in transplantation. Am. J. Transpl. *13*, 3010–3020. https://doi.org/10.1111/ajt.12433.

10. Sicard, A., Levings, M.K., and Scott, D.W. (2018). Engineering therapeutic T cells to suppress alloimmune responses using TCRs, CARs, or BARs. Am. J. Transpl. *18*, 1305–1311. https://doi.org/10.1111/ajt.14747.

11. Boardman, D.A., Philippeos, C., Fruhwirth, G.O., Ibrahim, M.A.A., Hannen, R.F., Cooper, D., Marelli-Berg, F.M., Watt, F.M., Lechler, R.I., Maher, J., et al. (2017). Expression of a Chimeric Antigen Receptor Specific for Donor HLA Class I Enhances the Potency of Human Regulatory T Cells in Preventing Human Skin Transplant Rejection. Am. J. Transpl. *17*, 931–943. https://doi.org/10.1111/ajt.14185.

12. MacDonald, K.G., Hoeppli, R.E., Huang, Q., Gillies, J., Luciani, D.S., Orban, P.C., Broady, R., and Levings, M.K. (2016). Alloantigen-specific regulatory T cells generated with a chimeric antigen receptor. J. Clin. Invest. *126*, 1413–1424. https://doi.org/10.1172/JCI82771.

13. Noyan, F., Zimmermann, K., Hardtke-Wolenski, M., Knoefel, A., Schulde, E., Geffers, R., Hust, M., Huehn, J., Galla, M., Morgan, M., et al. (2017). Prevention of Allograft Rejection by Use of Regulatory T Cells With an MHC-Specific Chimeric Antigen Receptor. Am. J. Transpl. *17*, 917–930. https://doi.org/10.1111/ajt.14175.

14. Chmielewski, M., Kopecky, C., Hombach, A.A., and Abken, H. (2011). IL-12 release by engineered T cells expressing chimeric antigen receptors can effectively Muster an antigen-independent macrophage response on tumor cells that have shut down tumor antigen expression. Cancer Res. *71*, 5697–5706. https://doi.org/10.1158/0008-5472.CAN-11-0103.

15. Chmielewski, M., and Abken, H. (2017). CAR T Cells Releasing IL-18 Convert to T-Bet(high) FoxO1(low) Effectors that Exhibit Augmented Activity against Advanced Solid Tumors. Cell Rep. *21*, 3205–3219. https://doi.org/10.1016/j.celrep.2017.11.063.

16. Suarez, E.R., Chang, D.K., Sun, J., Sui, J., Freeman, G.J., Signoretti, S., Zhu, Q., and Marasco, W.A. (2016). Chimeric antigen receptor T cells secreting anti-PD-L1 antibodies more effectively regress renal cell carcinoma in a humanized mouse model. Oncotarget *7*, 34341–34355. https://doi.org/10.18632/oncotarget.9114.

17. Li, S., Siriwon, N., Zhang, X., Yang, S., Jin, T., He, F., Kim, Y.J., Mac, J., Lu, Z., Wang, S., et al. (2017). Enhanced Cancer Immunotherapy by Chimeric Antigen Receptor-Modified T Cells Engineered to Secrete Checkpoint Inhibitors. Clin. Cancer Res. *23*, 6982–6992. https://doi.org/10.1158/1078-0432.CCR-17-0867.

18. Di Stasi, A., Tey, S.K., Dotti, G., Fujita, Y., Kennedy-Nasser, A., Martinez, C., Straathof, K., Liu, E., Durett, A.G., Grilley, B., et al. (2011). Inducible apoptosis as a safety switch for adoptive cell therapy. N. Engl. J. Med. *365*, 1673–1683. https://doi.org/10.1056/NEJMoa1106152.

19. Volpe, A., Lang, C., Lim, L., Man, F., Kurtys, E., Ashmore-Harris, C., Johnson, P., Skourti, E., de Rosales, R.T.M., and Fruhwirth, G.O. (2020). Spatiotemporal PET Imaging Reveals Differences in CAR-T Tumor Retention in Triple-Negative Breast Cancer Models. Mol. Ther. *28*, 2271–2285. https://doi.org/10.1016/j.ymthe.2020.06.028.

20. Minn, I., Huss, D.J., Ahn, H.H., Chinn, T.M., Park, A., Jones, J., Brummet, M., Rowe, S.P., Sysa-Shah, P., Du, Y., et al. (2019). Imaging CAR T cell therapy with PSMA-targeted positron emission tomography. Sci. Adv. *5*, eaaw5096. https://doi.org/10.1126/sciadv.aaw5096.

21. Mohseni, Y.R., Saleem, A., Tung, S.L., Dudreuilh, C., Lang, C., Peng, Q., Volpe, A., Adigbli, G., Cross, A., Hester, J., et al. (2021). Chimeric antigen receptor-modified human regulatory T cells that constitutively express IL-10 maintain their phenotype and are potently suppressive. Eur. J. Immunol. *51*, 2522–2530. https://doi.org/10.1002/eji.202048934.

22. Wang, Z., Guan, D., Huo, J., Biswas, S.K., Huang, Y., Yang, Y., Xu, S., and Lam, K.-P. (2021). IL-10 Enhances Human Natural Killer Cell Effector Functions via Metabolic Reprogramming Regulated by mTORC1 Signaling. Front. Immunol. *12*, 619195. https://doi.org/10.3389/fimmu.2021.619195.

23. Carlini, V., Noonan, D.M., Abdalalem, E., Goletti, D., Sansone, C., Calabrone, L., and Albini, A. (2023). The multifaceted nature of IL-10: regulation, role in immunological homeostasis and its relevance to cancer, COVID-19 and post-COVID conditions. Front. Immunol. *14*, 1161067. https://doi.org/10.3389/fimmu.2023.1161067.

24. Whitehouse, G., Gray, E., Mastoridis, S., Merritt, E., Kodela, E., Yang, J.H.M., Danger, R., Mairal, M., Christakoudi, S., Lozano, J.J., et al. (2017). IL-2 therapy restores regulatory T-cell dysfunction induced by calcineurin inhibitors. Proc. Natl. Acad. Sci. USA *114*, 7083–7088. https://doi.org/10.1073/pnas.1620835114.

25. Romano, M., Fanelli, G., Tan, N., Nova-Lamperti, E., McGregor, R., Lechler, R.I., Lombardi, G., and Scottà, C. (2018). Expanded Regulatory T Cells Induce Alternatively Activated Monocytes With a Reduced Capacity to Expand T Helper-17 Cells. Front. Immunol. *9*, 1625. https://doi.org/10.3389/fimmu.2018.01625.

26. Dawson, N.A.J., Rosado-Sanchez, I., Novakovsky, G.E., Fung, V.C.W., Huang, Q., McIver, E., Sun, G., Gillies, J., Speck, M., Orban, P.C., et al. (2020). Functional effects of chimeric antigen receptor co-receptor signaling domains in human regulatory T cells. Sci. Transl. Med. *12*, eaaz3866. https://doi.org/10.1126/scitranslmed.aaz3866.

27. Jacob, J., Nadkarni, S., Volpe, A., Peng, Q., Tung, S.L., Hannen, R.F., Mohseni, Y.R., Scotta, C., Marelli-Berg, F.M., Lechler, R.I., et al. (2021). Spatiotemporal in vivo tracking of polyclonal human regulatory T cells (Tregs) reveals a role for innate immune cells in Treg transplant recruitment. Mol. Ther. Methods Clin. Dev. *20*, 324–336. https://doi.org/10.1016/j.omtm.2020.12.003.

28. Ponomarev, V., Doubrovin, M., Lyddane, C., Beresten, T., Balatoni, J., Bornman, W., Finn, R., Akhurst, T., Larson, S., Blasberg, R., et al. (2001). Imaging TCR-dependent NFAT-mediated T-cell activation with positron emission tomography in vivo. Neoplasia *3*, 480–488. https://doi.org/10.1038/sj.neo.7900204.

29. Boardman, D.A., Mangat, S., Gillies, J.K., Leon, L., Fung, V.C.W., Haque, M., Mojibian, M., Halvorson, T., Huang, Q., Tuomela, K., et al. (2025). Armored human CAR Treg cells with PD1 promoter-driven IL-10 have enhanced suppressive function. Sci. Adv. *11*. eadx7845. https://doi.org/10.1126/sciadv.adx7845.

30. Morsut, L., Roybal, K.T., Xiong, X., Gordley, R.M., Coyle, S.M., Thomson, M., and Lim, W.A. (2016). Engineering Customized Cell Sensing and Response Behaviors Using Synthetic Notch Receptors. Cell *164*, 780–791. https://doi.org/10.1016/j.cell.2016.01.012.

31. Zhu, I., Liu, R., Garcia, J.M., Hyrenius-Wittsten, A., Piraner, D.I., Alavi, J., Israni, D.V., Liu, B., Khalil, A.S., and Roybal, K.T. (2022). Modular design of synthetic receptors for programmed gene regulation in cell therapies. Cell *185*, 1431–1443.e16. https://doi.org/10.1016/j.cell.2022.03.023.

32. Goyco Vera, D., Waghela, H., Nuh, M., Pan, J., and Lulla, P. (2024). Approved CAR-T therapies have reproducible efficacy and safety in clinical practice. Hum. Vaccin. Immunother. *20*, 2378543. https://doi.org/10.1080/21645515.2024.2378543.

33. Mackensen, A., Müller, F., Mougiakakos, D., Böltz, S., Wilhelm, A., Aigner, M., Völkl, S., Simon, D., Kleyer, A., Munoz, L., et al. (2022). Anti-CD19 CAR T cell therapy for refractory systemic lupus erythematosus. Nat. Med. *28*, 2124–2132. https://doi.org/10.1038/s41591-022-02017-5.

34. Guffroy, A., Jacquel, L., Guffroy, B., and Martin, T. (2024). CAR-T cells for treating systemic lupus erythematosus: A promising emerging therapy. Joint Bone Spine *91*, 105702. https://doi.org/10.1016/j.jbspin.2024.105702.

35. Kumar, M., Keller, B., Makalou, N., and Sutton, R.E. (2001). Systematic determination of the packaging limit of lentiviral vectors. Hum. Gene Ther. *12*, 1893–1905. https://doi.org/10.1089/104303401753153947.

36. al Yacoub, N., Romanowska, M., Haritonova, N., and Foerster, J. (2007). Optimized production and concentration of lentiviral vectors containing large inserts. J. Gene Med. *9*, 579–584. https://doi.org/10.1002/jgm.1052.

37. Van Zeebroeck, L., Arroyo Hornero, R., Côrte-Real, B.F., Hamad, I., Meissner, T.B., and Kleinewietfeld, M. (2021). Fast and Efficient Genome Editing of Human FOXP3(+) Regulatory T Cells. Front. Immunol. *12*, 655122. https://doi.org/10.3389/fimmu.2021.655122.

38. Du, W., Noyan, F., McCallion, O., Drosdek, V., Kath, J., Glaser, V., Fuster-Garcia, C., Yang, M., Stein, M., Franke, C., et al. (2025). Gene editing of CD3 epsilon to redirect regulatory T cells for adoptive T cell transfer. Mol. Ther. *33*, 997–1013. https://doi.org/10.1016/j.ymthe.2025.01.045.

39. Lam, A.J., Lin, D.T.S., Gillies, J.K., Uday, P., Pesenacker, A.M., Kobor, M.S., and Levings, M.K. (2021). Optimized CRISPR-mediated gene knockin reveals FOXP3-independent maintenance of human Treg identity. Cell Rep. *36*, 109494. https://doi.org/10.1016/j.celrep.2021.109494.

40. Ashmore-Harris, C., Ayabe, H., Yoshizawa, E., Arisawa, T., Takada, Y., Takebe, T., and Fruhwirth, G.O. (2025). Gene editing enables non-invasive in vivo PET imaging of human induced pluripotent stem cell-derived liver bud organoids. Mol. Ther. Methods Clin. Dev. *33*, 101406. https://doi.org/10.1016/j.omtm.2025.101406.

41. Anzalone, A.V., Koblan, L.W., and Liu, D.R. (2020). Genome editing with CRISPR-Cas nucleases, base editors, transposases and prime editors. Nat. Biotechnol. *38*, 824–844. https://doi.org/10.1038/s41587-020-0561-9.

42. Bak, R.O., and Porteus, M.H. (2017). CRISPR-Mediated Integration of Large Gene Cassettes Using AAV Donor Vectors. Cell Rep. *20*, 750–756. https://doi.org/10.1016/j.celrep.2017.06.064.

43. Chaudhari, N., Rickard, A.M., Roy, S., Dröge, P., and Makhija, H. (2020). A non-viral genome editing platform for site-specific insertion of large transgenes. Stem Cell Res. Ther. *11*, 380. https://doi.org/10.1186/s13287-020-01890-6.

44. Wang, P., Wu, P., Siegel, M.I., Egan, R.W., and Billah, M.M. (1995). Interleukin (IL)-10 inhibits nuclear factor kappa B (NF kappa B) activation in human monocytes. IL-10 and IL-4 suppress cytokine synthesis by different mechanisms. J. Biol. Chem. *270*, 9558–9563. https://doi.org/10.1074/jbc.270.16.9558.

45. Muller, Y.D., Ferreira, L.M.R., Ronin, E., Ho, P., Nguyen, V., Faleo, G., Zhou, Y., Lee, K., Leung, K.K., Skartsis, N., et al. (2021). Precision Engineering of an Anti-HLA-A2 Chimeric Antigen Receptor in Regulatory T Cells for Transplant Immune Tolerance. Front. Immunol. *12*, 686439. https://doi.org/10.3389/fimmu.2021.686439.

46. Ehx, G., Somja, J., Warnatz, H.J., Ritacco, C., Hannon, M., Delens, L., Fransolet, G., Delvenne, P., Muller, J., Beguin, Y., et al. (2018). Xenogeneic Graft-Versus-Host Disease in Humanized NSG and NSG-HLA-A2/HHD Mice. Front. Immunol. *9*, 1943. https://doi.org/10.3389/fimmu.2018.01943.

47. Abraham, S., Choi, J.G., Ye, C., Manjunath, N., and Shankar, P. (2015). IL-10 exacerbates xenogeneic GVHD by inducing massive human T cell expansion. Clin. Immunol. *156*, 58–64. https://doi.org/10.1016/j.clim.2014.11.004.

48. Ali, N., Flutter, B., Sanchez Rodriguez, R., Sharif-Paghaleh, E., Barber, L.D., Lombardi, G., and Nestle, F.O. (2012). Xenogeneic graft-versus-host-disease in NOD-scid IL-2Rgammanull mice display a T-effector memory phenotype. PLoS One *7*, e44219. https://doi.org/10.1371/journal.pone.0044219.

49. Hsueh, Y.H., Chen, H.W., Syu, B.J., Lin, C.I., Leung, P.S.C., Gershwin, M.E., and Chuang, Y.H. (2018). Endogenous IL-10 maintains immune tolerance but IL-10 gene transfer exacerbates autoimmune cholangitis. J. Autoimmun. *95*, 159–170. https://doi.org/10.1016/j.jaut.2018.09.009.

50. Emmerich, J., Mumm, J.B., Chan, I.H., LaFace, D., Truong, H., McClanahan, T., Gorman, D.M., and Oft, M. (2012). IL-10 directly activates and expands tumor-resident CD8(+) T cells without de novo infiltration from secondary lymphoid organs. Cancer Res. *72*, 3570–3581. https://doi.org/10.1158/0008-5472.CAN-12-0721.

51. Chen, K.Y., Liu, J., and Ren, E.C. (2012). Structural and functional distinctiveness of HLA-A2 allelic variants. Immunol. Res. *53*, 182–190. https://doi.org/10.1007/s12026-012-8295-5.

52. Volpe, A., Man, F., Lim, L., Khoshnevisan, A., Blower, J., Blower, P.J., and Fruhwirth, G.O. (2018). Radionuclide-fluorescence Reporter Gene Imaging to Track Tumor Progression in Rodent Tumor Models. J. Vis. Exp. *133*, e57088. https://doi.org/10.3791/57088.

53. Schroder, A.R., Shinn, P., Chen, H., Berry, C., Ecker, J.R., and Bushman, F. (2002). HIV-1 integration in the human genome favors active genes and local hotspots. Cell *110*, 521–529. https://doi.org/10.1016/s0092-8674(02)00864-4.

54. Shao, L., Shi, R., Zhao, Y., Liu, H., Lu, A., Ma, J., Cai, Y., Fuksenko, T., Pelayo, A., Shah, N.N., et al. (2022). Genome-wide profiling of retroviral DNA integration and its effect on clinical pre-infusion CAR T-cell products. J. Transl. Med. *20*, 514. https://doi.org/10.1186/s12967-022-03729-5.

55. Arber, C., Abhyankar, H., Heslop, H.E., Brenner, M.K., Liu, H., Dotti, G., and Savoldo, B. (2013). The immunogenicity of virus-derived 2A sequences in immunocompetent individuals. Gene Ther. *20*, 958–962. https://doi.org/10.1038/gt.2013.25.

YMTHE, Volume *34*

Supplemental Information

CAR-mediated release of IL-10 increases the function of regulatory T cells: Relevance for future clinical application

Adeel Saleem, Qi Peng, Ziqin Tang, Yasmin R. Mohseni, Cristiano Scottà, Panicos Shangaris, Kimberly Smit, Wilbert P. Vermeij, Fadi Issa, Giovanna Lombardi, and Gilbert O. Fruhwirth

SUPPLEMENTAL FIGURES

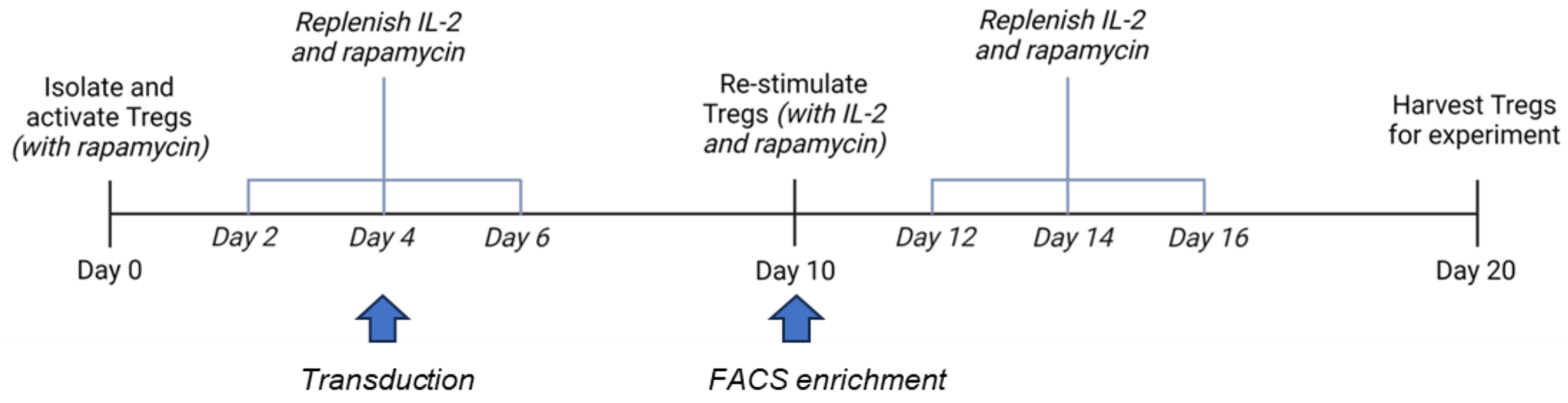

Figure S1. Scheme of Treg isolation, transduction, enrichment, and expansion. | IL-2 and rapamycin were replenished every two days after transduction with viral vectors. Transduction was performed on day 4 and FACS enrichment on day 10. Cells were harvested on day 20 for subsequent analyses or to use for *in vivo* experiments.

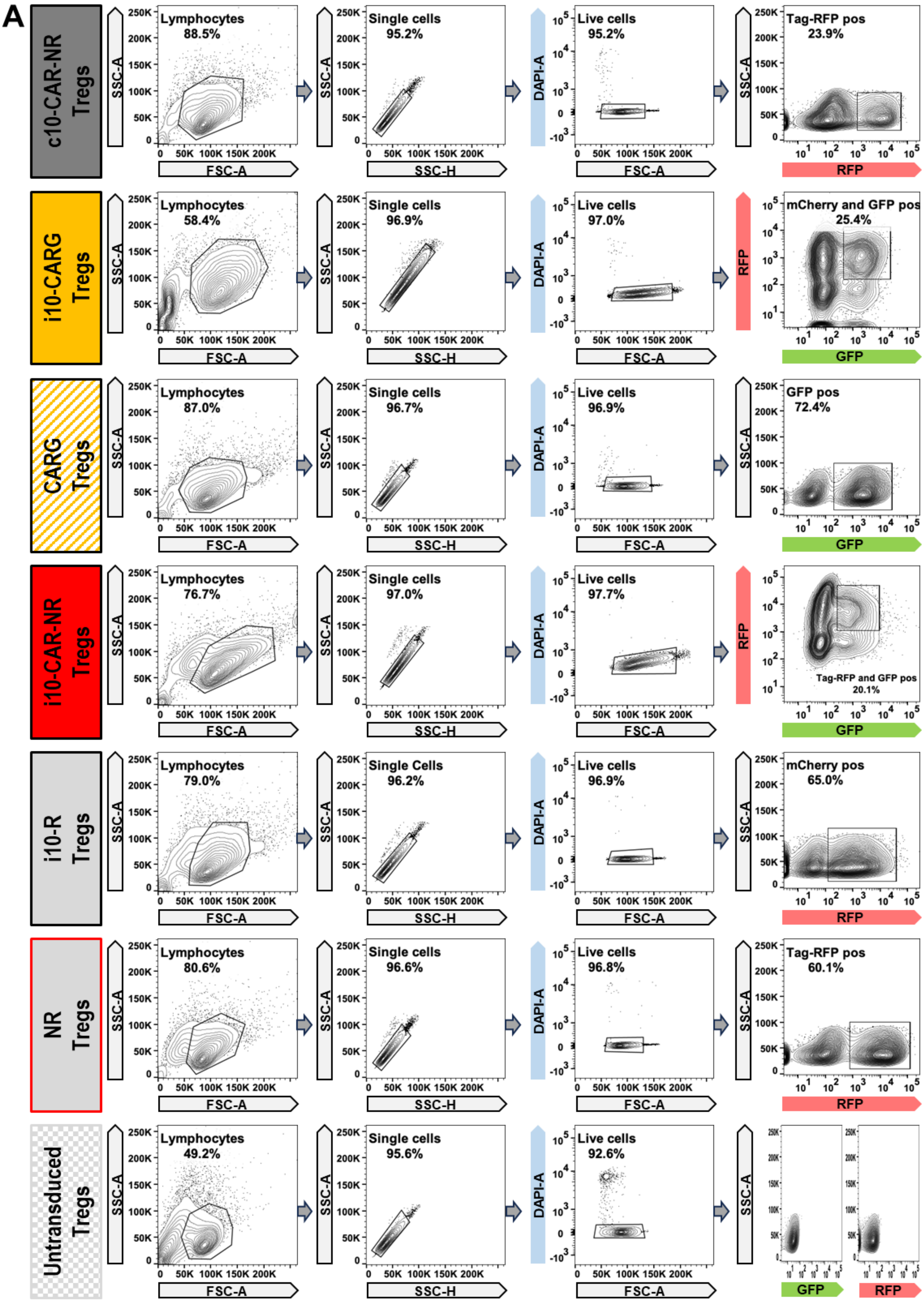
A
c10-CAR-NR Tregs
Lymphocytes 88.5%
Single cells 95.2%
Live cells 95.2%
Tag-RFP pos 23.9%
i10-CARG Tregs
Lymphocytes 58.4%
Single cells 96.9%
Live cells 97.0%
mCherry and GFP pos 25.4%
CARG Tregs
Lymphocytes 87.0%
Single cells 96.7%
Live cells 96.9%
GFP pos 72.4%
i10-CAR-NR Tregs
Lymphocytes 76.7%
Single cells 97.0%
Live cells 97.7%
Tag-RFP and GFP pos 20.1%
i10-R Tregs
Lymphocytes 79.0%
Single Cells 96.2%
Live cells 96.9%
mCherry pos 65.0%
NR Tregs
Lymphocytes 80.6%
Single cells 96.6%
Live cells 96.8%
Tag-RFP pos 60.1%
Untransduced Tregs
Lymphocytes 49.2%
Single cells 95.6%
Live cells 92.6%
SSC-A
FSC-A
SSC-H
DAPI-A
RFP
GFP

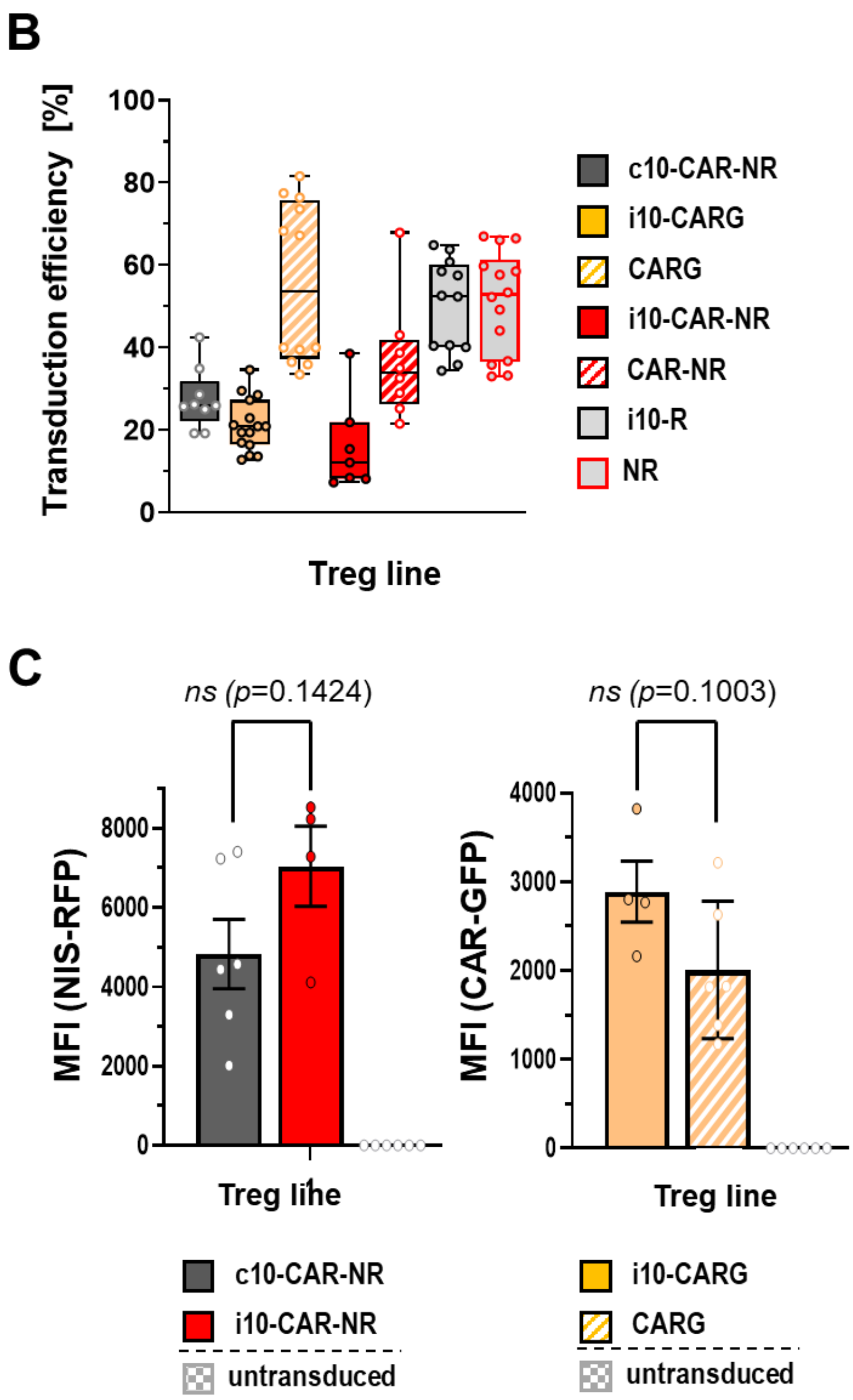

Figure S2. Representative gating strategy for evaluating transduction efficiencies. | (A) Representative flow cytometry plots showing the gating strategy for evaluating fluorescent protein expression (*cf.* Fig.1A) with examples from different engineered Treg types. Cells were analysed one week after transduction on day 10. **(B)** Cumulative transduction efficiency data based on the corresponding fluorescent protein reporter expression on day 10 and prior to FACS sorting of the various Treg populations. Box-an-Whisker plot shown with middle line indicating the medians and minima/maxima indicated by 'error bar' style lines. **(C)** CAR protein levels in the various CAR-Treg lines were determined by flow cytometry. *(Right)* i10-CARG and CARG Tregs expressed the anti-HLA-A*02 targeted CAR fused to GFP (see Fig.1A) and green fluorescence was a direct measure for CAR protein. *(Left)* In i10-CAR-NR and c10-CAR-NR Treg lines, anti-HLA-A*02 CAR expression was from the same transcript as NIS-RFP (see Fig.1), hence a surrogate for protein production from this transcript and therefore a measure for the CAR protein level. Mean fluorescence intensity (MFI) was determined on day 20 and is shown. Student's *t*-tests were used to evaluate any potential differences (α=0.05, two-tailed, heteroscedastic).

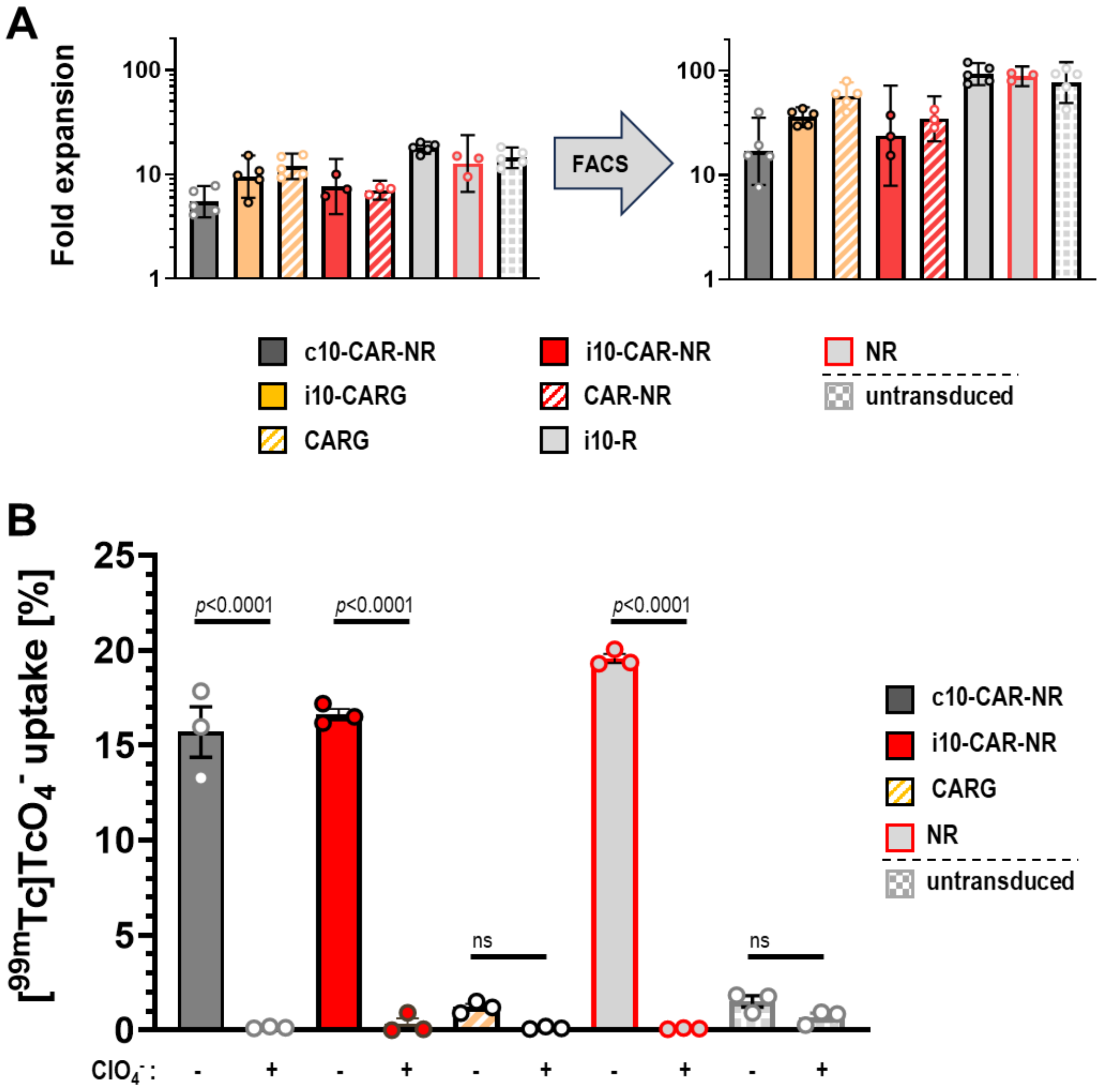

Figure S3. Expansion capacity and evaluation of radionuclide reporter function of engineered Tregs. | (A) Expansion capacity for indicated engineered Tregs for (*left*) the period between transduction and FACS sorting on day 10 and (*right*) for the period from FACS sorting until day 20. For timelines, see Figure S1. Geometric means are show with error bars representing 95% confidence intervals. **(B)** i10-CAR-NR, c10-CAR-NR, and NR Tregs (positive control) additionally contained the NIS-RFP reporter, which enables fluorescence applications but also future in vivo cell tracking by radionuclide imaging as demonstrated for Tregs before.[1] The function of the NIS portion of this dual–mode fusion reporter was determined by uptake of the NIS-specific substrate Tc-99m-pertechnetate (50kBq, 30min). The competitive substrate perchlorate was used to demonstrate that radiotracer uptake was specific to NIS-RFP. N=3 independent Treg batches from different donors with error bars representing SEM. Statistical significance was determined by Student's *t*-test (α=0.05, two-tailed, heteroscedastic) between sample pairs with and without perchlorate addition.

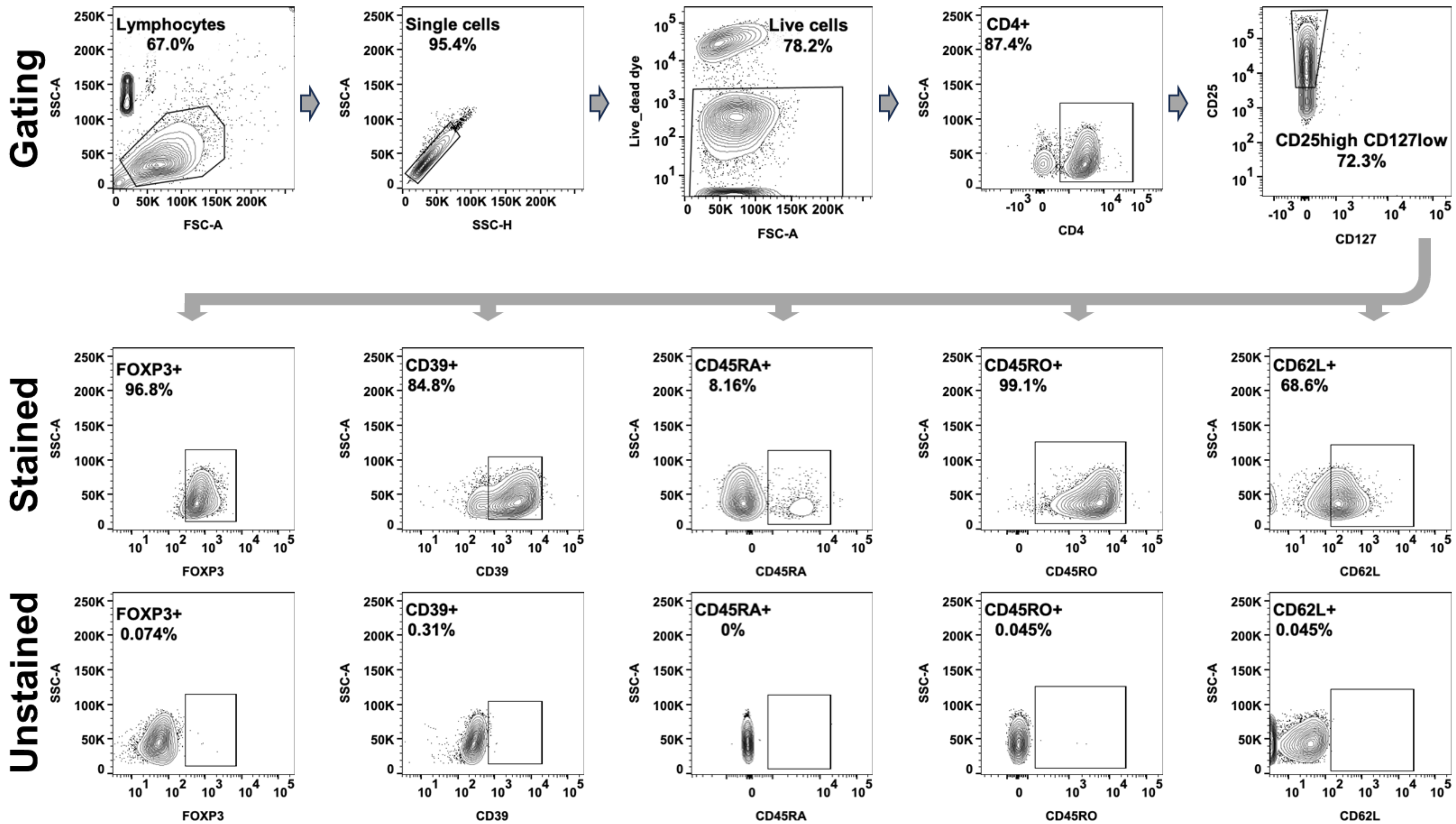

Figure S4. Representative Treg phenotyping example depicting gating strategy for indicated Treg markers. | Gating and subsequent representative results of stained and unstained samples for various Treg function and homing markers.

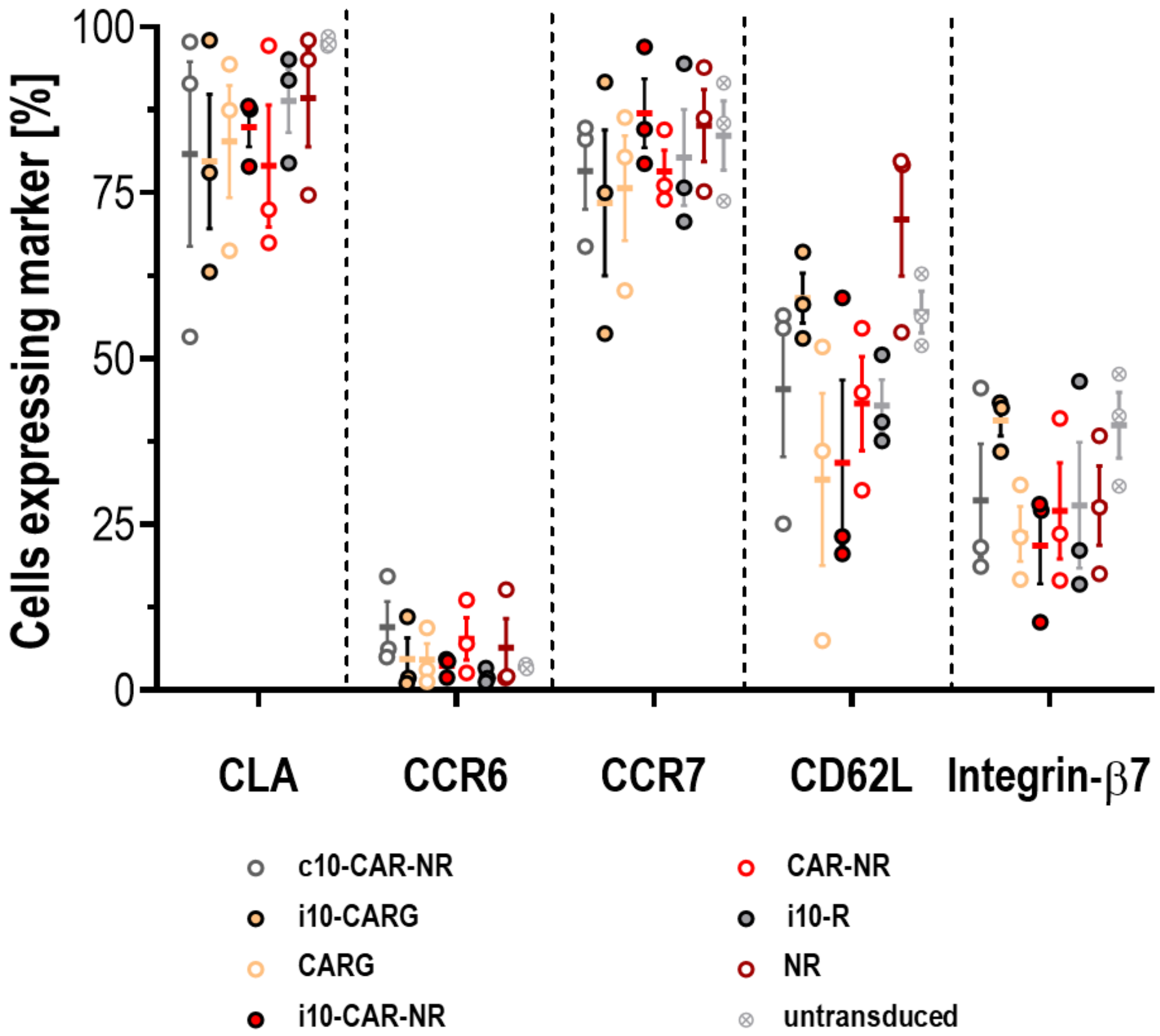

Figure S5. Surface expression of homing markers for Tregs. | Cumulative flow cytometry data for indicated marker expression in the various Treg types. N=3 independent Treg batches each with mean and error bars representing SEM.

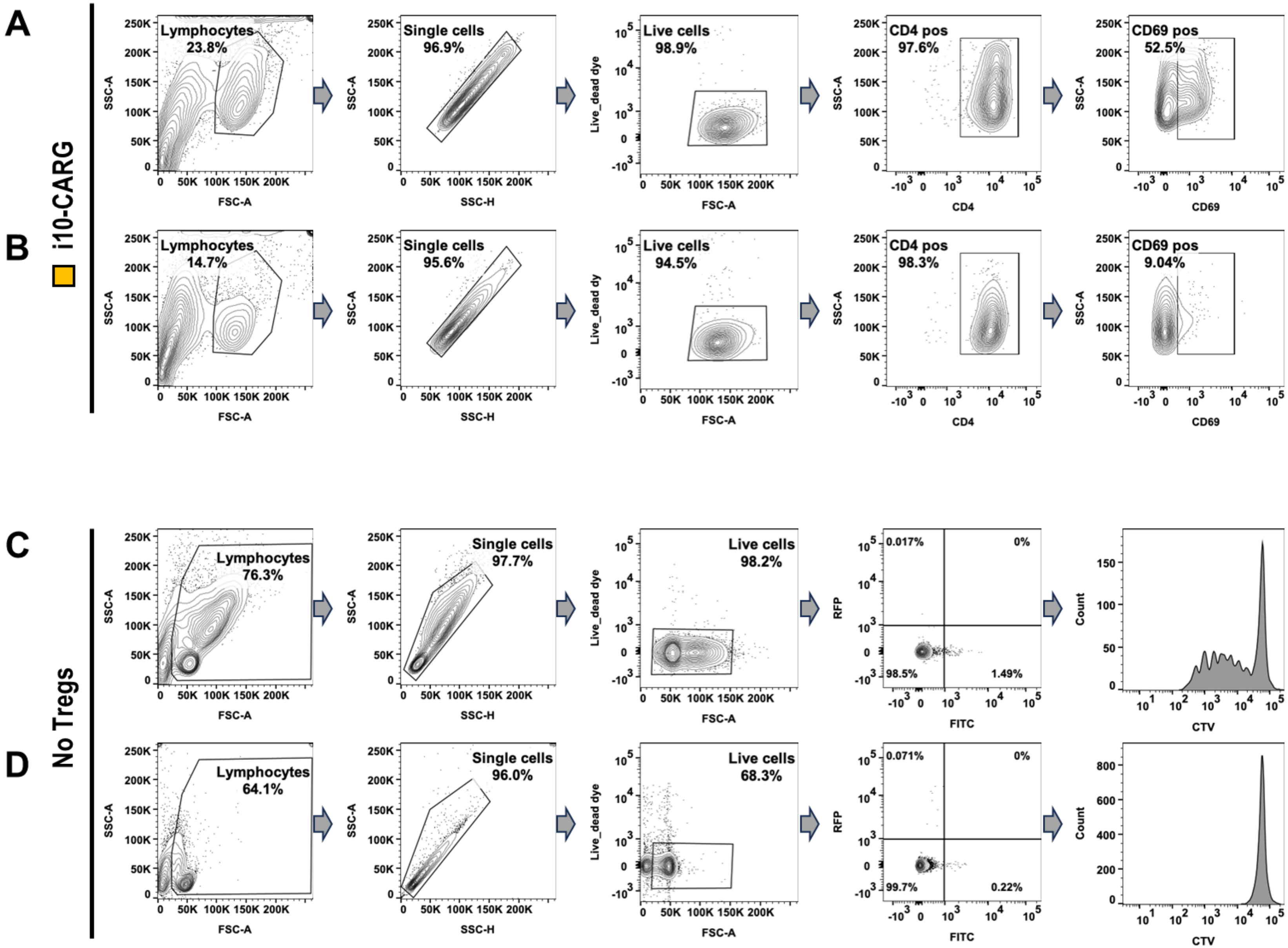
A
B
i10-CARG
C
D
No Tregs
Lymphocytes 23.8%
Single cells 96.9%
Live cells 98.9%
CD4 pos 97.6%
CD69 pos 52.5%
Lymphocytes 14.7%
Single cells 95.6%
Live cells 94.5%
CD4 pos 98.3%
CD69 pos 9.04%
Lymphocytes 76.3%
Single cells 97.7%
Live cells 98.2%
0.017%
0%
98.5%
1.49%
Lymphocytes 64.1%
Single cells 96.0%
Live cells 68.3%
0.071%
0%
99.7%
0.22%
SSC-A
FSC-A
SSC-H
Live_dead dye
CD4
CD69
RFP
FITC
Count
CTV

Figure S6. Representative gating strategy for determining the activation status of engineered Tregs and suppressive capacity towards Tconv. | i10-CARG Tregs were exposed to **(A)** the A2-CAR target antigen HLA-A*02 or **(B)** the mis-match antigen and Treg activation was measured by determining CD69 activation marker expression using flow cytometry (after 24h co-culture with corresponding B-LCLs). **(C)** Representative gating for Cell Trace Violet (CTV)-labelled Tconv that were stimulated with CD3/28 beads and consequently proliferated. **(D)** Representative gating for unstimulated CTV labelled Tconv, which did not proliferate. (C/D) are typical controls for assays that additionally contain Tregs (see Fig.3).

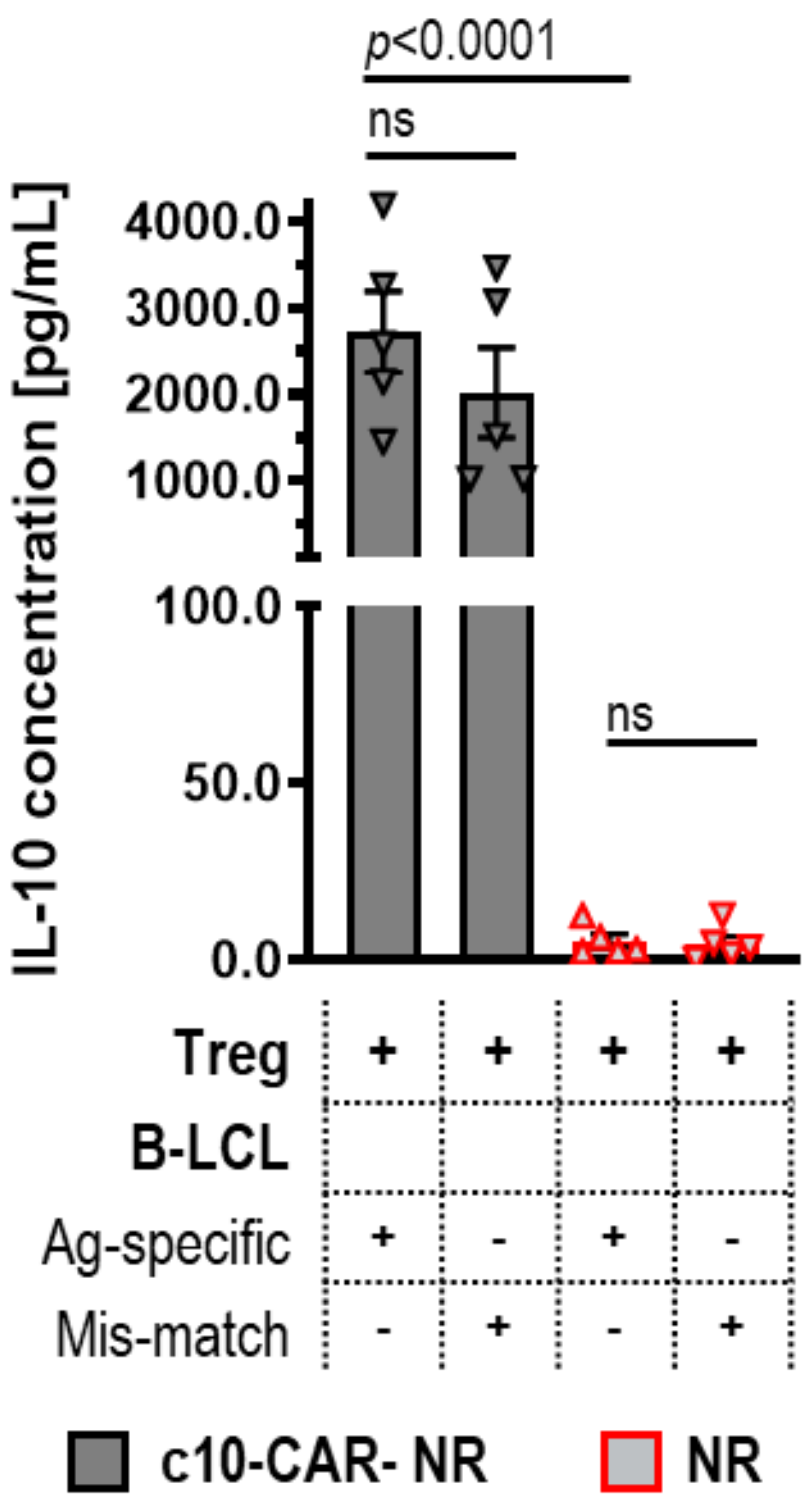

Figure S7. CD69 expression and IL-10 secretion data complementing Fig. 2C. | Indicated Treg types were co-cultured for 18h with B-LCL expressing either HLA-A*02 or the mis-match antigen HLA-A*01. IL-10 concentration was determined in co-culture supernatants of indicated engineered Tregs with HLA-A*02 B-LCLs or mis-match HLA-A*01 B-LCLs as indicated. N=5 Treg batches from different donors with error bars representing SEM. Statistical analyses in (A-B) were performed by one-way ANOVA with Tukey's multiple comparison correction and α=0.05 for all panels**.**

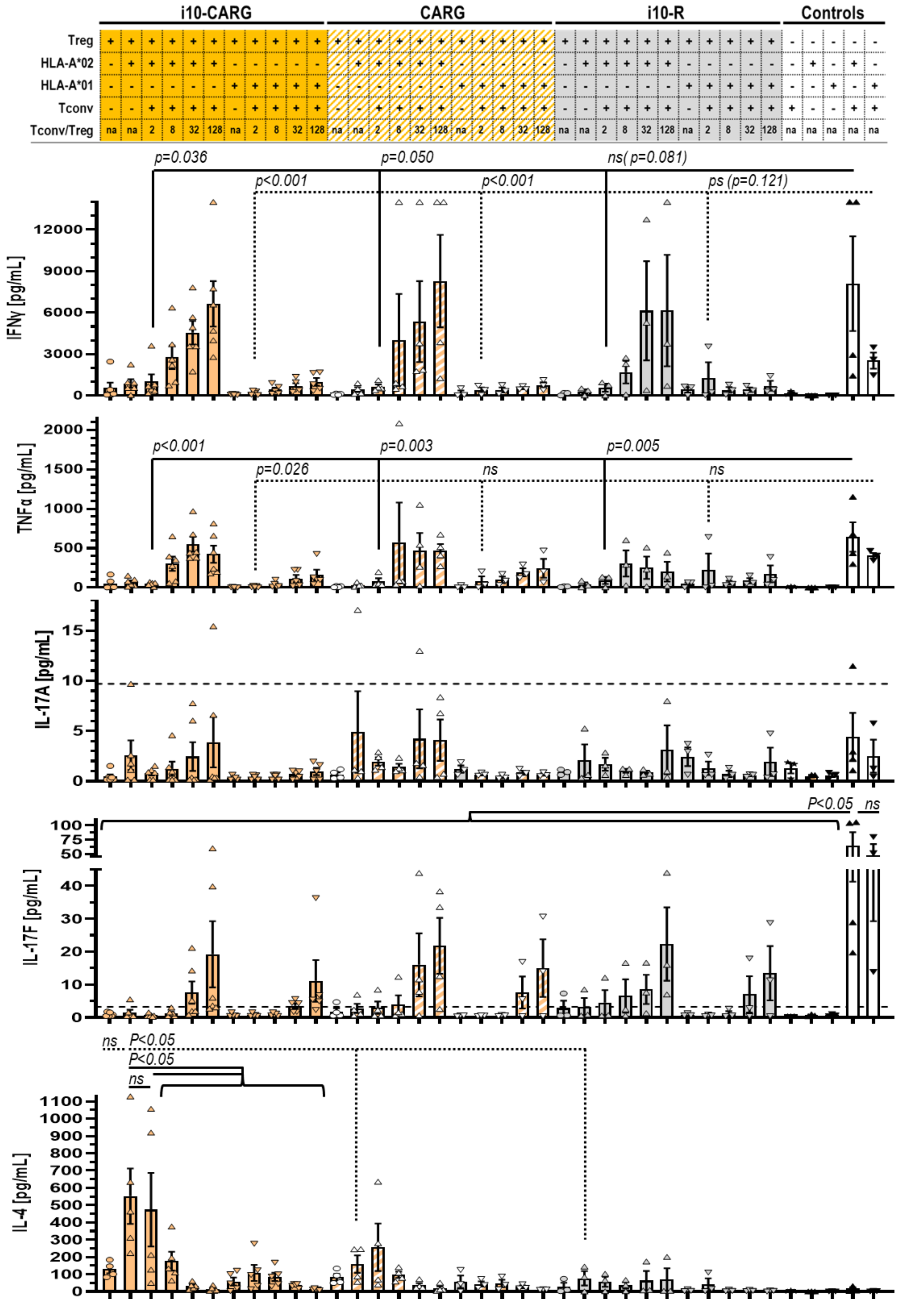

Figure S8. Cytokine secretion data supplementing suppression assays from Fig.3. | Secretion of additional indicated cytokines in the culture supernatants of various Treg suppression assays as assessed after 5 days of co-culture with autologous Tconv and

antigen presenting cells that were either HLA-A*02 positive B-LCLs (SPO) or HLA-A*02 negative B-LCLs (BM21). Cytokine bead array data are shown with means and error bars representing SEM; N ranged from 3-6. Cytokine secretion data from the supernatant of Tregs alone, Tregs with B-LCLs alone (without Tconv), B-LCLs alone, Tconv alone, and Tconv with B-LCLs (without Tregs) are included as controls. Statistical significance was tested by one-way ANOVA with multiple comparison correction, and the most relevant significant comparisons are visualised. The dashed line in the middle panel indicates the lower Limit of Measurement in the IL-17 assays.

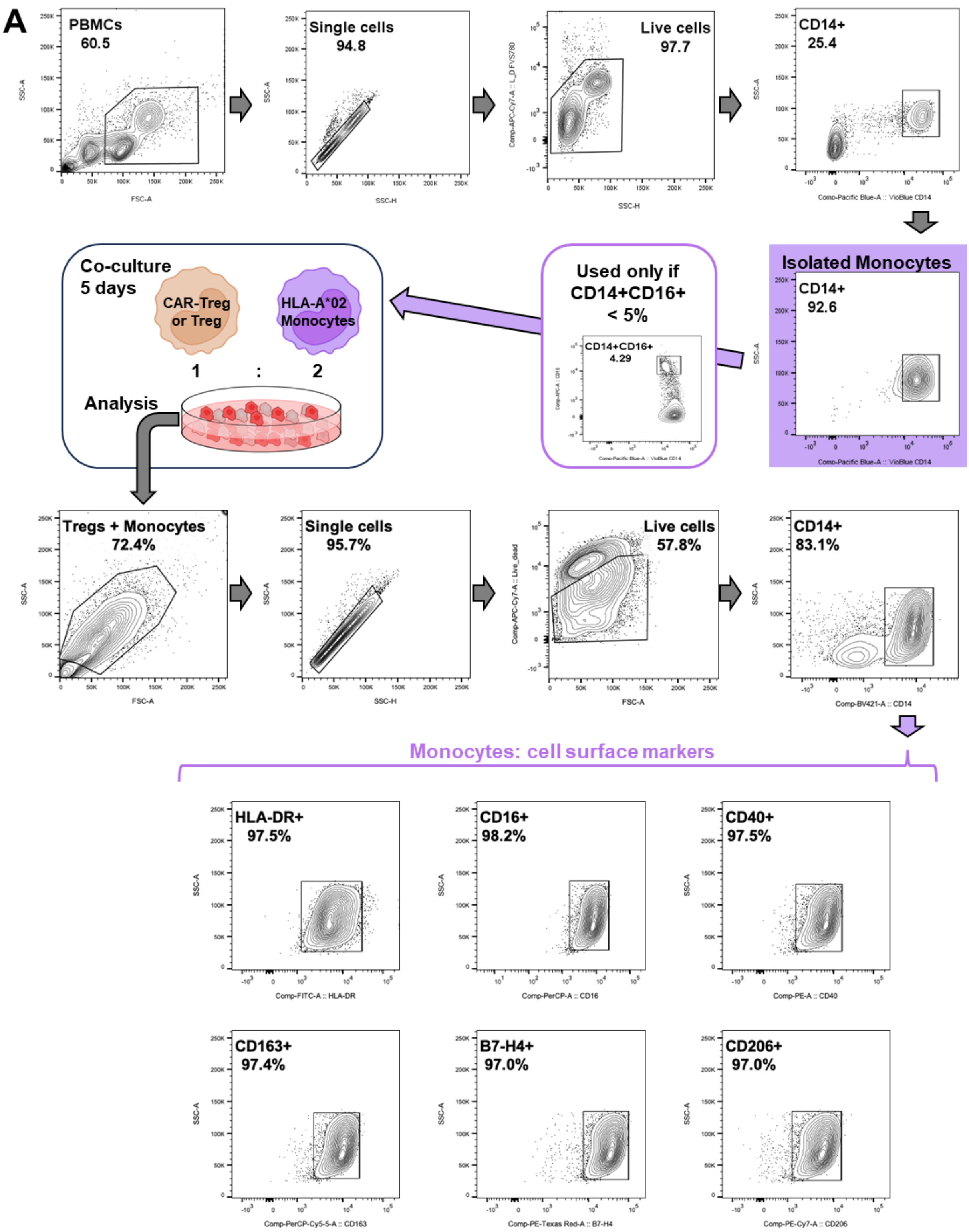
A
PBMCs
60.5
Single cells
94.8
Live cells
97.7
CD14+
25.4
Isolated Monocytes
CD14+
92.6
Used only if
CD14+CD16+
< 5%
CD14+CD16+
4.29
Co-culture
5 days
CAR-Treg
or Treg
HLA-A*02
Monocytes
1
:
2
Analysis
Tregs + Monocytes
72.4%
Single cells
95.7%
Live cells
57.8%
CD14+
83.1%
Monocytes: cell surface markers
HLA-DR+
97.5%
CD16+
98.2%
CD40+
97.5%
CD163+
97.4%
B7-H4+
97.0%
CD206+
97.0%

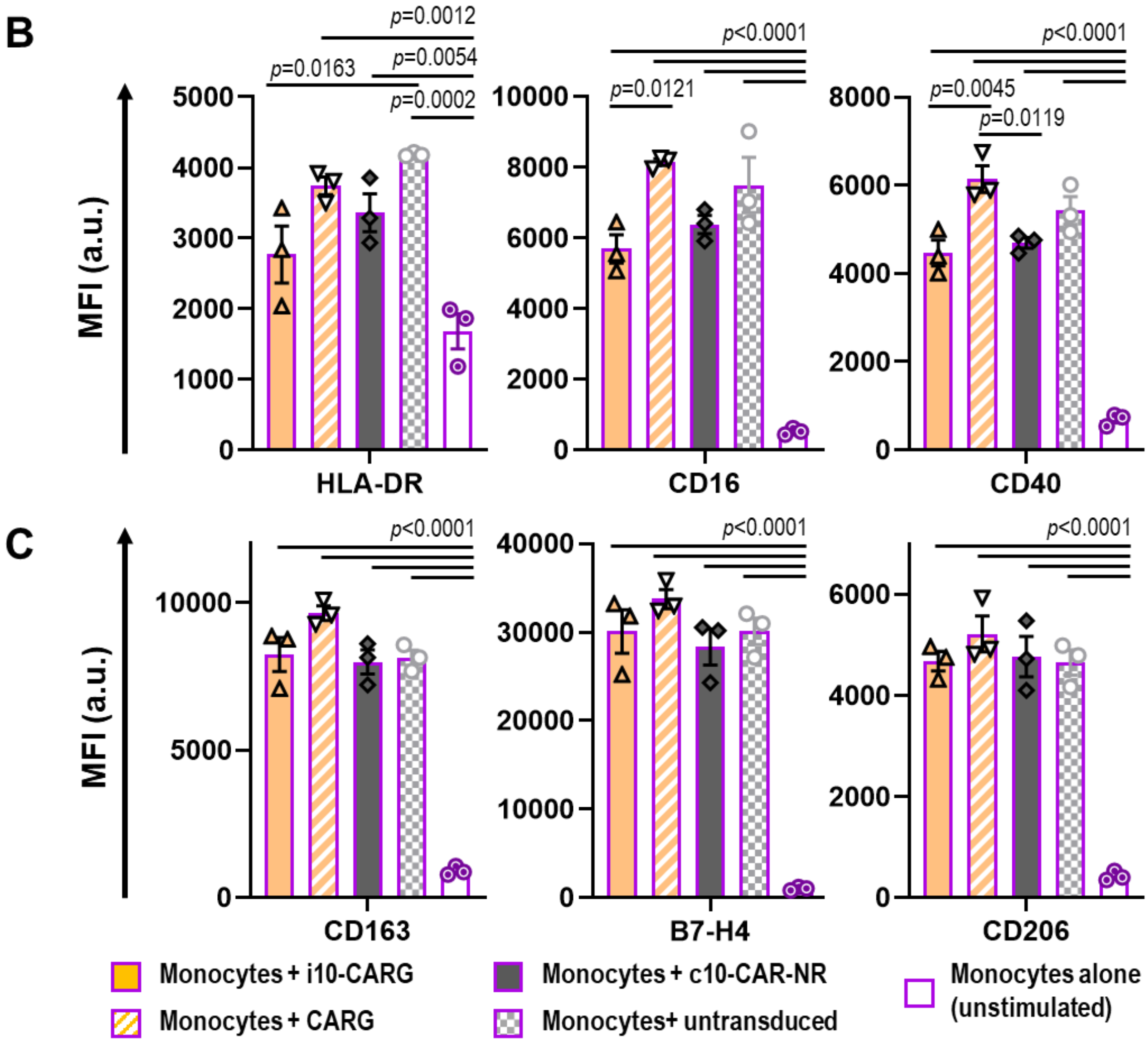

Figure S9. Effect of CAR-Tregs/Tregs on co-cultured HLA-A*02-positive monocytes | (A) Cartoon illustrating the monocyte:CAR-Treg/Treg co-culture assay. Flow cytometry panels at the top exemplify the analysis following the monocyte enrichment process (co-culture input). Flow panels at the bottom illustrate the gating undertaken for the analysis of monocytes after harvesting of the co-cultures. **(B)** Mean fluorescence intensity (MFI) values for indicated pro-inflammatory and **(C)** anti-inflammatory surface markers expressed on HLA-A*02 monocytes following co-culture with indicated Treg types. Unstimulated monocytes were included as a control. Cumulative results are shown from N=3 different experiments (*i.e.* different monocyte and Treg donors). Data are expressed as mean±SEM and with statistical analysis by one-way ANOVA (α<0.05) followed by Tukey's multiple comparison correction.

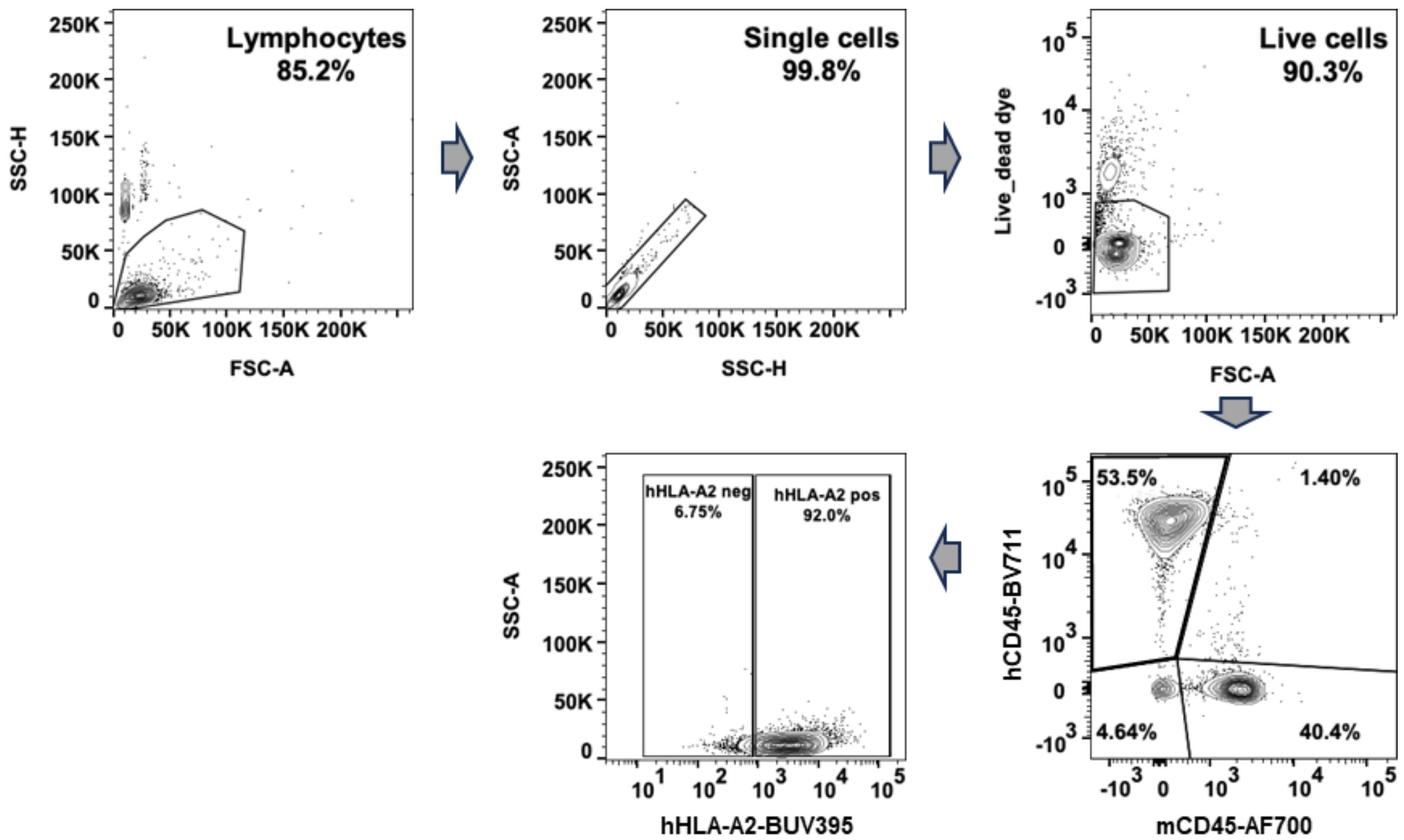

Figure S10. Representative gating strategy for determining HLA-A*02-positive CD45+ cells in peripheral blood of the xeno-GvHD model in Fig.4D. | Sample shown is from an animal that had received PBMCs and i10-CARG Tregs.

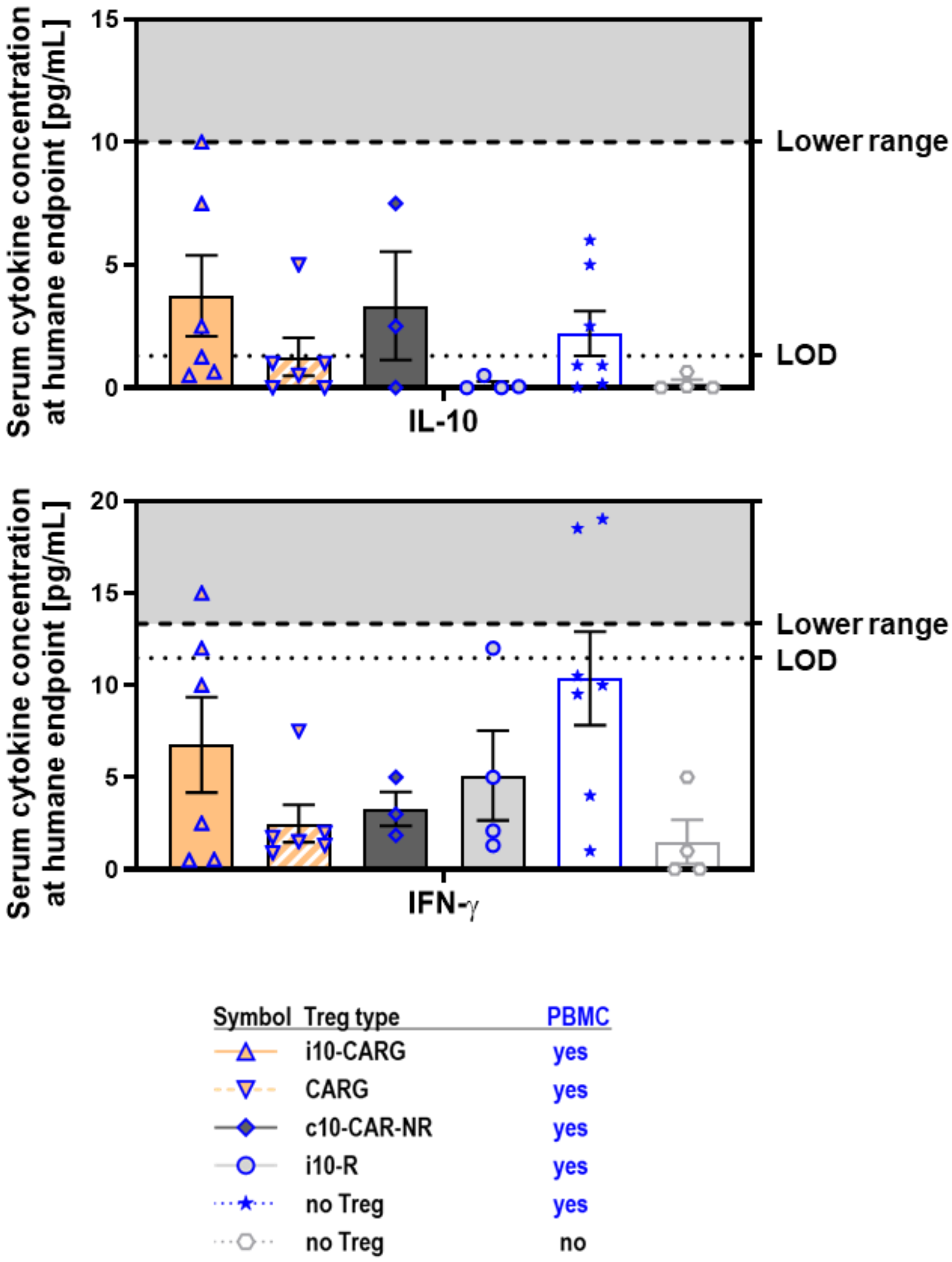

Figure S11. Serum cytokine determination at the time of animal sacrifice (human endpoint) from animals in Fig.4 | Graphs indicate the manufacturer's routine measurement range (*'Lower range' indicates its bottom end; dashed line*) and the limit of detection (*LOD; dotted line*) as obtained by our own measurements in control matrix samples; controls were sera from animals that had neither received PBMCs (no GvHD) nor Tregs and just an injection of saline. The number of data points is lower than in Fig.4, because readings of some serum samples were too low for a quantifiable instrument reading (*'not detectable')*. A noteworthy limitation of these measurements was that sampling was performed at the experimental humane endpoint, i.e. at the time of animal sacrifice, and not repeatedly at different time points ranging from therapeutic cell administration to the humane endpoint. This was due to animal license restrictions, which precluded serial sampling at volumes sufficient for repeat cytokine determination.

SUPPLEMENTAL TABLES

TableS1. List of fluorophores and optical flow cytometry settings as used for routine flow cytometric transduction and expansion analyses of the various Treg types (for constructs see Fig.1A); complements Figure S2 and S3A.

Marker	Fluorescent protein / dye	Antibody clone	Final concentration	Manufacturer	Product code	Laser wave-length (nm)	Emission Filter (nm)
Live/dead stain	DAPI	N/A	0.1µg/mL	ThermoFisher Scientific	62248	355	450/30
eGFP (expressed)	eGFP	N/A	N/A	N/A	N/A	488	530/30
mCherry (expressed)*	mCherry	N/A	N/A	N/A	N/A	561	610/20
mTagRFP (expressed)*	TagRFP	N/A	N/A	N/A	N/A	561	582/15

* never expressed in the same cell type.

TableS2. List of conjugated fluorescent antibodies and dyes as well as optical flow cytometry settings used for phenotype analyses. Fluorescent proteins as expressed by the various Treg types (*cf.* Fig.1A) are included. Antibodies in black font formed the panel for data shown in Fig.1B-D and Figure S4-S5.

Marker	Fluorescent protein / dye	Antibody clone	Dilution	Manufacturer	Product code	Laser wave-length (nm)	Emission Filter (nm)
Live/dead stain	Fixable Near-IR	N/A	1:1000	ThermoFisher Scientific	L10119	640	780/60
eGFP (expressed)	eGFP	N/A	N/A	N/A	N/A	488	530/30
mCherry (expressed)*	mCherry	N/A	N/A	N/A	N/A	561	610/20
mTagRFP (expressed)*	mTagRFP	N/A	N/A	N/A	N/A	561	582/15
CD4	BUV805	SK3	1:33	BD Biosciences	612888	355	780/60
CD25	PE-Vio-770	REA570	1:50	Miltenyi Biotec	130-133-749	561	780/60
CD127	BV786	HIL-7R-M21	1:50	BD Biosciences	563324	405	780/60
FOXP3	BV421	206D	1:20	Biolegend	320124	405	450/40
CD39	BV510	A1	1:20	Biolegend	328220	405	520/50
CD45RA	AF700	HI100	1:50	Biolegend	304120	640	730/45
CD45RO	BUV737	UCHL1	1:33	BD Biosciences	748368	355	740/35
CD62L	BV650	DREG-56	1:20	Biolegend	304832	405	660/20
CLA	BV605	HECA-452	1:20	BD Biosciences	563960	405	610/20
CCR6	BUV496	11A9	1:20	BD Biosciences	612948	355	515/30
CCR7	BV711	150503	1:20	Biolegend	353228	405	710/50
Integrin-β7**	PerCP-Cy5.5	FIB27	1:20	Biolegend	121008	488	710/50

* never expressed in the same cell type.

TableS3. List of conjugated fluorescent antibodies and dyes as well as optical flow cytometry settings used for activation and suppression analyses as in Fig.2A, Fig.3, and Figure S7. Fluorescent proteins as expressed by the various Treg types (*cf.* Fig.1A).

Marker	Fluorescent protein / dye	Antibody clone	Dilution	Manufacturer	Product code	Laser wave-length (nm)	Emission Filter (nm)
CTV	Cell Trace Violet	N/A	1:1000	ThermoFisher Scientific	C34557	405	450/40
Live/dead stain	Fixable Near-IR	N/A	1:1000	ThermoFisher Scientific	L10119	640	780/60
eGFP (expressed)	eGFP	N/A	N/A	N/A	N/A	488	530/30
mCherry (expressed)*	mCherry	N/A	N/A	N/A	N/A	561	610/20
mTagRFP (expressed)*	TagRFP	N/A	N/A	N/A	N/A	561	582/15
CD4	AF647	SK3	1:33	Biolegend	344636	640	670/30
CD25	PE-Vio-770	REA570	1:50	Miltenyi Biotec	130-133-749	561	780/60
CD69	PerCP	FN50	1:20	Biolegend	310928	488	710/50

* never expressed in the same cell type.

TableS4. List of conjugated fluorescent antibodies and dyes as well as optical flow cytometry settings used for monocyte-Treg co-culture experiments as in Figure S9. Fluorescent proteins were only expressed by various Treg types (*cf.* Fig.1A) but not by monocytes, hence are not listed here as CD14 gating always selected the monocyte population prior to cell surface marker analyses.

Marker	Fluorescent protein / dye	Antibody clone	Dilution	Manufacturer	Product code	Laser wave-length (nm)	Emission Filter (nm)
Live/dead stain	Fixable Near-IR	N/A	1:1000	ThermoFisher Scientific	L10119	640	780/60
CD14	BV421	MoP9	1:20	BD Biosciences	563743	405	450/40
HLA-DR	Alexa fluor 488	L243	1:20	Biolegend	307619	488	530/30
CD16	PerCP	3G8	1:20	Biolegend	302029	488	670/30
CD40	PE	5C3	1:20	Biolegend	334307	561	582/15
CD163	PerCP-Vio700	REA812	1:50	Miltenyi Biotec	130-112-291	488	710/50
B7-H4	PE/Dazzle	MIH43	1:20	Biolegend	358112	561	610/20
CD206	PE/Cy7	15-2	1:20	Biolegend	321101	561	780/60

TableS5. List of conjugated fluorescent antibodies and dyes as well as optical flow cytometry settings used for *ex vivo* engraft-ment analyses in peripheral blood as in Fig.4D and Figure S10. Fluorescent proteins as expressed by various Treg types (*cf.* Fig.1A).

Marker	Fluorescent protein / dye	Antibody clone	Dilution	Manufacturer	Product code	Laser wave-length (nm)	Emission Filter (nm)
Live/dead stain	Fixable Near-IR	N/A	1:1000	ThermoFisher Scientific	L10119	640	780/60
eGFP (expressed)	eGFP	N/A	N/A	N/A	N/A	488	530/30
mCherry (expressed)*	mCherry	N/A	N/A	N/A	N/A	561	610/20
mTagRFP (expressed)*	TagRFP	N/A	N/A	N/A	N/A	561	582/15
mCD45	AF700	30-F11	1:50	Biolegend	103128	640	730/45
hCD45	BV711	HI30	1:20	Biolegend	304050	405	710/50
hHLA-A2	BUV395	BB7.2	1:50	BD Biosciences	740313	355	379/28

* never expressed in the same cell type.

TableS6. List of exact *p*-values for individual comparisons in Fig.3B. Exact *p*-values were calculated by two-way ANOVA using Tukey's multiple comparison correction using GraphPad Prism software.

	B-LCL: HLA-A*02		
Tconv:Treg ratio	Tukey's multiple comparisons test	*p*-value	Summary
1	CAR-Treg vs. CAR-IL10i-Tregs	0.6391	ns
	CAR-Treg vs. Group C	0.0071	**
	CAR-IL10i-Tregs vs. Group C	0.0004	***
2	CAR-Treg vs. CAR-IL10i-Tregs	0.3696	ns
	CAR-Treg vs. Group C	0.0183	*
	CAR-IL10i-Tregs vs. Group C	0.0003	***
4	CAR-Treg vs. CAR-IL10i-Tregs	0.4743	ns
	CAR-Treg vs. Group C	0.0304	*
	CAR-IL10i-Tregs vs. Group C	0.0011	**
8	CAR-Treg vs. CAR-IL10i-Tregs	0.9846	ns
	CAR-Treg vs. Group C	0.0042	**
	CAR-IL10i-Tregs vs. Group C	0.0026	**
16	CAR-Treg vs. CAR-IL10i-Tregs	0.946	ns
	CAR-Treg vs. Group C	0.0137	*
	CAR-IL10i-Tregs vs. Group C	0.0057	**
32	CAR-Treg vs. CAR-IL10i-Tregs	0.9814	ns
	CAR-Treg vs. Group C	0.0786	ns
	CAR-IL10i-Tregs vs. Group C	0.116	ns
64	CAR-Treg vs. CAR-IL10i-Tregs	0.9929	ns
	CAR-Treg vs. Group C	0.1171	ns
	CAR-IL10i-Tregs vs. Group C	0.0924	ns
128	CAR-Treg vs. CAR-IL10i-Tregs	0.8198	ns
	CAR-Treg vs. Group C	0.4242	ns
	CAR-IL10i-Tregs vs. Group C	0.7889	ns
256	CAR-Treg vs. CAR-IL10i-Tregs	0.8998	ns
	CAR-Treg vs. Group C	0.8533	ns
	CAR-IL10i-Tregs vs. Group C	0.5955	ns

	B-LCL: HLA-A*01		
Tconv:Treg ratio	Tukey's multiple comparisons test	*p*-value	Summary
all ratios	All comparison had *p*-values	>0.05	ns

SUPPLEMENTAL REFERENCES

1. Jacob, J., Nadkarni, S., Volpe, A., Peng, Q., Tung, S.L., Hannen, R.F., Mohseni, Y.R., Scotta, C., Marelli-Berg, F.M., Lechler, R.I., Smyth, L.A., et al. (2021). Spatiotemporal in vivo tracking of polyclonal human regulatory T cells (Tregs) reveals a role for innate immune cells in Treg transplant recruitment. Mol Ther Methods Clin Dev *20*, 324-336. 10.1016/j.omtm.2020.12.003.
